# Biological Evaluation of a Novel Compound with Predicted EZH2 and EED Binding Against Human Malignant Melanoma Cells

**DOI:** 10.3390/ijms27062647

**Published:** 2026-03-13

**Authors:** Sergei Gorbunov, Sotiris Kyriakou, Ioannis Anestopoulos, Shahzaib Khoso, Marcello Manfredi, Rodrigo Franco, Aglaia Pappa, Mihalis I. Panayiotidis

**Affiliations:** 1 Department of Cancer Genetics, Therapeutics and Ultrastructural Pathology, The Cyprus Institute of Neurology and Genetics, Nicosia 2371, Cyprus; sergeig@cing.ac.cy (S.G.); or mp2358@msstate.edu (M.I.P.); 2 Department of Translational Medicine, Center for Translational Research on Autoimmune and Allergic Diseases, University of Piemonte Orientale, 28100 Novara, Italy; shahzaib.khoso@uniupo.it (S.K.); marcello.manfredi@uniupo.it (M.M.); 3 Institute for Molecular and Translational Cardiology (IMTC), IRCCS Policlinico San Donato, 20097 Milan, Italy; 4 Redox Biology Centre, University of Nebraska-Lincoln, Lincoln, NE 68583, USA; rodrigo.franco@unl.edu; 5 Department of Veterinary Medicine and Biomedical Sciences, University of Nebraska-Lincoln, Lincoln, NE 68583, USA; 6 Department of Molecular Biology and Genetics, Democritus University of Thrace, 68100 Alexandroupolis, Greece; apappa@mbg.duth.gr; 7 Department of Comparative Biomedical Sciences, College of Veterinary Medicine, Mississippi State University, Starkville, MS 39762, USA

**Keywords:** EZH2, EED, PRC2, malignant melanoma, histone methyltransferase, Tazemetostat, H3K27 methylation, blood–brain barrier

## Abstract

Enhancer of Zeste Homolog 2 (EZH2), the catalytic subunit of Polycomb Repressive Complex 2 (PRC2), mediates histone H3 lysine 27 trimethylation (H3K27me3), an epigenetic modification associated with transcriptional repression. EZH2 inhibitors (EZH2is) gained attention after the first-in-class drug Tazemetostat received FDA approval for treating epithelioid sarcoma. Preclinical studies suggest that EZH2is could be effective against melanoma, but their general inability to cross the blood–brain barrier (BBB), among others, limits the treatment of secondary brain metastases. Based on these limitations, we designed SG-8, a novel compound derived from TDI-6118 (a known brain-penetrant EZH2i). In silico docking predicted that SG-8 may exhibit high affinity for EZH2 as well as for another PRC2 subunit, Embryonic Ectoderm Development (EED). In addition, in vitro PAMPA assays suggested passive BBB permeability of SG-8. In cell-based assays, SG-8 and the structurally related EZH2i PF-06726304 displayed lower cytotoxicity than Tazemetostat in both primary (A375) and metastatic (Colo-679) human melanoma cells. Western blot analysis showed that SG-8 and PF-06726304 markedly reduced EED protein levels and, to a lesser extent, EZH2 levels, without affecting total H3K27me3, consistent with preserved canonical PRC2 activity. Instead, treatment with both compounds—most prominently SG-8—was associated with reduced phosphorylation levels of EZH2 (Ser21) and its upstream regulator Akt (Ser473), suggesting that modulation of the Akt–EZH2 signaling axis may at least partially contribute to their anti-melanoma activity.

## 1. Introduction

Deregulations of epigenetic mechanisms (i.e., DNA methylation, histone modifications, chromatin remodeling, and non-coding RNA) are well-known contributors to the development of many multifactorial diseases, including cancer [1,2,3]. Particularly of note, aberrant patterns of histone modifications are implicated in tumorigenesis and are mediated through altered expression levels of specific modifying enzymes (e.g., writers or erasers) [4]. Based on the reversible nature of epigenetic modifications, various chemical entities capable of targeting key epigenetic enzymes (and thus restoring a normal epigenetic landscape) have shown promising results in preclinical models as well as clinical trials. However, only a few of them have been approved by the US Food and Drug Administration (FDA) for clinical use. These so-called epigenetic drugs (epi-drugs) currently fall into three major classes: (i) DNA methyltransferase inhibitors (DNMTi), (ii) histone deacetylase inhibitors (HDACi), and (iii) histone methyltransferase inhibitors (HMTi) [5,6]. Even though epigenetic drugs have shown to be effective against specific types of hematological malignancies, their therapeutic efficacy against solid tumors is rather limited due to high toxicity levels and development of tumor resistance [7,8]. Currently, among different epi-drugs, Tazemetostat (EPZ-6438, E7438) is the only FDA-approved agent for the treatment of solid tumors as monotherapy, specifically for metastatic or locally advanced epithelioid sarcoma [9]. Tazemetostat is an HMTi acting as a selective *S*-adenosyl methionine (SAM)-competitive inhibitor of EZH2 [10].

EZH2 is a member of PRC2 that also contains two other important subunits: (i) Suppressor of Zeste 12 (SUZ12) and (ii) Embryonic Ectoderm Development (EED). In addition, EZH2 is the catalytic subunit of the PRC2 complex that regulates gene expression through its intrinsic histone methyltransferase activity. Specifically, EZH2 is responsible for trimethylation of the ε-amino group of lysine residue 27 in histone H3 (H3K27me3), a modification associated with gene repression. This activity is mediated through the catalytic Su(var)3-9, Enhancer-of-zeste, and Trithorax (SET) domain of EZH2 by using SAM as a methyl group donor. However, to be fully functional, EZH2 requires interaction with the EED and SUZ12 components [11,12].

The first selective EZH2 inhibitor, EPZ005687 (K*_i_* = 24 nM), was discovered through the optimization of a compound identified during high-throughput screening of a library containing 175,000 diverse small molecules [13]. This indazole derivative with a 2-pyridone ring became the prototypical structure for all subsequent SAM-competitive inhibitors of EZH2. Further modifications of the initial structure led to many potent EZH2i, including derivatives of indazole (GSK-343, K*_i_* = 1–1.4 nM), indole (GSK-126, K*_i_* = 0.5–3 nM; GSK-503; EI1; CPI-1205), benzamide (Tazemetostat, K*_i_* = 2.5 nM; EPZ-011989; ZLD1039), and dihydroisoquinolin-1(*2H*)-one (PF-06726304, K*_i_* = 0.7 nM; PF-06821497 (Mevrometostat), K*_i_* < 0.1 nM). These compounds demonstrated particularly strong antiproliferative effects against lymphomas harboring activating EZH2 mutations (Y641N and others), while some, such as Tazemetostat and ZLD1039, also showed efficacy in solid tumors [14,15,16,17,18,19,20].

The limited therapeutic efficacy of selective SAM-competitive EZH2i and the emergence of tumor resistance due to secondary mutations [21,22,23] have driven the development of alternative strategies to suppress PRC2 function. Dual EZH1i/EZH2i, such as UNC1999 and Valemetostat, have shown greater efficacy than selective EZH2i against certain types of leukemia [24,25]. Another promising approach is targeting EED, with two main types of EED inhibitors currently available. The first type includes molecules (EED226, A-395, BR-001, EEDi-5285, EEDi-1056, etc.) that occupy the EED site responsible for recognizing H3K27me3, known as the “aromatic cage” or “top pocket”. These EED allosteric inhibitors act similarly to EZH2i but are also effective against EZH2i-resistant tumors. The second group includes molecules (Astemizole, Wedelolactone, DC-PRC2in-01, etc.) that target the EED “bottom pocket”, which is normally bound to the α-helix of EZH2, thereby disrupting the EZH2–EED protein–protein interaction (PPI) within the PRC2 complex. These EZH2–EED PPIi are distinctive in their ability to accelerate the degradation of PRC2 proteins (EZH2, EED, and SUZ12) [26,27].

Malignant melanoma is the most lethal form of skin cancer with the 5-year survival rate of patients with metastatic melanoma, in the United States, being around 35% [28,29]. Many dysregulated molecular pathways contribute to melanoma pathogenesis, including the MAPK/ERK and PI3K/Akt pathways, among others, that regulate cell cycle control and apoptotic cell death [30]. In this context, despite the development of different therapeutic approaches (e.g., BRAF/MEKi and CTLA-4/PD-1i, among others), survival rates among melanoma patients are still relatively low [28]. On the other hand, malignant melanoma is also characterized by a deregulated epigenetic landscape that further contributes to its pathophysiology. In this context, it has been reported that EZH2 plays a crucial role in melanoma development since its overexpression is associated with increased metastatic rates and poor progression among melanoma patients. In addition, melanoma cells often carry point mutations in the SET domain of EZH2, such as Y646*, responsible for its increased enzymatic activity and consequently upregulated levels of H3K27me3 [31]. Several studies have demonstrated that inhibiting EZH2 can suppress tumor growth and metastatic potential in melanoma [31,32,33,34]. Tazemetostat has shown selective toxicity against both primary (A375) and metastatic (Colo-679) melanoma cells, but is relatively non-toxic in non-malignant, immortalized keratinocyte (HaCaT) cells [35]. These findings suggest that EZH2i hold great promise as therapeutic agents for the treatment of malignant melanoma.

However, a great limitation of currently utilized therapeutic agents is their poor efficacy in crossing the blood–brain barrier (BBB). Over 10% of all cancer patients, including 7% of melanoma patients, will develop secondary brain metastases [36]. Interestingly, EZH2 is a key factor in the survival of brain tumors like midline glioma [37,38], ependymoma [39], and atypical rhabdoid tumors [40,41]. Thus, brain-penetrant EZH2i hold great promise as effective agents in the treatment of advanced stages of melanoma and possibly other cancers as well. However, Tazemetostat (as the majority of EZH2i) has a modest capacity to reach the central nervous system due to the presence of active drug efflux transporters like P-glycoprotein (P-gp, ABCB1, MDR1) and BCRP/ABCG2 (a breast cancer resistance protein) [42]. On the other hand, TDI-6118 has been reported as an EZH2i with high efficacy in crossing the BBB, but with relatively low potency [43].

Over the past decades, in silico (computer-based) modeling has become an invaluable tool for accelerating and optimizing drug discovery [44]. Molecular docking is widely used to predict the preferred orientation and binding affinity between small molecules and target proteins. This technique has also been particularly useful in identifying new compounds targeting various epigenetic mechanisms [45,46,47]. Notably, computer modeling played a central role in the discovery of TDI-6118, the first brain-penetrant EZH2 inhibitor [43]. In addition, pharmacokinetic calculations, such as those assessing bioavailability and blood–brain barrier permeability, have achieved strong predictive power and are now routinely utilized in medicinal chemistry studies [48,49].

Overall, brain-penetrant EZH2 inhibitors hold significant therapeutic potential but remain underdeveloped. In this study, we aim to advance this field by designing a novel compound based on the previously known TDI-6118. Our initial goal was to develop a new molecule with predicted EZH2 binding efficacy while maintaining physicochemical properties compatible with BBB permeability. In this context, we next assessed: (i) cytotoxicity (as a means of therapeutic efficacy), (ii) capacity to target members of the PRC2 complex, (iii) ability to modulate H3K27me3 marks, and (iv) effects on the EZH2 phosphorylation status of our newly synthesized compound (SG-8) against primary (A375) as well as metastatic (Colo-679) human malignant melanoma cells.

## 2. Results

### 2.1. In Silico Analyses of Various EZH2i Molecules Under Study

**Figure 1:**
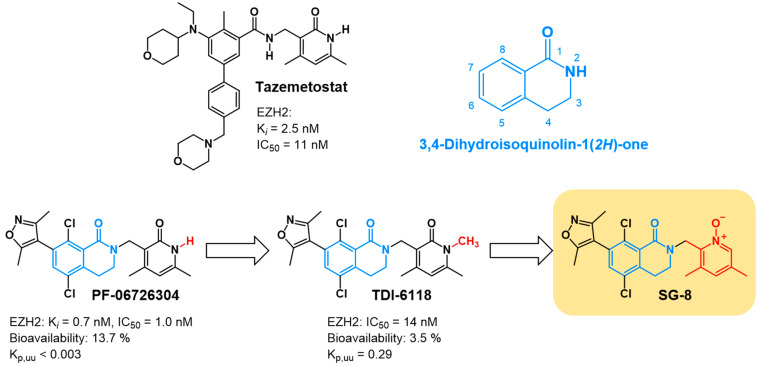
Design of SG-8 based on the chemical structure of TDI-6118, which originates from the lead compound PF-06726304. SG-8 was designed as a bioisosteric analog of TDI-6118. PF-06726304, TDI-6118, and SG-8 can be classified as 3,4-dihydroisoquinoline-1(*2H*)-one derivatives based on their common core structure. K*_i_*—inhibition constant; IC_50_—half-maximal inhibitory concentration; K_p,uu_—unbound brain-to-plasma partition coefficient. Blue color indicates the 3,4-dihydroisoquinolin-1(*2H*)-one core structure, red color highlights the modified substituent groups, and black represents the remaining parts of the molecule.

To explore possible strategies for improving TDI-6118, we designed and synthesized a new small molecule, SG-8, based on the chemical structure of TDI-6118, with the anticipated ability to target EZH2 and cross the BBB (Figure 1).

The potential inhibitory properties of SG-8 were estimated by computational chemistry methods. First, molecular docking analysis was performed to calculate the binding affinity (ΔG°) of the SG-8 ligand for the SET domain of EZH2 (Table 1, Figures S19–S21). For this purpose, a 3D structure of the EZH2 protein (*Homo sapiens*/*Anolis carolinensis* chimera, wild type, chain B) was isolated from the X-ray crystallography data containing the PRC2 complex with a structurally related 3,4-dihydroisoquinoline-1(*2H*)-one derivative (PDB ID: *5IJ7*). The same EZH2 protein structure was previously utilized for successful in silico calculations of TDI-6118 inhibitory properties, confirming its suitability for this type of modeling [43]. The ΔG° value was converted to the inhibition constant (K*_i_*) (Equation (1)) to compare the potency of SG-8 with the known EZH2 inhibitors.

(1) $$K_{i}\;or\;K_{d}=exp\left(\frac{∆G{}^{\circ}}{RT}\right),\;\;R=1.9872\times 10^{-3}\;kcal\cdot K^{-1}\cdot {mol}^{-1},\;\;T=298.15\;K$$

To validate the in silico docking model, we calculated the corresponding ΔG° and K*_i_* values of both PF-06726304 and TDI-6118 compounds, for which the experimental K*_i_* values were taken from the literature [18,43] (Table 1).

**Table 1:** Results of molecular docking. Binding affinities were calculated as standard Gibbs free energies ΔG° (kcal/mol) of the ligand/protein interaction. Adjusted K*_i_* values were obtained after division of the AutoDock-calculated K*_i_* by the common coefficient 3.4.

Target	Quantity	Unit	PF-06726304	TDI-6118	SG-8
EZH2	Calculated ΔG°	kcal/mol	−11.77	−10.22	−11.36
	Calculated * K*_i_*	nM	2.38	32.20	4.69
	Adjusted K*_i_*	nM	0.7	9.5	1.4
	Experimental K*_i_*	nM	0.7 [18]	10 **	–
	Experimental IC_50_	nM	1.0 ± 0.2 [43]	14 ± 3 [43]	–
EED	Calculated ΔG°	kcal/mol	−11.10	−10.20	−10.88
	Calculated * K*_d_*	nM	7.34	33.32	10.64
SUZ12	Calculated ΔG°	kcal/mol	−5.84	−5.94	−6.01
	Calculated * K*_d_*	nM	5.24 × 10^4^	4.44 × 10^4^	3.93 × 10^4^

K*_i_*—inhibition constant characterizing the degree of dissociation of the inhibited ligand/enzyme complex. K*_d_*—dissociation constant, which is a broader interpretation of K*_i_* for any ligand/protein interaction. * K*_i_* and K*_d_* values were calculated from the corresponding ΔG° (kcal/mol) using Equation (1). ** Experimental K*_i_* for TDI-6118 was estimated based on the experimental IC_50_ values of TDI-6118 and PF-06726304 and the experimental K*_i_* value of PF-06726304, using the following proportion derived from the Cheng–Prusoff equation [50] (Equation (2)):

(2) $$\frac{K_{i}\left(X\right)}{K_{i}\left(Y\right)}=\frac{{IC}_{50}\left(X\right)}{{IC}_{50}\left(Y\right)}$$

Although the calculated K*_i_* values for both PF-06726304 and TDI-6118 were approximately three times lower than the experimental values, the model accurately predicted the relative potency of PF-06726304 in comparison to TDI-6118. The calculated K*_i_* value for TDI-6118 was 13.5 times higher than that of PF-06726304, whereas the ratio between the experimental K*_i_* values for these compounds was 14:1, respectively. Based on the above validations, we concluded that our in silico model consistently makes the same error when calculating EZH2 affinity values for all bioisosteric analogues of PF-06726304 or TDI-6118, leading to an overestimation of the calculated K*_i_* values by a factor of 3.4. Regarding ΔG°, this error is equivalent to an increase of +0.725 kcal/mol. To obtain a more accurate assessment of SG-8 potency, we adjusted our calculated K*_i_* values by dividing them by 3.4 (Table 1). The validity of this adjustment is supported by the observation that docking-derived scores often require post-processing to correct systematic offsets and more accurately approximate absolute K*_i_* values [51]. Additionally, applying an empirical scaling factor or linear regression can significantly enhance the agreement between docking predictions and in vitro K*_i_* values [52]. The docking results for SG-8 suggested a high ability of this compound to inhibit EZH2. According to the calculated K*_i_* values, SG-8 appeared to be less potent than PF-06726304 but significantly more potent than TDI-6118. After adjustment, SG-8 revealed a K*_i_* = 1.4 nM and was therefore predicted to be more potent than Tazemetostat (K*_i_* = 2.5 nM).

In silico analysis of local interactions between the SG-8 ligand and EZH2 (Figure S20) suggested that SG-8 occupies the same binding site within the SET domain as PF-06726304 (Figure S19), predominantly in the same position. Our prediction of the preferred orientation of PF-06726304 is consistent with existing X-ray data for this and related compounds (PDB ID: *6B3W*, *5IJ7*). A strong hydrogen-bond interaction with Tyr111 and favorable π–π contacts with aromatic residues such as Phe665, Phe686, and Tyr661 were predicted for both PF-06726304 and SG-8. However, in contrast to PF-06726304, SG-8 did not show any hydrogen bonds with Trp624. Instead, an ionic attraction between the oxygen atom of the *N*-oxide group and the Arg685 residue was suggested.

**Figure 2:**
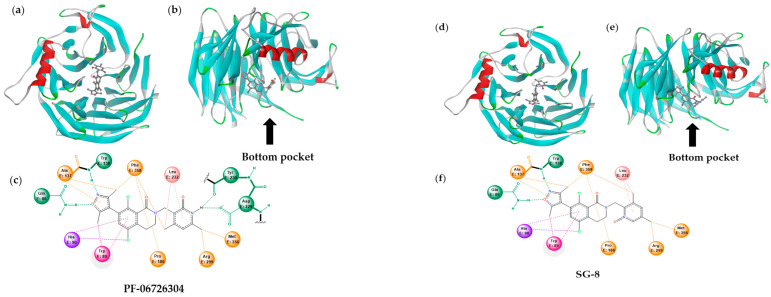
Docking calculations predicting interactions of PF-06726304 (**a**–**c**) and SG-8 (**d**–**f**) with the bottom pocket of EED. The binding of the ligands to the entire EED subunit can be observed from the front (**a**,**d**) and side (**b**,**e**). (**c**,**f**) represent schematically the local interactions of the ligands with the amino acid residues in the binding site (chain E). PF-06726304 appears to establish hydrogen bonds with Asp229, Tyr230, Gln86, and Trp138 (green dashed lines). Additionally, π–π stacking (Trp89) and several hydrophobic interactions contribute to the overall binding (other dashed lines). In contrast, SG-8 does not form hydrogen bonds with Asp229 and Tyr230. The wavy line indicates a truncated part of the structure.

To assess any potential multitarget effects, we also investigated the interactions between our inhibitors and the other subunits of the PRC2 complex, namely SUZ12 (PDB ID: *5IJ7*, *Homo sapiens*, wild type, chain S) and EED (PDB ID: *5IJ7*, *Homo sapiens*, wild type, chain E) (Table 1). The same molecular docking methodology used for EZH2 was also applied to identify specific interaction sites on these subunits and calculate the corresponding binding affinities (ΔG°). Dissociation constants (K*_d_*) were obtained from the ΔG° values according to Equation (1). Based on our analysis, neither SG-8 nor PF-06726304 nor TDI-6118 exhibited any significant affinity for SUZ12. However, all these compounds were predicted to have strong interactions with EED (in a nanomolar range of concentrations). Specifically, PF-06726304 showed a stronger calculated affinity (in terms of a lower K*_d_* value) compared to SG-8, while all appeared to bind to the same EED binding site at similar positions (Figure 2 and Figure S18). In general, EED has a β-propeller architecture consisting of seven β-sheets arranged around a central pore. The binding site was found in this central pore at the side which is closer to the α-helix of EED (bottom pocket).

Unlike EZH2 inhibition, where the 2-pyridone cycle of PF-06726304 serves as a key pharmacophore facilitating critical hydrogen bonding, interaction with EED appears to rely on the opposite part of the molecule—an isoxazole ring (Figure 2). According to our in silico data, this isoxazole fragment in both PF-06726304 and SG-8 may form strong hydrogen bonds with Gln86 and Trp138 of EED. Additional hydrogen bonding with Asp229 and Tyr230, observed for PF-06726304, seems less critical, as its absence in SG-8 resulted in only a minor increase in calculated K*_d_*.

**Figure 3:**
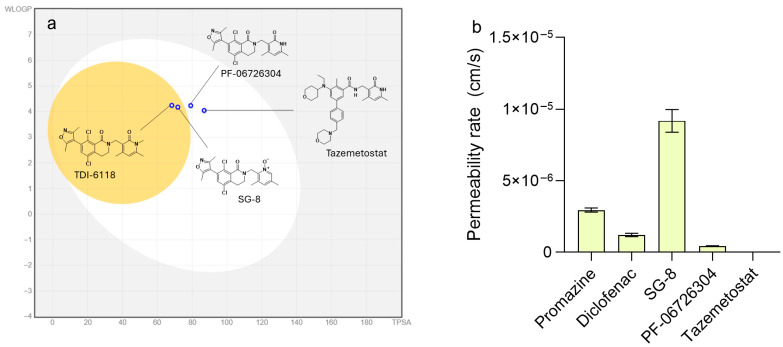
(**a**) BOILED-Egg plot represents the calculated capacity of a compound to be absorbed from the gastrointestinal tract (GI) and passively permeate through the BBB. The vertical axis corresponds to hydrophobicity (WlogP; calculated by the atomistic method of Wildman & Crippen [53]). The horizontal axis shows the predicted topological polar surface area (TPSA, Å^2^) of a molecule [54]. This BOILED-Egg plot was constructed using the SwissADME website (http://www.swissadme.ch/) [48,49]. The white region represents compounds with a high probability of having good GI absorption, whereas the yellow circle (yolk) corresponds to molecules that are likely to cross the BBB. (**b**) PAMPA in vitro assay results for passive BBB permeability. Effective permeability rates (P_e_, cm/s) were calculated using the following equation (Equation (3)) [55]:

Finally, several physicochemical properties of SG-8 (in addition to Tazemetostat, PF-06726304, and TDI-6118) were calculated to estimate its potential ability to cross the BBB (Table S1). The BOILED-Egg model predicted that SG-8 may not only be well absorbed in the gastrointestinal tract but also have the potential to reach the central nervous system (Figure 3a). The predictive performance of this model was benchmarked using two non-BBB-penetrant compounds (Tazemetostat and PF-06726304) as negative controls together with a BBB-penetrant compound (TDI-6118) as a positive control.

(3) $$P_{e}=-\frac{V_{D}\times V_{A}}{\left(V_{D}+V_{A}\right)\times S\times t}\times \ln\left(1-\frac{C_{A}}{C_{E}}\right)=-7.72\times 10^{-6}\times \ln\left(1-\frac{C_{A}}{200\;\mu M}\right)$$

where C_A_ is the resulting molar concentration in the acceptor solution; C_E_ is the theoretical equilibrium concentration, equal to 200 μM; the donor volume (V_D_) is 0.2 cm^3^, the acceptor volume (V_A_) is 0.3 cm^3^, the membrane area (S) is 0.24 cm^2^, and the incubation time (t) is 64,800 s (18 h). Promazine was used as a positive control, representing a compound with high brain penetrance, whereas diclofenac served as a negative control.

### 2.2. In Vitro Assessment of Passive Blood–Brain Barrier Permeability of SG-8

To further assess the potential BBB permeability of SG-8, an in vitro evaluation was conducted using the Parallel Artificial Membrane Permeability Assay (PAMPA) [56]. The results demonstrated that SG-8 possesses a remarkably high capacity for passive diffusion across the artificial BBB, exhibiting a permeability rate (P_e_ = 9.2 × 10^−6^ cm/s) approximately three times higher than that of the high-permeability control, promazine (P_e_ = 2.9 × 10^−6^ cm/s). In contrast, PF-06726304 displayed lower permeability than the low-permeability control, diclofenac, while Tazemetostat showed no detectable membrane penetration (Figure 3b). These findings are fully consistent with our computational predictions and agree with previously published data [42,43].

### 2.3. Differential Cytotoxic Response Induced by SG-8 and PF-06726304

**Figure 4:**
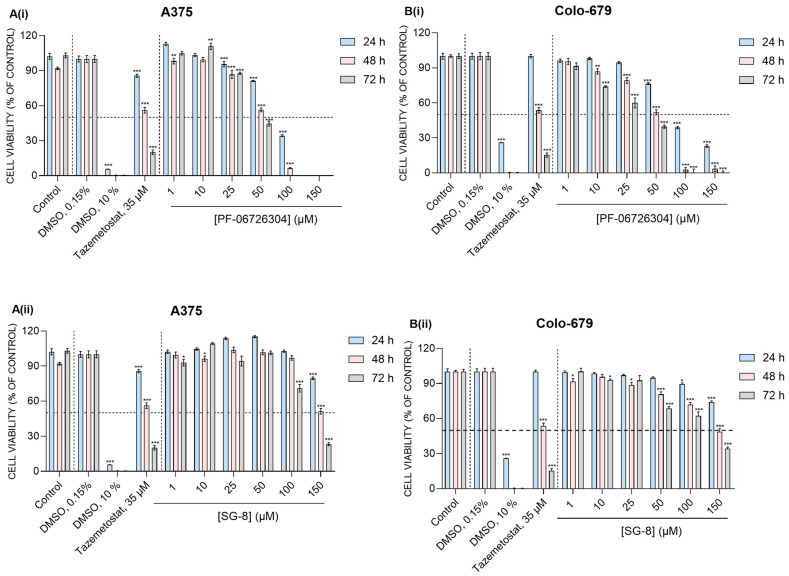
Cytotoxic profile of SG-8 and PF-06726304 against A375 and Colo-679 cells exposed to increasing concentrations of either compound (1–150 μM) over 24–72 h. (**A(i)**) Viability of A375 cells treated with PF-06726304 for 24, 48, and 72 h. (**A(ii)**) Viability of A375 cells treated with SG-8 for 24, 48, and 72 h. (**B(i)**) Viability of Colo-679 cells treated with PF-06726304 for 24, 48, and 72 h. (**B(ii)**) Viability of Colo-679 cells treated with SG-8 for 24, 48, and 72 h. In parallel, Tazemetostat (35 μM) and DMSO (10%) were used as positive controls, while DMSO (0.15%) was used as a negative control. Cell viability levels were determined through the Alamar Blue assay. Data are presented as means ± SEM and are representative of three independent experiments. Each dashed line indicates the 50% cell viability threshold. Statistical significance was set at * *p* < 0.05, ** *p* < 0.01, *** *p* < 0.001 compared with 0.15% DMSO.

Next, we assessed the cytotoxic efficacy of SG-8 together with that of PF-06726304 in primary (A375) and metastatic (Colo-679) human malignant melanoma cells. Cytotoxicity was evaluated by the Alamar Blue assay as previously described [35,57,58]. Specifically, A375 and Colo-679 cells were exposed to increasing concentrations (1–150 μM) of either SG-8 or PF-06726304 over 24–72 h. In parallel, cells were exposed to Tazemetostat (35 μM) and DMSO (10%), both of which were used as positive controls (Figure 4). Finally, half-maximal effective concentrations (EC_50_) for each compound were calculated (Table 2).

Overall, both SG-8 and PF-06726304 appeared to be less potent in decreasing viability levels of A375 and Colo-679 melanoma cells compared to Tazemetostat. Specifically, PF-06726304 appeared to be the most potent, with EC_50_ values reaching 50 μM, while SG-8 was less potent, with EC_50_ values of approximately 150 μM after 48 h of exposure. At the same time, PF-06726304 and SG-8 caused distinct morphological changes in A375 cells, characterized by cell elongation and branching of cell protrusions, which were neither evident in Colo-679 cells nor observed under exposure to Tazemetostat (Figure S22).

### 2.4. Alterations in Protein Expression Levels of PRC2 Complex Members, H3K27me3 Marks, and EZH2 Phosphorylation Induced by SG-8 and PF-06726304

In this set of experiments, we examined the effect of SG-8, along with PF-06726304 and Tazemetostat (used as a reference EZH2 inhibitor), on the expression levels of EZH2, EED, SUZ12, and total H3K27me3 marks. A375 cells were exposed to the respective EC_50_ concentrations of (i) Tazemetostat (35 μM), (ii) PF-06726304 (50 μM), and (iii) SG-8 (150 μM) for 48 h.

All three compounds decreased EZH2 protein levels to a variable extent, with SG-8 causing the most pronounced reduction. SUZ12 expression levels remained unchanged across all treatments. In contrast, EED expression was strongly reduced by SG-8; PF-06726304 showed a milder decrease, while Tazemetostat exerted only a modest effect.

**Figure 5:**
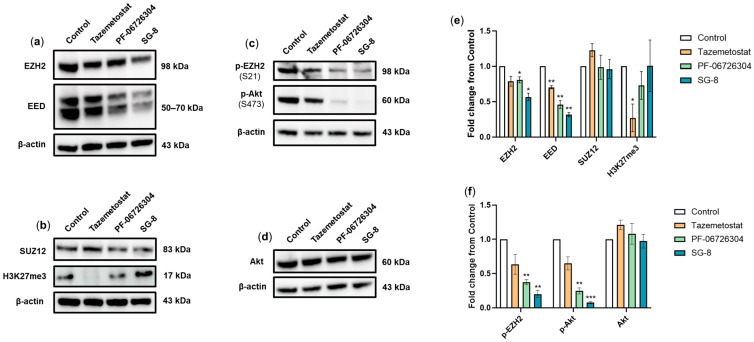
Effects of Tazemetostat, PF-06726304, and SG-8 on PRC2 complex components and EZH2-related signaling in A375 melanoma cells. Cells were treated with the EC_50_ concentrations of (i) Tazemetostat (35 μM), (ii) PF-06726304 (50 μM), or (iii) SG-8 (150 μM) for 48 h. Whole-cell extracts were subjected to immunoblot analysis for (**a**) EZH2 and EED, and (**b**) SUZ12 and the histone mark H3K27me3. Panels (**c**,**d**) show phosphorylation levels of EZH2 and its upstream regulator Akt. Equal protein loading was confirmed by β-actin re-probing. Western blots shown are representative of three independent experiments. Densitometric quantification of protein bands (**e**,**f**) is presented as means ± SEM. Statistical significance was set at * *p* < 0.05, ** *p* < 0.01, and *** *p* < 0.001.

H3K27me3 expression levels were shown to be differentially altered. Specifically, Tazemetostat almost completely abolished this post-translational modification, whereas PF-06726304 and SG-8 did not reduce H3K27me3 levels. Notably, despite being the most effective in decreasing EZH2 and EED expression levels, SG-8 treatment was associated with a slight increase in H3K27me3 levels (Figure 5a,b,e).

In addition to analyzing PRC2 core components, we evaluated whether our compounds affected key regulatory post-translational modifications. Previous studies have shown that phosphorylation of EZH2 at serine 21 markedly reduces its affinity for histone H3, thereby lowering H3K27 trimethylation levels. This modification is mediated by Akt when it is activated through phosphorylation at serine 473 [59]. Accordingly, we assessed EZH2 phosphorylation (p-EZH2, Ser21) and examined its upstream regulator Akt by measuring both total Akt and phosphorylated Akt (p-Akt, Ser473).

Treatment with SG-8 markedly reduced p-EZH2 (Ser21) levels, accompanied by a decrease in p-Akt (Ser473) expression. In addition, PF-06726304 caused a similar but less pronounced effect. In contrast, Tazemetostat had minimal influence on the phosphorylation of either EZH2 or Akt, while total Akt protein levels were not altered by any treatment, indicating that the compounds primarily affected phosphorylation rather than overall protein abundance (Figure 5c,d,f).

### 2.5. Impact of PF-06726304 and SG-8 on Cell Cycle Progression

**Figure 6:**
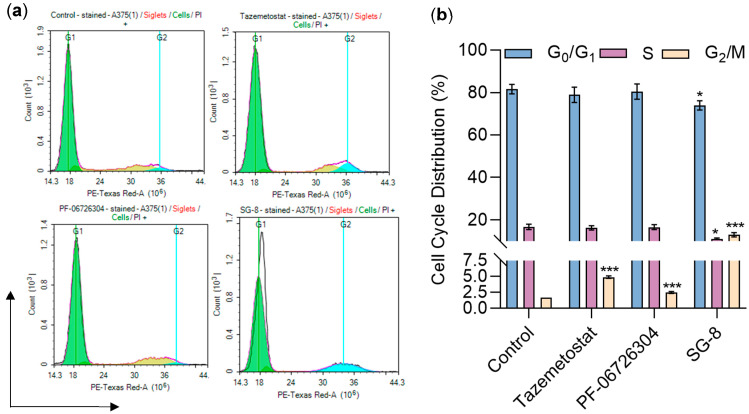
Effects of Tazemetostat (35 μM), PF-06726304 (50 μM), and SG-8 (150 μM) on cell cycle progression in A375 cells after 48 h of exposure. (**a**) Flow cytometry analysis using PI staining, with fluorescence measured in the PE-Texas Red channel; The x-axis represents propidium iodide (PI) fluorescence intensity (DNA content), and the y-axis indicates the number of counted cells (events). Green, yellow, and cyan peaks correspond to cell populations in the G_0_/G_1_, S, and G_2_/M phases of the cell cycle, respectively. (**b**) Cell cycle phase distributions are presented as percentages (Table S3). Statistical significance was set at * *p* < 0.05, *** *p* < 0.001.

To assess whether PF-06726304 or SG-8 interfered with cell cycle progression, we performed flow cytometry analysis on A375 cells using propidium iodide (PI) staining. The cells were exposed to the corresponding EC_50_ concentrations for 48 h: 50 μM of PF-06726304, 150 μM of SG-8, and 35 μM of Tazemetostat (used as a positive control). SG-8 induced pronounced alterations in cell cycle distribution, characterized by a significant decrease in the percentage of cells in the S phase and a concomitant accumulation in the G_2_/M phase compared to untreated controls. PF-06726304 also affected cell cycle progression, causing a reduction in the G_2_/M phase population, although to a lesser extent than SG-8. Notably, both SG-8 and PF-06726304 reduced the proportion of cells in the G_2_/M phase. In contrast, Tazemetostat treatment led to a modest decrease in the S phase population without significantly affecting other cell cycle phases (Figure 6, Table S3).

### 2.6. Impact of PF-06726304 and SG-8 on Apoptosis Induction

Finally, induction of apoptotic cell death was evaluated in A375 cells treated with PF-06726304 (50 μM), SG-8 (150 μM), or Tazemetostat (35 μM) for 48 h. Flow cytometric analysis was performed following dual staining with Annexin V and propidium iodide (PI). In this assay, Annexin V detects both early and late apoptotic cells by binding to phosphatidylserine (PS), which becomes externalized to the outer leaflet of the plasma membrane during apoptosis. In contrast, PI is a DNA-intercalating agent that penetrates only cells with compromised membrane integrity, thereby identifying cells undergoing late apoptosis or necrosis. Thus, this combination not only enables the detection of apoptotic cells but also allows for discrimination between early (Annexin V^+^/PI^−^) and late apoptotic (Annexin V^+^/PI^+^) cell populations.

**Figure 7:**
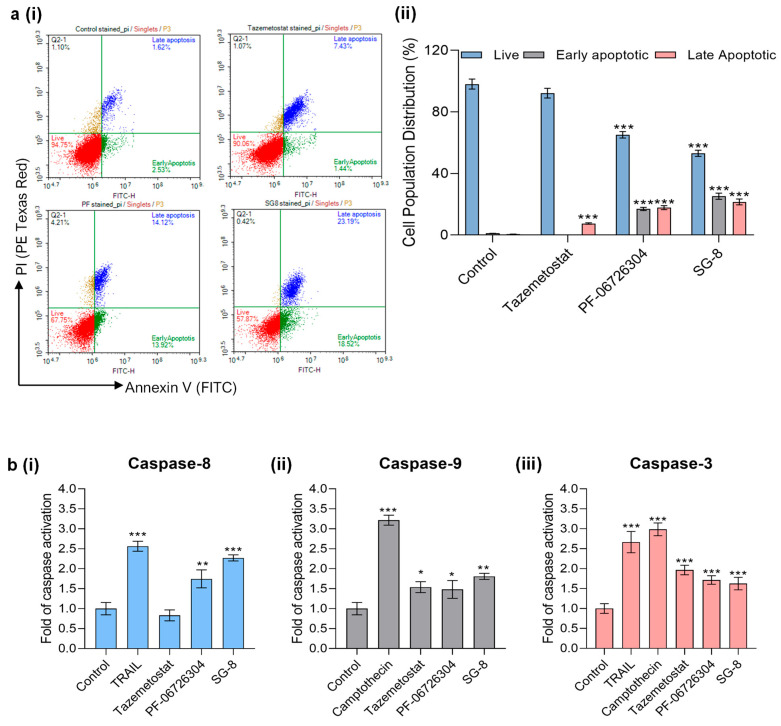

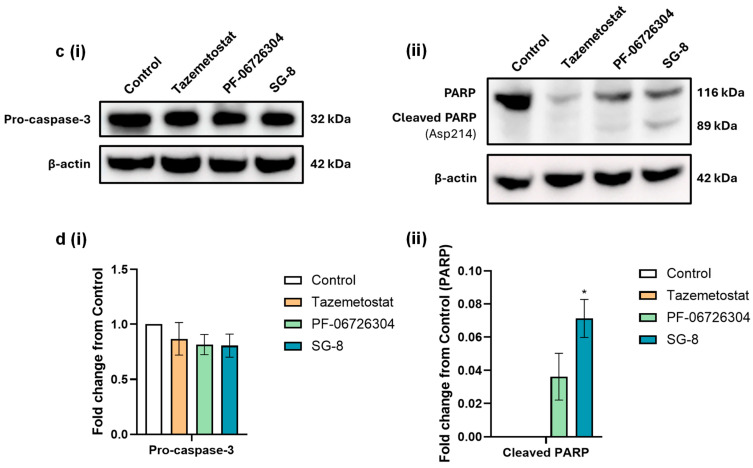
Assessment of apoptosis activation in A375 cells following 48 h exposure to Tazemetostat (35 μM), PF-06726304 (50 μM), and SG-8 (150 μM). (**a**) Flow cytometric analysis following Annexin V/propidium iodide (PI) staining was performed to quantify early and late apoptotic cell populations: (**i**) representative dot plots and (**ii**) corresponding quantification of early and late apoptotic cells. In the dot plots, red dots represent viable cells (Annexin V^−^/PI^−^), green dots correspond to early apoptotic cells (Annexin V^+^/PI^−^), blue dots indicate late apoptotic cells (Annexin V^+^/PI^+^), and brown dots represent necrotic cells (Annexin V^−^/PI^+^). The x-axis shows Annexin V fluorescence (FITC channel), while the y-axis represents PI fluorescence measured in the PE–Texas Red channel. (**b**) Relative enzymatic activity levels of caspases in treated A375 cells: (**i**) caspase-8, (**ii**) caspase-9, and (**iii**) caspase-3. Tumor necrosis factor (TNF)-related apoptosis-inducing ligand (TRAIL) was utilized as a positive control for a caspase-8-driven extrinsic apoptotic pathway, while Camptothecin was used for a caspase-9-driven intrinsic one. (**c**) Western blot analysis of (**i**) pro-caspase-3 and (**ii**) PARP (total and cleaved forms) protein expression levels. (**d**) Corresponding densitometric quantification of (**i**) pro-caspase-3 and (**ii**) cleaved PARP (normalized to total PARP) expression levels. Western blots shown are representative of three independent experiments. Data are expressed as percentages and/or fold changes relative to control and presented as means ± SEM, representative of three independent experiments. Statistical significance was set at * *p* < 0.05, ** *p* < 0.01, *** *p* < 0.001.

According to our results, both PF-06726304 and SG-8 showed significant induction of apoptosis, with early apoptotic cells (Annexin V^+^/PI^−^) represented at levels comparable to those observed in late apoptotic cells (Annexin V^+^/PI^+^). In contrast, Tazemetostat exhibited a lower overall apoptotic effect, with apoptosis predominantly detected at the late stage (Figure 7a).

Additionally, the enzymatic activities of caspase-3 (executioner caspase), caspase-8 (extrinsic pathway), and caspase-9 (intrinsic pathway) were assessed. Our data revealed differential activation of all examined caspases depending on the treatment. Specifically, SG-8 was the most potent inducer of both caspases-8 and -9 activity compared to either PF-06726304 or Tazemetostat. In particular, SG-8-induced caspase-8 activity levels were comparable to the effect of TRAIL, used as a positive control. On the other hand, a significant induction of caspase-9 activity was also observed, with SG-8 being slightly more potent compared to PF-06726304 and Tazemetostat. Finally, increased levels of caspase-3 activity were observed after treatments with all tested compounds, with Tazemetostat appearing as the most potent one. These results indicate, at least partially, the concomitant activation of both extrinsic and intrinsic apoptotic cascades as an underlying mechanism of cell death induction (Figure 7b).

Finally, we have assessed the activation of apoptosis by examining the protein expression levels of caspase-3 and poly (ADP-ribose) polymerase (PARP; total and cleaved forms) through Western blot analysis (Figure 7c). All tested compounds induced a slight reduction in pro-caspase-3 levels in A375 cells, while cleaved caspase-3 expression remained below detectable levels under the applied experimental conditions. In contrast, a slight cleavage of PARP was detected after treatment with PF-06726304, while exposure to SG-8 caused a statistically significant PARP cleavage, as evidenced by immunoblot analysis (Figure 7c). PARP is a well-established substrate of caspase-3, and its proteolytic cleavage is widely recognized as a hallmark of apoptosis, thereby supporting the induction of apoptotic cell death under the above conditions.

Overall, all applied methodologies, including Annexin V/PI flow cytometry, caspase enzymatic activity assays, and Western blot analysis, consistently support the induction of apoptosis in A375 cells following treatment with PF-06726304 and SG-8.

## 3. Discussion

Epigenetic alterations play a pivotal role in the development of cancer, including melanoma [1,2,3,4]. EZH2 is a key histone methyltransferase involved in gene repression through deposition of H3K27me3 marks [11,12], while its overexpression has been linked to various characteristics of melanoma cells such as progression, metastasis, as well as resistance to both immuno- and targeted therapeutic agents [31,32]. However, despite the development of several EZH2 inhibitors (e.g., GSK-126 and Tazemetostat), their low rates of crossing the BBB [42] may hinder the treatment of melanoma cases involving secondary brain metastases [36]. To this end, our initial goal was to explore the feasibility of developing small molecules capable of targeting EZH2, along with the potential to cross the BBB.

Molecular docking predicted a high potency of our designed compound SG-8 as a putative inhibitor of EZH2 with the estimated K*_i_* = 1.4 nM value being significantly superior compared to those of TDI-6118 (previously known brain-penetrant EZH2i, K*_i_* = 10 nM) and Tazemetostat (K*_i_* = 2.5 nM). Surprisingly, in silico studies suggested an additional mode of interaction for SG-8, as well as for PF-06726304 (a well-known potent EZH2i with a similar chemical structure, K*_i_* = 0.7 nM), with the PRC2 complex. Molecular docking predicted that these compounds could not only bind to EZH2 but also display high calculated affinity for a particular site in the EED subunit. EED protein folds into a canonical β-propeller domain formed by seven β-sheets arranged around a central pore and one α-helix. The central pore at the side near the α-helix (bottom pocket) was suggested as a specific binding site for PF-06726304, TDI-6118, and SG-8. On the other hand, the bottom pocket of EED is the interaction site for the *N*-terminal α-helix of EZH2, which is necessary for the structural integrity and function of the PRC2 complex and for the transmission of activating signals from EED to the EZH2 catalytic domain [27]. Several small molecules targeting the EED bottom pocket have been described, including Astemizole (K*_d_* = 23.01 μM), DC-PRC2in-01 (K*_d_* = 4.56 μM), and Wedelolactone (K*_d_* = 2.82 μM). These compounds disrupt the EED–EZH2 interaction, resulting in: (i) degradation of PRC2 complex members, (ii) reduction in H3K27me3 marks, and ultimately (iii) anti-proliferative effects in hematological malignancies [26,27,60,61,62]. Previous data indicated that both DC-PRC2in-01 [60] and Astemizole [61] downregulated the protein expression levels of EZH2, SUZ12, EED, and H3K27me3, whereas Wedelolactone reduced the levels of EZH2 and EED only [62]. Overall, EED targeting might suggest a novel underlying molecular mechanism of some 3,4-dihydroisoquinoline-1(*2H*)-one derivatives, including PF-06726304, TDI-6118, and SG-8, in addition to their ability to target EZH2.

The BOILED-Egg model predicted a high probability of BBB permeability for SG-8. This in silico prediction was further supported by in vitro results obtained using the Parallel Artificial Membrane Permeability Assay (PAMPA). Specifically, SG-8 demonstrated a passive permeability across the artificial membrane three times greater than the known brain-penetrant compound Promazine, placing it among the most BBB-permeable molecules, such as Imipramine, Terfenadine, and Thiopental [56]. However, this in vitro model has important limitations due to the absence of efflux transporters, among other key features of the physiological BBB. Accordingly, future in vivo studies will be necessary to confirm the BBB permeability of SG-8.

Although SG-8 and PF-06726304 were predicted to exhibit nanomolar binding affinities toward EZH2 and EED, based on in silico docking, both compounds displayed only micromolar cellular EC_50_ values against A375 and Colo-679 melanoma cells. This apparent discrepancy likely reflects the well-recognized differences between predicted or biochemical target affinity and phenotypic cellular potency, which are influenced by various pharmacokinetic and cellular factors, including: (i) intracellular target accessibility, (ii) protein binding, (iii) metabolic stability, and (iv) active efflux. For epigenetic modulators, the magnitude of this gap can be particularly large in solid tumors, which are generally resistant to epigenetic therapies [7,8]. Accordingly, Tazemetostat has also been reported to display nanomolar EZH2 affinity (K*_i_* = 2.5 nM) but only micromolar cellular toxicity in melanoma cells (EC_50_ = 35 μM) [35].

Biological characterization in A375 and Colo-679 cells also revealed that the in silico-predicted ranking of compounds based on K*_i_* values does not fully correspond to their ranking based on cellular EC_50_ values. Although SG-8 has a more favorable predicted EZH2 binding affinity compared to Tazemetostat, it exhibited lower cytotoxic potency, as indicated by its higher EC_50_ values (150 μM for SG-8 vs. 35 μM for Tazemetostat) after 48 h of exposure. Similarly, PF-06726304 (K*_i_* = 0.7 nM) is approximately 3.5 times more potent as an EZH2 inhibitor than Tazemetostat (K*_i_* = 2.5 nM) in biochemical PRC2 assays [10,18], yet it was shown to be 1.5 times less toxic (EC_50_ = 50 μM) in both cell lines. At the same time, the ~3-fold lower cytotoxicity of SG-8 compared to PF-06726304 aligns well with our in silico data, which predicted a higher affinity of PF-06726304 for both EZH2 and EED.

Since 3,4-dihydroisoquinoline-1(2*H*)-one-based compounds (SG-8 and PF-06726304) are chemically distinct from Tazemetostat, they might possess substantial differences in pharmacokinetic properties and cellular target engagement, all of which could account for the unexpectedly higher cytotoxic potency observed for Tazemetostat in our melanoma model. In addition, differences in the mechanism of action between our 3,4-dihydroisoquinoline-1(2*H*)-one derivatives and Tazemetostat are also possible, as discussed below. In contrast, SG-8 and PF-06726304 are closely related structurally, sharing the same 3,4-dihydroisoquinoline-1(2*H*)-one and isoxazole moieties. Their pharmacokinetics and mechanisms of action are therefore expected to be more similar, which is consistent with the good agreement between the relative cytotoxic potencies and the in silico–predicted differences in EZH2 and EED binding affinities.

Immunoblotting analysis partially supported our in silico predictions regarding EED targeting. Both PF-06726304 and, in particular, SG-8 markedly reduced EED protein expression levels in A375 cells—an effect also observed with Tazemetostat, although to a lesser extent. Notably, SG-8 produced the most pronounced reduction in EZH2 protein levels. As previously shown, accelerated degradation of EED and EZH2 mediates the activity of compounds targeting the EED bottom pocket, such as Astemizole, DC-PRC2in-01, and Wedelolactone [60,61,62]. Consequently, these findings suggest that PF-06726304 and SG-8 may interact with EED in the same manner. On the other hand, a rather unexpected H3K27me3 expression pattern was observed following exposure of A375 cells to PF-06726304 and SG-8. Although PF-06726304 is a potent EZH2i capable of reducing H3K27me3 levels in human B-cell non-Hodgkin lymphoma cells [18], such activity was not evident in our study. Specifically, we observed only a slight reduction in H3K27me3 expression levels in PF-06726304-treated cells, whereas SG-8 slightly increased these levels. In contrast, Tazemetostat almost completely abolished H3K27me3 expression.

The protein expression pattern of H3K27me3 was rather unexpected and appears to contradict both the in silico predictions and the remaining Western blot data. As the primary canonical function of PRC2 is the trimethylation of H3K27, inhibition of EZH2 and/or reduced protein levels of key PRC2 components, such as EZH2 and EED, would be expected to result in a corresponding decrease in total H3K27me3 levels. However, as no such decrease was observed, these findings do not provide evidence for inhibition of the canonical EZH2 methyltransferase activity by SG-8 or by PF-06726304 in our in vitro melanoma model under the experimental conditions applied in this study. In this context, our data suggest that, in melanoma cells, the cytotoxic effects of SG-8 and PF-06726304 are likely mediated through mechanisms distinct from the suppression of the canonical EZH2 pathway. This may explain the unexpectedly lower cytotoxic potency of these compounds compared with Tazemetostat.

Similar findings have been reported in Silibinin-treated prostate carcinoma cells (Du145 and PC3) [63]. Specifically, Silibinin caused downregulation of EZH2, SUZ12, and EED expression, an effect accompanied by an upregulation of—rather than a decrease in—H3K27me3 levels. This inverse expression pattern was found to be at least partially associated with reduced phosphorylated Akt (p-Akt) levels and was further linked to depletion of phosphorylated EZH2, a response similar to that observed in our study. In general, p-Akt is the active form of Akt and is responsible for phosphorylating various substrates, including EZH2 at serine 21, thereby decreasing its affinity for histone H3 [59]. Since this phosphorylation diminishes EZH2’s capacity to methylate histone H3, an increased EZH2/p-EZH2 ratio may sustain H3K27me3 levels despite the overall reduction in PRC2 components. In this context, a potential compensatory mechanism for maintaining overall PRC2 activity following EED reduction may involve a shift in EZH2 from its phosphorylated, histone-inactive form to its unphosphorylated, histone-active form.

Recent studies using two known EED-targeting compounds, LG1980 [64] and Nicardipine [65], further support this notion. In chemoresistant prostate cancer cells (C4-2B-TaxR), both LG1980 and Nicardipine were shown to markedly reduce EED protein levels and slightly decrease EZH2 and SUZ12 expression without affecting H3K27me3 marks. Furthermore, both compounds induced cell cycle arrest and apoptotic cell death, potentially through modulation of non-canonical PRC2-related pathways and in association with reduced levels of p-EZH2 (Ser21). In particular, the non-canonical activity of EZH2 refers to functions beyond its well-established methyltransferase activity on histone H3. Depending on its phosphorylation status, EZH2 is able not only to methylate non-histone substrates (in a PRC2-dependent or -independent manner) but also to act as a transcription factor and co-activator of gene expression [66,67]. For instance, phosphorylation at serine 21 has been associated with a functional switch in which EZH2 loses its histone-methylating activity and instead targets STAT3 [68]. In this context, the reduction in p-EZH2 induced by LG1980 and Nicardipine resulted in significant suppression of STAT3 activation, an effect associated with transcriptional inhibition of downstream targets such as Skp, ABCB1 and surviving, ultimately leading to cell death [64,65].

On the other hand, our results revealed that SG-8 induced cell cycle arrest at the G_2_/M phase, accompanied by a parallel decrease in the S phase population. Activation of apoptosis as a potential anti-melanoma mechanism was also observed following treatment of A375 cells with both PF-06726304 and SG-8. Notably, we detected a significant increase in the activity of caspases-8, -9, and -3, thereby indicating activation of both the extrinsic and intrinsic apoptotic pathways. Immunoblotting analysis further demonstrated significant cleavage of poly (ADP-ribose) polymerase (PARP) upon treatment with SG-8 and PF-06726304 at their respective EC_50_ concentrations. PARP plays a crucial role in DNA repair following single-strand breaks. Activated caspase-3 cleaves PARP into two characteristic fragments of 24 kDa and 89 kDa, thereby abrogating DNA repair and rendering the apoptotic process irreversible. Accordingly, the cleaved (Asp214) 89 kDa PARP fragment is widely recognized as a classical biochemical marker of apoptosis. Similarly, PARP cleavage has been reported following treatments with LG1980 and Nicardipine, further supporting the involvement of apoptosis activation as an underlying molecular mechanism of their cytotoxicity [64,65].

**Figure 8:**
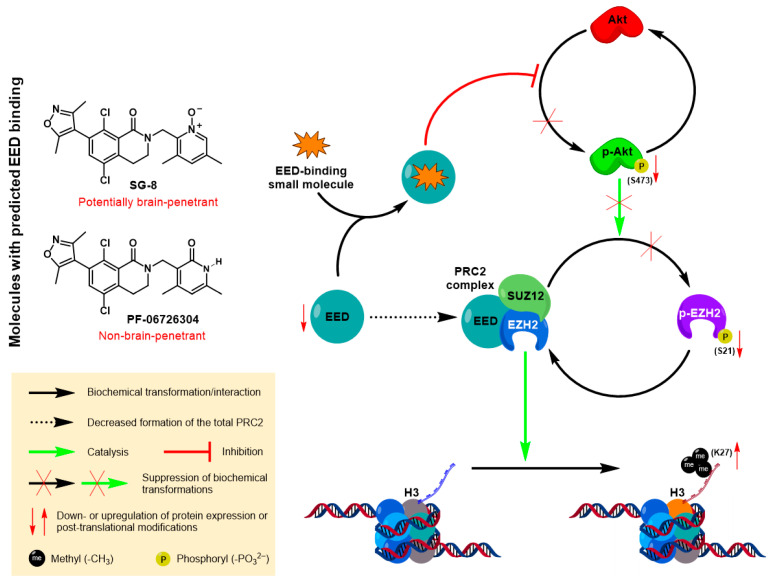
Proposed schematic model summarizing the potential mechanisms of action of SG-8 and PF-06726304 in melanoma cells. Docking calculations suggest that SG-8 and PF-06726304 may interact with the EED subunit of the PRC2 complex. Experimentally, treatment with SG-8 and PF-06726304 is associated with reduced EED protein levels and decreased phosphorylation levels of both Akt (Ser473) and EZH2 (Ser21), consistent with modulation of the Akt–EZH2-related signaling axis. Despite these changes, H3K27me3 levels remain largely unaffected and/or are slightly increased, indicating that canonical PRC2-dependent H3K27 trimethylation is not substantially inhibited under the conditions tested. This observation could be explained by a compensatory shift in the balance from phosphorylated EZH2 (which is inactive towards histone H3) to non-phosphorylated EZH2 (which retains histone H3 methyltransferase activity). All interactions and pathways shown are proposed and are intended as a conceptual framework based on the current data.

Collectively, our data indicate that the anti-melanoma effects of both SG-8 and PF-06726304 may not be related to the inhibition of the canonical EZH2 histone methyltransferase activity since we did not observe any associated and significant decrease in H3K27me3 marks. Instead, an alternative model appears more plausible, in which SG-8 and PF-06726304 primarily modulate the p-Akt/p-EZH2 signaling axis (Figure 8). In line with this hypothesis, immunoblotting analysis showed a pronounced reduction in p-Akt (Ser473) (an upstream regulator of EZH2) as well as p-EZH2 (Ser21) expression levels, whereas total EZH2 levels were only mildly decreased. In parallel, unaffected H3K27me3 marks, despite the strong reduction in EED levels, suggest the potential involvement of compensatory molecular mechanisms. One possible explanation is a shift in the balance between phosphorylated and non-phosphorylated EZH2 ratios, as observed in our study, which may partially preserve canonical EZH2 activity. Notably, co-existence of reduced EED and p-EZH2 levels and preserved H3K27me3 marks, induced by compounds with predicted EED-targeting activity, have been reported previously, suggesting that this pattern may represent a recurring mechanistic feature [63,64,65].

However, it is premature to conclude that SG-8 and PF-06726304 causatively exert their anti-melanoma effects through direct EED targeting and subsequent suppression of the Akt–p-EZH2 (Ser21) axis. The observed alterations in EED, p-EZH2, and p-Akt expression levels may also reflect indirect consequences of SG-8- and PF-06726304-induced cellular stress, growth arrest, and/or apoptosis induction. Therefore, further dedicated mechanistic studies will be required to elucidate the direct molecular targets and signaling pathways responsible for the cellular effects of these compounds in melanoma models.

## 4. Materials and Methods

### 4.1. Molecular Docking

In this study, the file containing the three-dimensional structures of the EZH2, EED, and SUZ12 proteins (PDB ID: *5IJ7*) was retrieved from the RCSB Protein Data Bank (RCSB PDB; https://www.rcsb.org/) [69]. The native ligand, water molecules, and other unwanted molecules and ions were removed from the original PDB file, and the non-target *Anolis carolinensis* protein chain was excised, leaving only the *Homo sapiens* protein chain corresponding to EZH2. To ensure structural completeness, the protein chains were processed with AutoDockTools4 (version 1.5.7, The Scripps Research Institute, La Jolla, CA, USA) [70] prior to docking analysis. This processing included the addition of polar hydrogens, assigning Kollman charges, correcting missing atoms, and assigning bond types. The processed protein files were saved in PDBQT format for subsequent docking analysis.

The structure of the PF-06726304 ligand was extracted from the PubChem database, while SG-8 and TDI-6118 were generated using PubChem Sketcher (web-based tool, National Center for Biotechnology Information, Bethesda, MD, USA; https://pubchem.ncbi.nlm.nih.gov/) [71]. These ligands were imported into UCSF Chimera (version 1.8, University of California, San Francisco, CA, USA) [72] for the addition of hydrogen atoms and Gasteiger charge assignment. Although no specific pH was explicitly set during ligand preparation, both AutoDockTools and Chimera applied default protonation states corresponding to physiological conditions (approximately pH 7.0).

The EZH2 binding site was determined based on the SET domain, as PF-06726304 and other related EZH2 inhibitors have been reported in the literature to exhibit a strong affinity for this region (PDB ID: *6B3W*, *4W2R*, *5IJ7*, *5WFD*, *5WFC*, *5LS6*). A grid box with dimensions of 64, 106, and 58 grid points along the x-, y-, and z-axes, respectively, with a spacing of 0.460 Å, was centered at (12.921, −4.502, 285.938). Binding sites for auxiliary targets EED and SUZ12 were defined using grid boxes of 40 × 40 × 40 grid points (spacing: 0.50833 Å; center: 41.861, 25.281, 192.997) and 82 × 108 × 112 grid points (spacing: 0.502777 Å; center: 59.56, 7.535, 231.38). Since the binding sites of EED and SUZ12 are not well defined in the literature, they were identified through systematic grid box mapping.

Docking calculations were performed using AutoDock4 (version 4.2, The Scripps Research Institute, La Jolla, CA, USA) [70] with the Lamarckian Genetic Algorithm (LGA), a widely used method for conformational searching and binding pose prediction. The parameters included 150 genetic algorithm (GA) runs, a population size of 150, 27,000 generations, and 2,500,000 energy evaluations. The poses were ranked according to predicted binding free energies. Docked complexes were analyzed for hydrogen bonding, π–π stacking, and hydrophobic interactions in BIOVIA Discovery Studio Visualizer (version 2024, Dassault Systèmes BIOVIA, San Diego, CA, USA) [73]. High-affinity poses were selected based on docking scores and the quality of key interactions.

### 4.2. Physicochemical and Pharmacokinetic Analyses

All physicochemical data and the BOILED-Egg diagram were obtained computationally using the SwissADME web service (http://www.swissadme.ch/, Swiss Institute of Bioinformatics, Lausanne, Switzerland) [49]. WlogP and topological polar surface area (TPSA, Å^2^) were utilized to construct the BOILED-Egg plot, which served as a predictive model for gastrointestinal absorption and blood–brain barrier permeability [48]. The WlogP value was calculated by the atomistic method of Wildman and Crippen [53], while TPSA was determined as the sum of fragment-based contributions [54].

### 4.3. Materials and Equipment for Organic Synthesis

Chemical reagents and solvents were purchased from Fluorochem Ltd. (Glossop, UK), Sigma-Aldrich (Merck Group; Saint Louis, MO, USA), Angene International Ltd. (London, UK), and Honeywell (Charlotte, NC, USA) and used without further purification. For thin-layer chromatography (TLC), silica plates (60Å, F254, aluminum support; Fluorochem Ltd., Glossop, UK) were used. A UV lamp or an iodine chamber was employed for the visualization of the TLC plates. A silica gel with 60 Å pore size and 35–70 μm particle size (Fluorochem Ltd.; Glossop, UK) was utilized for the column chromatography. ^1^H and ^13^C NMR spectra were recorded on a spectrometer Bruker UltraShieldPlus 500 MHz (Bruker BioSpin GmbH, Rheinstetten, Germany). The attached proton test (APT) was performed to assign CH_3_, CH_2_, CH, and quaternary carbon (C) signals in ^13^C NMR spectra. Tetramethylsilane (TMS) was used as the internal standard for chemical shifts (ppm). The deuterated solvents CDCl_3_ and DMSO-*d_6_*, containing TMS, were purchased from Eurisotop (Saint-Aubin, France). The purities of all products were assessed by ^1^H NMR and TLC analyses and was found to be >95%.

High-resolution mass spectrometry (HRMS) experiments were conducted using an Agilent REVIDENT Quadrupole Time-Of-Flight (Q-TOF) mass spectrometer, coupled to an Agilent 1290 Infinity II UPLC system via a Dual AJS electrospray ionization (ESI) source (Agilent Technologies, Santa Clara, CA, USA). The Agilent 1290 UPLC system included a binary pump, an online vacuum degasser, an autosampler, and a diode array detector. Mass spectrometric analysis was carried out in positive electrospray ionization (ESI) mode. The autosampler temperature was maintained at 4 °C, while an isocratic elution with 100% acetonitrile was performed for 2 min. The flow rate was set at 250 μL/min, and the injection volume was 5 μL. The resolving power of the Q-TOF analyzer was set to >10,000 (FWHM, full width at half maximum), and spectra were acquired over a mass range of *m*/*z* 400–500. Nitrogen gas (225 °C, 6 L/min, 40 psi) was used for nebulization and drying in the ionization source. The sheath gas was maintained at 350 °C with a flow rate of 10 L/min, the fragmentor was set to 145 V, and the skimmer to 65 V. The capillary, nozzle, and octapole 1RF voltages were set 3000 V, 0 V, and 450 V, respectively. Data were acquired under continuous reference mass correction at *m*/*z* 121.0509 and 922.0890 in positive ion mode.

### 4.4. Synthetic Procedures

**Figure 9:**
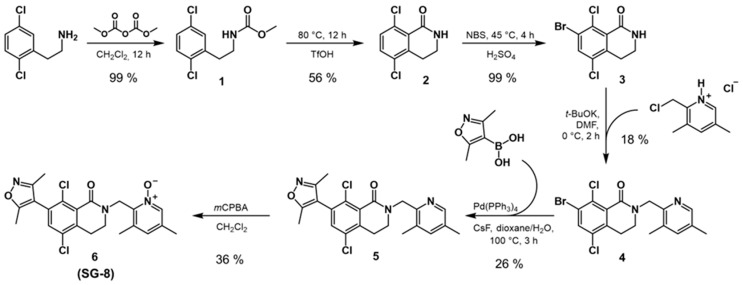
General synthetic route that has been followed for the preparation of SG-8 (**6**). The numbering of compounds **1**–**6** is retained throughout the text for referencing the synthetic procedures [18,43].

A general synthetic route was followed for the preparation of SG-8 (Figure 9), as described below.

#### 4.4.1. Synthesis of Methyl (2,5-Dichlorophenethyl)carbamate (**1**)

A solution of dimethyl dicarbonate (16.1 g, 0.12 mol, 1.2 eq) in dichloromethane (50 mL) was added dropwise to a cooled (0 °C) solution of 2-(2,5-dichlorophenethyl)ethanamine (19.0 g, 0.1 mol, 1 eq) in dichloromethane (50 mL). The reaction mixture was then stirred overnight at room temperature. Upon completion of the reaction, the solvents were removed under reduced pressure, and the resulting residue was quenched with 60 mL of water–methanol mixture (1:5, *v*/*v*) for 12 h. After final evaporation, pure product **1** was obtained as a yellow solid (24.5 g, 0.099 mol, 99%).

^1^H NMR (500 MHz, CDCl_3_) *δ* (ppm): 2.93 (t, *J* = 6.9 Hz, 2H, CH_2_), 3.44 (q, *J* = 6.6 Hz, 2H, CH_2_), 3.67 (s, 3H, CH_3_), 4.87 (brs, 1H, NH), 7.15 (dd, *J* = 2.5, 8.5 Hz, 1H, Ar), 7.22 (d, *J* = 1.9 Hz, 1H, Ar), 7.28 (d, *J* = 8.4 Hz, 1H, Ar);

^13^C NMR (126 MHz, CDCl_3_) (ppm): 33.9 (CH_2_), 40.4 (CH_2_), 52.1 (CH_3_), 128.1 (CH, Ar), 130.7 (CH, Ar), 130.8 (CH, Ar), 132.4 (Ar), 132.6 (Ar), 138.3 (Ar), 157.0 (CO).

#### 4.4.2. Synthesis of 5,8-Dichloro-3,4-dihydroisoquinolin-1(*2H*)-one (**2**)

Compound **1** (20.9 g, 84 mmol) was slowly added in small portions to cold (0 °C) pure triflic acid (270 mL). The resulting mixture was stirred for 45 min until a homogeneous dark orange solution was formed. This solution was heated at 80 °C for 12 h and finally poured into 125 g of crushed ice, leading to the formation of a white precipitate. The mixture was then filtered, and the filtrate was extracted with dichloromethane (3 × 385 mL). The lower organic layer was combined with the precipitate from the previous filtration, and the resulting solution was washed with 1 M NaOH (155 mL), with brine (80 mL), and then dried over MgSO_4_. Evaporation under reduced pressure afforded a dark crude powder (15.6 g). The residue was stirred in a 120 mL mixture of petroleum ether and ethyl acetate (5:1) for 30 min and then filtered. Compound **2** (10.2 g, 47 mmol, 56%) was obtained as a white powder after drying.

^1^H NMR (500 MHz, CDCl_3_) *δ* (ppm): 2.97 (t, *J* = 6.3 Hz, 2H, CH_2_), 3.33 (td, *J* = 3.8, 6.3 Hz, 2H, CH_2_), 7.43 (d, *J* = 8.6 Hz, 1H, Ar), 7.59 (d, *J* = 8.6 Hz, 1H, Ar), 8.28 (brt, *J* = 3.8 Hz, 1H, NH);

^13^C NMR (126 MHz, CDCl_3_) *δ* (ppm): 27.6 (CH_2_), 38.4 (CH_2_), 129.1 (Ar), 130.8 (Ar), 131.4 (CH, Ar), 132.6 (Ar), 132.7 (CH, Ar), 140.7 (Ar), 161.9 (CO).

#### 4.4.3. Synthesis of 7-Bromo-5,8-dichloro-3,4-dihydroisoquinolin-1(*2H*)-one (**3**)

Compound **2** (8.8 g, 41 mmol, 1 eq) was suspended in H_2_SO_4_ (98%, 425 mL), and then *N*-bromosuccinimide (8.0 g, 45 mmol, 1.1 eq) was added in small portions. For the next 4 h, the temperature was elevated up to 45 °C. Upon completion of the reaction, the mixture was poured into crushed ice (200 g). The resulting precipitate was filtered, washed thoroughly with water (5 × 80 mL), and dried under vacuum at 90 °C for 3 h to yield Compound **3** (12.0 g, 40.5 mmol, 99%) as a white powder.

^1^H NMR (500 MHz, DMSO-*d*_6_) *δ* (ppm): 2.93 (t, *J* = 6.3 Hz, 2H, CH_2_), 3.33 (td, *J* = 3.9, 6.2 Hz, 2H, CH_2_), 8.09 (s, 1H, Ar), 8.40 (brt, *J* = 3.4 Hz, 1H, NH);

^13^C NMR (126 MHz, DMSO-*d*_6_) *δ* (ppm): 27.6 (CH_2_), 38.3 (CH_2_), 123.8 (Ar), 131.1 (Ar), 131.4 (Ar), 132.5 (Ar), 135.6 (CH, Ar), 140.2 (Ar), 161.4 (CO).

#### 4.4.4. Synthesis of 7-Bromo-5,8-dichloro-2-((3,5-dimethylpyridin-2-yl)methyl)-3,4-dihydroisoquinolin-1(*2H*)-one (**4**)

A portion of Compound **3** (2.0 g, 6.78 mmol, 1 eq) was mixed with 15 mL of anhydrous dimethylformamide at 0 °C, and 16.4 mL of 1 M potassium *tert*-butoxide in tetrahydrofuran (16.4 mmol) was added dropwise. Then, the mixture was stirred for 2 h (0 °C). Subsequently, a solution of 2-(chloromethyl)-3,5-dimethylpyridine hydrochloride (1.43 g, 7.46 mmol, 1.1 eq) in 15 mL of anhydrous dimethylformamide was added dropwise. After stirring at 0 °C for 30 min, the resulting solution was mixed with ethyl acetate (200 mL) and water (200 mL). The upper layer was separated, washed with water (1 × 200 mL) and brine (1 × 200 mL), dried over MgSO_4_, and evaporated to dryness. The resulting crude product was mixed with 250 mL of ethyl acetate and 250 mL of 1 M HCl. A white crystalline precipitate of pure hydrochloride salt of Compound **4** was formed. It was filtered, washed with a small amount of cold ethyl acetate, and dried on the filter. The acidic aqueous layer was neutralized with 5 M NaOH (pH 11) and extracted with 100 mL of dichloromethane. The organic layer was washed with water (100 mL) and brine (100 mL), dried over MgSO_4_, and concentrated under reduced pressure. After mixing this crude compound with 10 mL of ethyl acetate and 10 mL of 1 M HCl, an additional amount of hydrochloride salt of Compound **4** was crystallized at 4 °C. The total amount of the hydrochloride salt of Compound **4** was 0.67 g (1.5 mmol, 22%).

^1^H NMR (500 MHz, DMSO-*d*_6_) *δ* (ppm): 2.43 (s, 3H, CH_3_), 2.44 (s, 3H, CH_3_), 3.12 (t, *J* = 6.3 Hz, 2H, CH_2_), 3.72 (t, *J* = 6.3 Hz, 2H, CH_2_), 5.03 (s, 2H, CH_2_), 8.17 (s, 1H, Ar), 8.20 (brs, 1H, Py), 8.51 (brs, 1H, Py);

^13^C NMR (126 MHz, DMSO-*d*_6_) *δ* (ppm): 17.6 (CH_3_), 17.7 (CH_3_), 26.8 (CH_2_), 45.9 (CH_2_), 48.2 (CH_2_), 123.9 (Ar), 130.4 (Ar), 131.3 (Ar), 132.9 (Ar), 135.6 (Py), 135.9 (Py), 136.0 (CH, Ar), 139.6 (CH, Py), 140.1 (Ar), 146.9 (CH, Py), 148.4 (Py), 161.4 (CO).

The hydrochloride salt of Compound **4** was converted into the free base by the following procedure: 10 mL of 1 M NaOH solution was added to 0.60 g (1.33 mmol) of hydrochloride of Compound **4**. The resulting mixture was extracted with dichloromethane (3 × 10 mL). The organic phase was washed with brine (1 × 10 mL) and evaporated with a vacuum. The free base of Compound **4** was obtained as a white powder (0.45 g, 1.09 mmol, 82%).

^1^H NMR (500 MHz, CDCl_3_) *δ* (ppm): 2.29 (s, 3H, CH_3_), 2.35 (s, 3H, CH_3_), 2.99 (t, *J* = 6.2 Hz, 2H, CH_2_), 3.58 (t, *J* = 6.2 Hz, 2H, CH_2_), 4.89 (s, 2H, CH_2_), 7.28 (brs, 1H, Py), 7.76 (s, 1H, Ar), 8.20 (brs, 1H, Py);

^13^C NMR (126 MHz, CDCl_3_) *δ* (ppm): 18.1 (CH_3_), 18.4 (CH_3_), 26.9 (CH_2_), 44.7 (CH_2_), 49.9 (CH_2_), 124.4 (Ar), 130.8 (Ar), 131.0 (Ar), 131.7 (Ar), 132.4 (Py), 133.9 (Py), 135.5 (CH, Ar), 139.0 (Ar), 139.2 (CH, Py), 147.1 (CH, Py), 151.5 (Py), 160.9 (CO).

#### 4.4.5. Synthesis of 5,8-Dichloro-2-((3,5-dimethylpyridin-2-yl)methyl)-7-(3,5-dimethyl isoxazol-4-yl)-3,4-dihydroisoquinolin-1(*2H*)-one (**5**)

To a solution of Compound **4** (0.30 g, 0.724 mmol, 1 eq), (3,5-dimethylisoxazol-4-yl)boronic acid (0.143 g, 1.015 mmol, 1.4 eq) and tetrakis(triphenylphosphine)palladium(0) (0.121 g, 0.10 mmol, 0.14 eq) in dioxane (6 mL) and water (1 mL), cesium fluoride (0.556 g, 3.66 mmol, 5 eq) was added at 25 °C. The mixture was degassed with a stream of nitrogen and stirred at 100 °C for 3 h. After cooling to room temperature, the mixture was poured into water (30 mL) and extracted with ethyl acetate (4 × 20 mL). The combined organic layers were washed with saturated brine (2 × 50 mL), dried over anhydrous MgSO_4_, filtered, and the filtrate was concentrated under reduced pressure. The residue was purified by column chromatography (*n*-hexane/ethyl acetate/triethylamine = 50:50:3, *R_f_* = 0.41) to afford Compound **5** (0.080 g, 0.186 mmol, 26%) as a pale-yellow solid.

^1^H NMR (500 MHz, CDCl_3_) *δ* (ppm): 2.15 (s, 3H, CH_3_), 2.28 (s, 3H, CH_3_), 2.29 (s, 3H, CH_3_), 2.37 (s, 3H, CH_3_), 3.09 (t, *J* = 6.3 Hz, 2H, CH_2_), 3.65 (t, *J* = 6.3 Hz, 2H, CH_2_), 4.90 (d, *J* = 3.1 Hz, 2H, CH_2_), 7.29 (brs, 1H, Py), 7.29 (s, 1H, Ar), 8.21 (brs, 1H, Py);

^13^C NMR (126 MHz, CDCl_3_) *δ* ppm 10.6 (CH_3_), 11.7 (CH_3_), 17.9 (CH_3_-Py), 18.3 (CH_3_-Py), 26.9 (CH_2_), 44.6 (CH_2_), 49.7 (CH_2_), 114.1 (Ar), 130.1 (Ar), 130.5 (Ar), 131.4 (Ar), 131.6 (Ar), 132.3 (Ar), 134.0 (CH, Ar), 134.2 (Ar), 139.0 (CH, Py), 140.0 (Ar), 147.0 (CH, Py), 151.5 (Ar), 159.2 (Ar), 161.2 (CO), 166.6 (Ar).

HRMS (ESI^+^) *m*/*z*: calculated for C_22_H_21_Cl_2_N_3_O_2_, 430.1084 [M + H]^+^, 452.0903 [M + Na]^+^; observed 430.1072 [M + H]^+^, 452.0890 [M + Na]^+^.

#### 4.4.6. Synthesis of 5,8-Dichloro-2-[(3,5-dimethyl-1-oxo-1λ^5^-pyridin-2-yl)methyl]-7-(3,5-dimethyl isoxazol-4-yl)-3,4-dihydroisoquinolin-1(*2H*)-one (**6**, SG-8)

To a solution of Compound **5** (40 mg, 0.0930 mmol, 1 eq) in dichloromethane (1 mL), 3-chloroperoxybenzoic acid (*m*CPBA, 70%), (25 mg, 0.101 mmol, 1.09 eq) was added. After 24 h, the solution was quenched with aqueous solution of sodium metabisulfite (5%, 1 mL, 1 h). Further, the mixture was extracted with an additional 9 mL of dichloromethane and 10 mL of NaOH solution in water (0.1 M). The organic phase was washed with 10 mL of water and 10 mL of brine, dried over MgSO_4_, and concentrated under reduced pressure. The crude compound was purified by column chromatography (diethyl ether/methanol = 90:10, *R_f_* = 0.21) to afford Compound **6** (15 mg, 0.0336 mmol, 36%) as a colorless solid.

^1^H NMR (500 MHz, CDCl_3_) *δ* ppm 2.13 (s, 3H, CH_3_), 2.25 (s, 3H, CH_3_), 2.26 (s, 3H, CH_3_), 2.62 (s, 3H, CH_3_), 3.13 (t, *J* = 6.2 Hz, 2H, CH_2_), 3.98 (t, *J* = 6.2 Hz, 2H, CH_2_), 4.92 (s, 2H, CH_2_), 6.96 (s, 1H, Py), 7.28 (s, 1H, Ar), 7.99 (s, 1H, Py);

^13^C NMR (126 MHz, CDCl_3_) *δ* ppm 10.6 (CH_3_), 11.6 (CH_3_), 18.0 (CH_3_), 19.3 (CH_3_), 27.7 (CH_2_), 44.6 (CH_2_), 47.5 (CH_2_), 114.0 (Ar), 129.8 (CH, Py), 130.6 (Ar), 131.2 (Ar), 134.0 (Ar), 134.1 (CH, Ar), 134.7 (Ar), 137.1 (Ar), 137.2 (CH, Py), 140.5 (Ar), 142.9 (Ar), 159.2 (Ar), 161.8 (CO), 166.6 (Ar).

HRMS (ESI^+^) *m*/*z*: calculated for C_22_H_21_Cl_2_N_3_O_3_, 446.1033 [M + H]^+^, 468.0852 [M + Na]^+^; observed 446.1023 [M + H]^+^, 468.0841 [M + Na]^+^.

### 4.5. Materials for Biological Characterization

Dulbecco’s Modified Eagle’s Medium (DMEM) was purchased from Capricorn Scientific GmbH (Ebsdorfergrund, Germany). RPMI-1640 (Roswell Park Memorial Institute) medium was purchased from Biosera (East Sussex, UK). Dulbecco’s phosphate-buffered saline (PBS), *L*-Glutamine 100X, Penicillin-Streptomycin solution, and trypsin-EDTA were purchased from Biosera (East Sussex, UK). Fetal bovine serum (FBS) was purchased from PAN-Biotech (Aidenbach, Germany). Dimethyl sulfoxide (DMSO) was purchased from PAN-Biotech (Aidenbach, Germany). Tazemetostat was obtained from MedChemExpress LLC (Monmouth Junction, NJ, USA). PF-06726304 (98%, CAS: 1616287-82-1) was produced by BLD Pharmatech GmbH (Reinbek, Germany). SG-8 was synthesized according to synthetic procedures in Section 4.4. Pure compounds PF-06726304 and SG-8 were dissolved in DMSO. Resazurin sodium salt was purchased from Fluorochem Ltd. (Glossop, UK). Amersham™ Hybond^®^ polyvinylidene fluoride (PVDF) blotting membranes with 0.2 μm pore size (GE Healthcare Life Sciences; Düsseldorf, Germany) were used for Western blot.

### 4.6. In Vitro Determination of Blood–Brain Barrier Membrane Permeability

The Parallel Artificial Membrane Permeability Assay–BBB Kit (PMBBB-096; BioAssay Systems, Hayward, CA, USA) was employed to determine the passive permeability rate of compounds across the blood–brain barrier (BBB), following the manufacturer’s protocol. In brief, stock solutions of the compounds (10 mM in DMSO) were prepared and subsequently diluted in PBS to a final concentration of 500 μM for testing. High-permeability (Promazine) and low-permeability (Diclofenac) controls were provided by the manufacturer. A volume of 200 μL of each test solution was added to the donor wells, while the acceptor wells were filled with 300 μL of PBS. A hydrophobic PVDF membrane (0.45 µm pore size), incorporating the manufacturer’s lipid composition solubilized in dodecane, served as the artificial BBB. The assay was conducted for 18 h at 37 °C. All experiments were performed in triplicates.

Final compound concentrations in the acceptor wells (C_A_) were determined spectrophotometrically. For this purpose, 200 μL of each acceptor solution was diluted with PBS to a final volume of 1 mL and transferred to a disposable UVette UV/Vis cuvette (Eppendorf SE, Hamburg, Germany) for measurements. In parallel, 40 μM PBS solutions of each compound were prepared and measured as equilibrium standards. Absorbance values (optical densities, ODs) were recorded at 240, 250, and 270 nm using Thermo Scientific Evolution 260 Bio UV-Visible Spectrophotometer (Thermo Fisher Scientific Inc., Waltham, MA, USA). The wavelength yielding the highest OD was selected for each compound to calculate its C_A_: 240 nm for PF-06726304 and SG-8; 250 nm for Promazine and Tazemetostat; 270 nm for Diclofenac. C_A_ values were calculated by dividing the OD of each sample by that of the corresponding equilibrium standard and multiplying by 200 μM. These values were used to compute effective permeability rates (P_e_).

### 4.7. Cell Lines and Culture Conditions

Melanoma A375 cells were purchased from the American Type Culture Collection (ATCC; Manassas, VA, USA) and cultured in DMEM supplemented with *L*-glutamine (2 mM), 10% fetal bovine serum (FBS), 1% Pen/Strep. In addition, Colo-679 cells were purchased from Deutche Sammlung von Mikroorganismen und Zellkulturen (DSMZ; Braunschweig, Germany) and cultured in RPMI-1640 supplemented with *L*-glutamine (2 mM), 10% fetal bovine serum (FBS), 1% penicillin–streptomycin. Cells were maintained in a humidified incubator, at 5% CO_2_, and 37 °C.

### 4.8. Determination of Cell Viability

Cell viability was determined by the Alamar Blue assay. Specifically, cell seeding densities were as follows: (i) A375 cells: 8000, 4000, and 2000 cells/well, (ii) Colo-679 cells: 10,000, 5000, and 2500 cells/well, for 24, 48, and 72 h exposure periods, respectively. Cells were incubated overnight (5% CO_2_, 37 °C in a humidified incubator) and the next day were treated with the following exposure conditions: (i) control (untreated cells), (ii) DMSO, 0.15%, (iii) DMSO 10% (positive control), (iv) Tazemetostat, 35 μM, (v) SG-8 and PF-06726304 in the following concentrations: 1, 10, 25, 50, 100 and 150 μM. Each experimental condition was performed in pentaplicate, while experiments were repeated three independent times. At the end of treatments, cells were further incubated for 4 h with 0.01% of resazurin dye, at 37 °C and 5% CO_2._ Finally, absorbance was measured at 570 nm and 600 nm (reference wavelength) by the Epoch 2 microplate spectrophotometer (BioTek Instruments, Inc., Winooski, VT, USA). Cell viability levels were expressed as percentages (%) of 0.15% DMSO-treated cells, while corresponding EC_50_ values were calculated by the GraphPad Prism software (version 8.0.0, GraphPad Software, San Diego, CA, USA).

### 4.9. Western Immunoblotting

Following exposure of A375 cells with Tazemetostat (35 μM), PF-06726304 (50 μM), and SG-8 (150 μM) for 48 h, cells were trypsinized, washed with PBS, and lysed with Radio-Immunoprecipitation Assay (RIPA) buffer (150 mM sodium chloride, 50 mM Tris-HCl pH 8.0, 1% NP-40, 0.5% sodium deoxycholate, 0.1% SDS) (supplemented with protease and phosphatase inhibitors. Lysis was performed at 4 °C for 30 min, while lysates were vortexed every 10 min, before being centrifuged (4 °C, 14,000× *g*, 15 min) to obtain whole cell extracts (i.e., supernatants). Protein concentrations of the samples were determined by the Pierce™ BCA Protein Assay Kit (Thermo Fisher Scientific; Rockford, IL, USA). Next, whole cell extracts (60 μg) were prepared and subjected to electrophoresis on FastGene^®^ PAGE precast gels (gradient: 4–20%), using a FastGene^®^ PAGE Protein system (Nippon Genetics, Düren, Germany). Separated proteins were transferred to PVDF membranes using the semi-dry Trans-Blot^®^ Turbo™ transfer system (Bio-Rad Laboratories, Inc.; Hercules, CA, USA), while non-specific sites were blocked with either 5% non-fat dry milk or 5% bovine serum albumin (BSA) in Tris-buffered saline with Tween^®^-20 (TBST) buffer (150 mM NaCl, 100 mM Tris pH 7.5, and 0.1% (*v*/*v*) Tween-20) for 2 h at room temperature (RT). Next, membranes were incubated overnight at 4 °C with specific primary antibodies (Table S2).

The next day, membranes were washed with TBST (3 × 10 min), followed by the incubation with the appropriate secondary antibody for 1h at RT. Finally, membranes were washed with TBST (3 × 10 min) and developed using the FUSION solo X imaging system (VilberLourmat; Collégien, France). Membranes were stripped with a stripping buffer (62.5 mM Tris-HCl pH 6.7, 2% SDS, 6 µL/mL 2-mercaptoethanol) and re-probed with an appropriate antibody. To ensure equal protein loading, each membrane was stripped and re-probed with anti-β-actin antibody, while band densitometry was performed with the Image J software (version 1.44n, National Institutes of Health, Bethesda, MD, USA).

### 4.10. Flow Cytometry-Based Cell Cycle Analysis

After treating A375 cells with Tazemetostat (35 μM), PF-06726304 (50 μM) and SG-8 (150 μM) for 48 h, the cells were trypsinized, washed with PBS and fixed in 70% ethanol at 5 °C for at least 24 h. Fixed cells were then washed three times with PBS and permeabilized with 0.3% Triton X-100 for 10 min at room temperature. Following permeabilization, the cells were centrifuged at 500× *g* for 5 min and the supernatant was discarded. The pellet was incubated with RNase (0.03 mg/mL) for 1 h, followed by staining with propidium iodide (0.1 mg/mL) for 15 min in the dark. Samples were analyzed in 96-well plates using a NovoCyte Advanteon Flow Cytometer (Agilent Technologies, Inc.; Santa Clara, CA, USA), while 50,000 events per condition were recorded. Data analysis was performed using NovoExpress software, version 2.2.0 (Agilent Technologies, Inc.; Santa Clara, CA, USA). Doublets and cell aggregates were excluded by gating on forward scatter area (FSC-A) versus forward scatter height (FSC-H). DNA content histograms were analyzed using the Watson Pragmatic model implemented in the NovoExpress cell cycle analysis module. The G_2_/M peak was constrained to twice the G_0_/G_1_ DNA content, and identical coefficients of variation (CVs) were applied to G_0_/G_1_ and G_2_/M peaks. Debris was modeled automatically by the software. The percentages of cells in G_0_/G_1_, S, and G_2_/M phases were calculated accordingly. Each condition was tested in triplicate.

### 4.11. Flow Cytometry-Based Determination of Apoptosis Activation

A375 cells were exposed to Tazemetostat (35 μM), PF-06726304 (50 μM) or SG-8 (150 μM) for 48 h. Following treatments, cells were collected by trypsinization, washed three times with PBS and resuspended in 1X binding buffer at a density of 1 × 10^6^ cells/mL. Subsequently, 5 μL of Annexin V was added, and the cells were incubated for 20 min in the dark at RT. An additional 2 mL of 1X binding buffer was added, and the cells were centrifuged at 2000 rpm for 5 min. The supernatant was discarded and the cell pellet was taken up in 200 μL of binding buffer. Propidium iodide (5 μL) was then added to the cell suspension and the cells were incubated for an additional 15 min in the dark at RT. Samples were analyzed in 96-well plates using a NovoCyte Advanteon Flow Cytometer (Agilent Technologies, Inc.; Santa Clara, CA, USA), while 50,000 events per condition were recorded. Data analysis was performed using NovoExpress software (version 2.2.0, Agilent Technologies, Inc.; Santa Clara, CA, USA).

### 4.12. Determination of Caspases-3, -8, and -9 Enzymatic Activity Levels

A375 cells were seeded in black 96-well plates at a density of 4000 cells/well. After a 16 h incubation at 37 °C with 5% CO_2_, cells were treated with Tazemetostat (35 μM), PF-06726304 (50 μM) and SG-8 (150 μM) for 48 h. Apoptosis was assessed using the caspase-3/7, caspase-8, and caspase-9 Multiplex Activity Assay Kit (Fluorometric) (ab219915; Abcam Limited, Cambridge, UK). Caspase activity levels were quantified using an Agilent BioTek Synergy H1 multimode fluorescence microplate reader (Agilent Technologies, Inc.; Santa Clara, CA, USA) with the following excitation/emission wavelengths: (i) 535/620 nm for caspase-3, (ii) 490/525 nm for caspase-8, and (iii) 370/450 nm for caspase-9. Each condition was tested in triplicate. Data analysis and interpretation were performed according to the manufacturer’s instructions. Relative fluorescence units (RFU) were transformed into fold of caspase activation relative to control.

### 4.13. Statistical Analyses

Data were expressed as mean values ± standard error of the mean (SEM) and comparisons were performed between untreated (control) and treated samples. Statistical analyses were performed by one-way analysis of variance (one-way ANOVA) with Tukey’s post hoc test for multiple comparisons, using GraphPad Prism software, version 8.0.0 (San Diego, CA, USA). Values of *p* < 0.05, *p* < 0.01, *p* < 0.001, and *p* < 0.0001 were considered statistically significant compared with untreated (control) samples.

## Figures and Tables

**Table 2:** Half-maximal effective concentrations (EC_50_) of PF-06726304 and SG-8 against primary (A375) and metastatic (Colo-679) human malignant melanoma cells. Data are presented as means ± SEM and are representative of three independent experiments.

	EC_50_ (μM)			
	A375		Colo-679	
Time (h)	PF-06726304	SG-8	PF-06726304	SG-8
24	94.0 ± 6.0	>150.0	84.0 ± 4.0	>150.0
48	52.0 ± 5.0	150.0 ± 6.0	46.0 ± 5.0	148.0 ± 9.0
72	42.0 ± 3.0	119.0 ± 12.0	29.0 ± 4.0	122.0 ± 15.0

## Data Availability

All data are available within this manuscript including the Supplementary Materials.

## Supplementary Materials

The following supporting information can be downloaded at: https://www.mdpi.com/article/10.3390/ijms27062647/s1.

## Abbreviations

The following abbreviations are used in this manuscript:

ABCB1	ATP-binding cassette sub-family B member 1 (ABCB1); P-glycoprotein
ABCG2	ATP-binding cassette super-family G member 2
ADP	Adenosine diphosphate
Akt	Protein kinase B
Ar	Aryl
BBB	Blood–brain barrier
BCRP	Breast Cancer Resistance Protein; ABCG2
BCA	Bicinchoninic acid
BRAF	Proto-oncogene B-Raf
brs	Broad singlet (NMR)
brt	Broad triplet (NMR)
BSA	Bovine serum albumin
CAS	Chemical Abstracts Service
Caspase	Cysteine-aspartic protease
CTLA-4	Cytotoxic T-lymphocyte associated protein 4
d	Doublet (NMR)
dd	Doublet of doublets (NMR)
DMEM	Dulbecco’s Modified Eagle Medium
DMF	Dimethylformamide
DMSO	Dimethyl sulfoxide
DMSO-*d*_6_	Deuterated DMSO
DNA	Deoxyribonucleic acid
DNMTi	DNA methyltransferase inhibitor
EC_50_	Half maximal effective concentration
EDTA	Ethylenediaminetetraacetic acid
EED	Embryonic Ectoderm Development
ERK	Extracellular signal-regulated kinase
EZH1	Enhancer of Zeste Homolog 1
EZH2	Enhancer of Zeste Homolog 2
EZH2i	EZH2 inhibitor
ESI	Electrospray ionization
FBS	Fetal bovine serum
FDA	The United States Food and Drug Administration
FWHM	Full width at half maximum
GA	Genetic algorithm
GI	Gastrointestinal
H3	Histone 3
H3K27me3	Trimethylated lysine 27 in H3
HDACi	Histone deacetylase inhibitor
HMTi	Histone methyltransferase inhibitor
HRMS	High-resolution mass spectrometry
IC_50_	Half maximal inhibitory concentration
*J*	*J*-coupling; spin-spin coupling (NMR)
LGA	Lamarckian genetic algorithm
MAPK	Mitogen-activated protein kinase
*m*CPBA	*meta*-Chloroperoxybenzoic acid
MDR1	Multidrug resistance protein 1; P-glycoprotein
MEK	Mitogen-activated protein kinase kinase; MAPKK
*m*/*z*	Mass-to-charge ratio
NBS	*N*-Bromosuccinimide
NMR	Nuclear Magnetic Resonance
OD	Optical density
PAGE	Polyacrylamide gel electrophoresis
p-Akt	Phosphorylated Akt
PAMPA	Parallel artificial membrane permeability assay
PARP	Poly (ADP-ribose) polymerase
PBS	Phosphate-buffered saline
PD-1	Programmed cell death protein 1
PDB	The Protein Data Bank
p-EZH2	Phosphorylated EZH2
P-gp	P-glycoprotein
PI	Propidium iodide
PI3K	Phosphoinositide 3-kinase
PPI	Protein–protein interaction
ppm	Parts per million
PRC2	Polycomb Repressive Complex 2
PS	Phosphatidylserine
PVDF	Polyvinylidene fluoride
Py	Pyridine/pyridyl
q	Quartet (NMR)
Q-TOF	Quadrupole Time-of-Flight
RFU	Relative fluorescence units
RIPA	Radio-Immunoprecipitation Assay
RNA	Ribonucleic acid
RPMI	Roswell Park Memorial Institute
RT	Room temperature
s	Singlet (NMR)
SAM	*S*-Adenosyl methionine
SEM	Standard error of the mean
SET	Su(var)3-9, Enhancer-of-zeste, and Trithorax domain
SDS	Sodium dodecyl sulfate
Skp	Seventeen kilodalton protein
STAT3	Signal transducer and activator of transcription 3
SUZ12	Suppressor of Zeste 12
t	Triplet (NMR)
TaxR	Docetaxel resistant
TBST	Tris-Buffered Saline with Tween 20
td	Triplet of doublets (NMR)
Tf	Triflic/Triflate; Trifluoromethanesulfonic/Trifluoromethanesulfonate
TLC	Thin layer chromatography
TMS	Tetramethylsilane
TNF	Tumor Necrosis Factor
TPSA	Topological polar surface area
TRAIL	TNF-related apoptosis-inducing ligand
