# Supplementary material for: Biological Evaluation of a Novel Compound with Predicted EZH2 and EED Binding Against Human Malignant Melanoma Cells
Source: Int J Mol Sci. 2026 Mar 13;27(6):2647. doi: 10.3390/ijms27062647 (PMC13026104; doi:10.3390/ijms27062647)
Supplement: Supplementary file 1 [file ijms-27-02647-s001.zip › Revised Supplementary I Gorbunov et al.pdf]

Supplementary Material – Part I

NMR Spectra

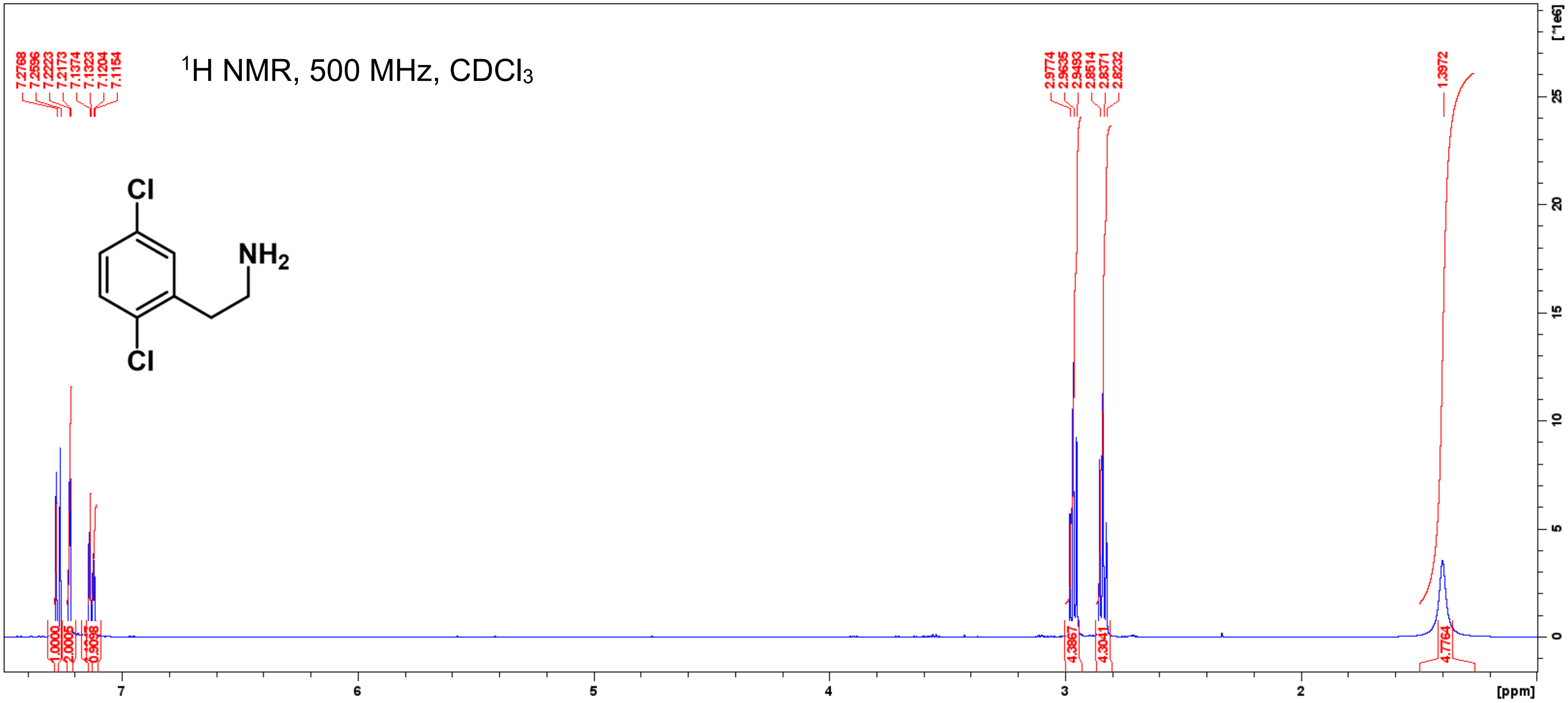

Figure S1. ^{1}H-NMR (500 MHz) spectra of *2-(2,5-dichlorophenethyl)ethanamine* in $CDCl_3$. Chemical shifts δ (ppm) and integrals (arbitrary units) of the signals are depicted in red color.

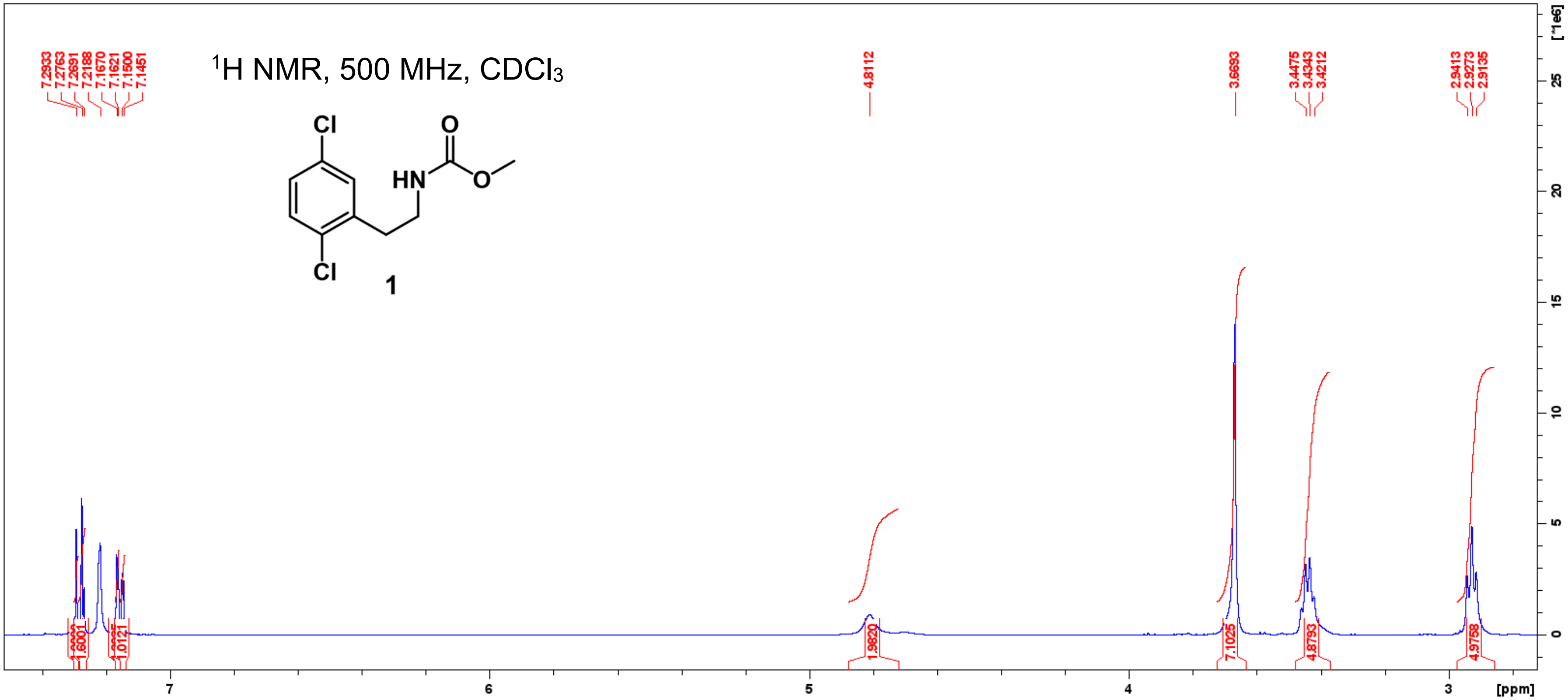

Figure S2. ^{1}H-NMR (500 MHz) spectra of *methyl (2,5-dichlorophenethyl)carbamate* in $CDCl_3$. Chemical shifts δ (ppm) and integrals (arbitrary units) of the signals are depicted in red color.

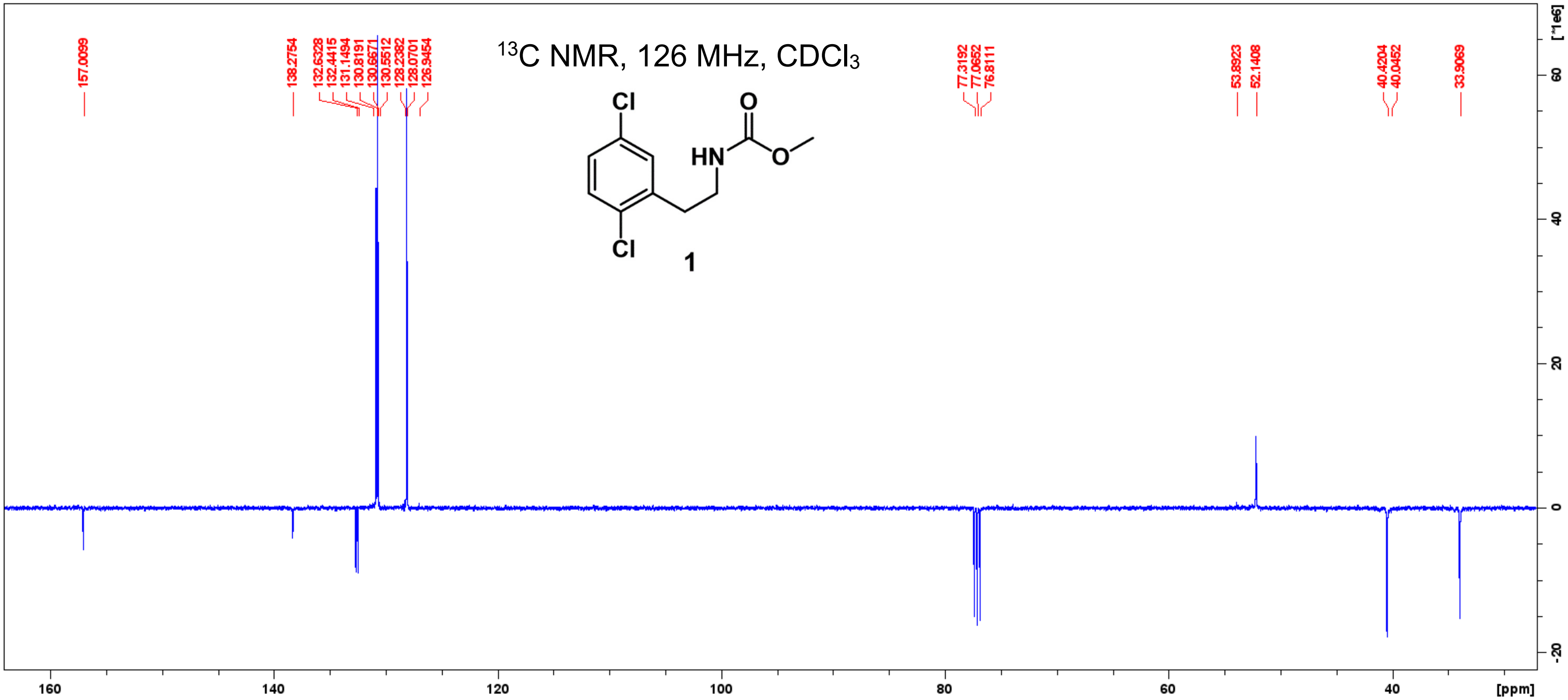

Figure S3. ^{13}C-NMR (126 MHz) spectra of *methyl (2,5-dichlorophenethyl)carbamate* in $CDCl_3$. Chemical shifts δ (ppm) of the signals are depicted in red color.

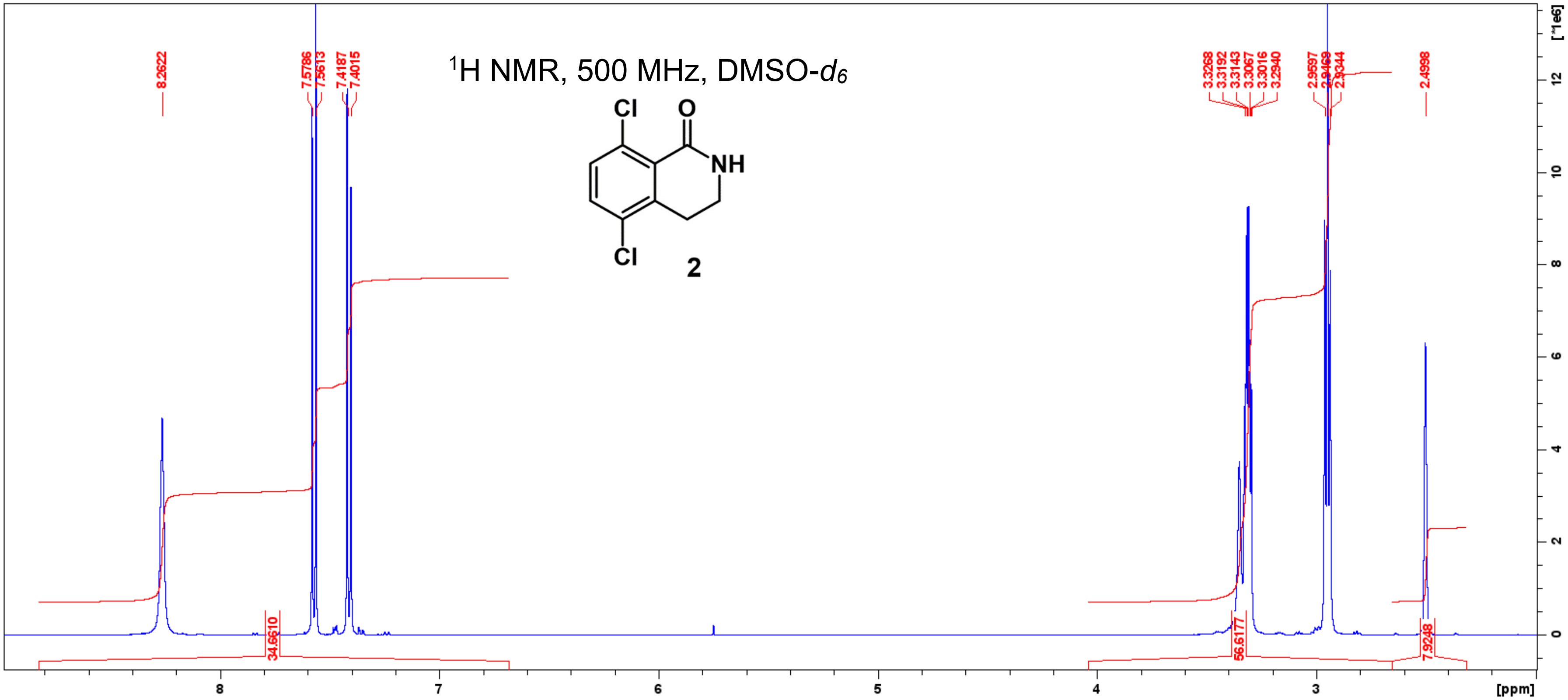

Figure S4. ^{1}H-NMR (500 MHz) spectra of*5,8-dichloro-3,4-dihydroisoquinolin-1(2H)-one* in DMSO-d_6. Chemical shifts δ (ppm) and integrals (arbitrary units) of the signals are depicted in red color.

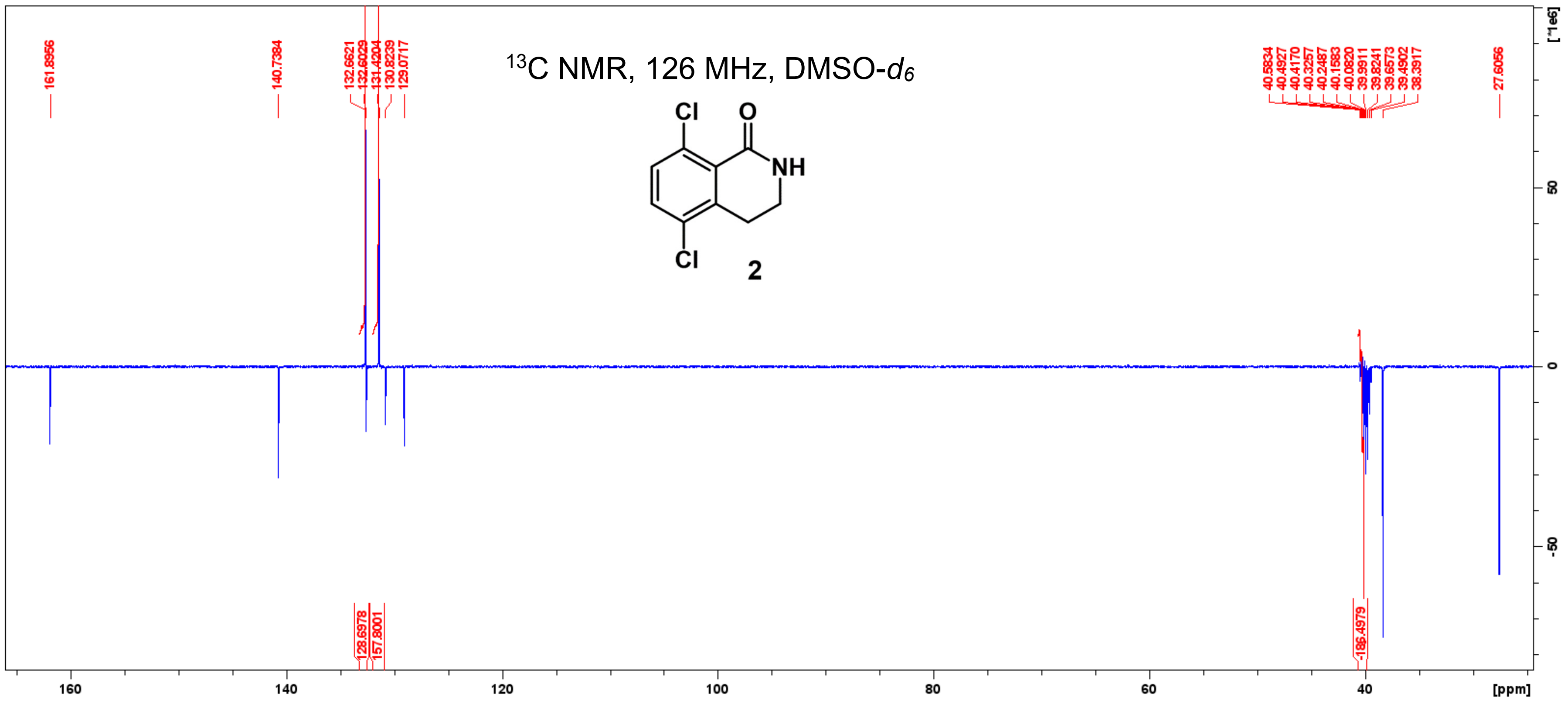

Figure S5. ^{13}C-NMR (126 MHz) spectra of *5,8-dichloro-3,4-dihydroisoquinolin-1(2H)-one* in DMSO-d_6. Chemical shifts δ (ppm) and integrals (arbitrary units) of the signals are depicted in red color.

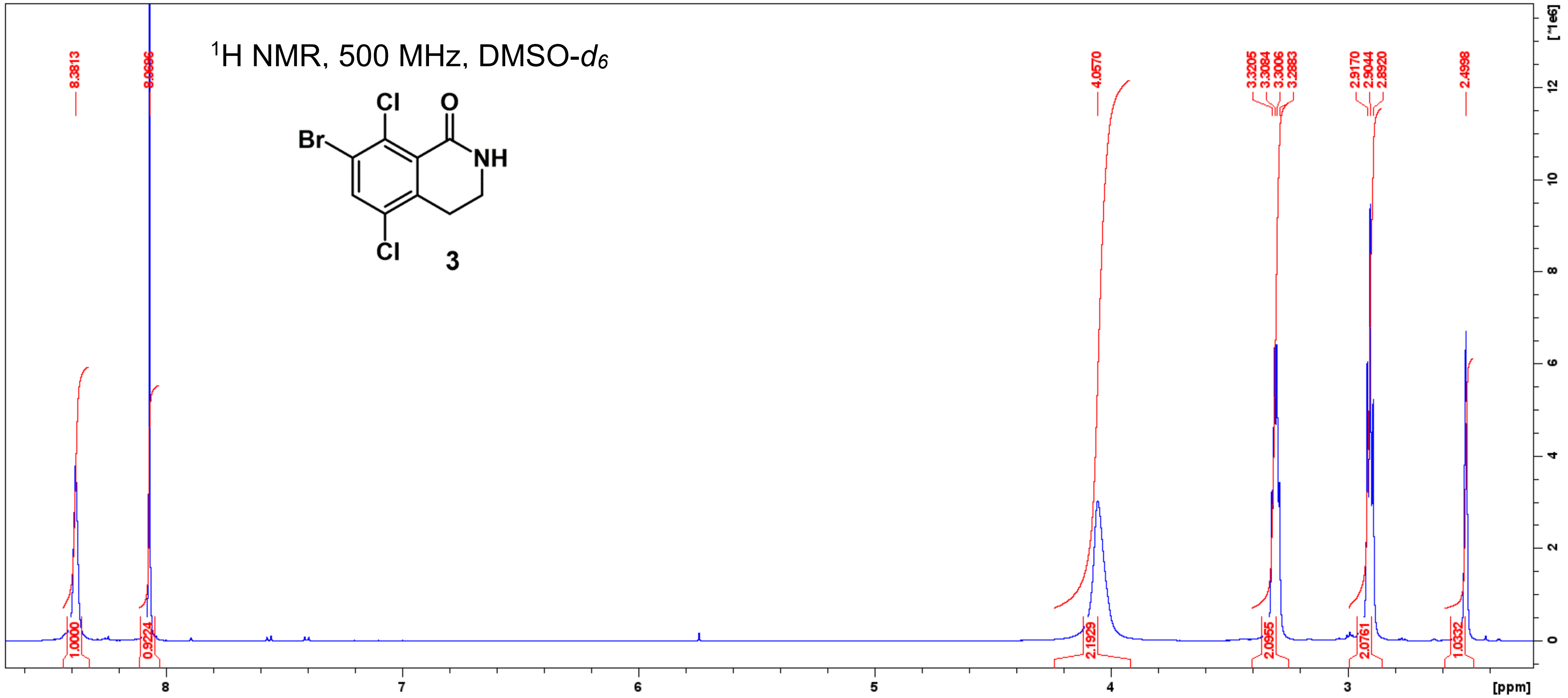

Figure S6. ^{1}H-NMR (500 MHz) spectra of *7-bromo-5,8-dichloro-3,4-dihydroisoquinolin-1(2H)-one* in DMSO-d_6. Chemical shifts δ (ppm) and integrals (arbitrary units) of the signals are depicted in red color.

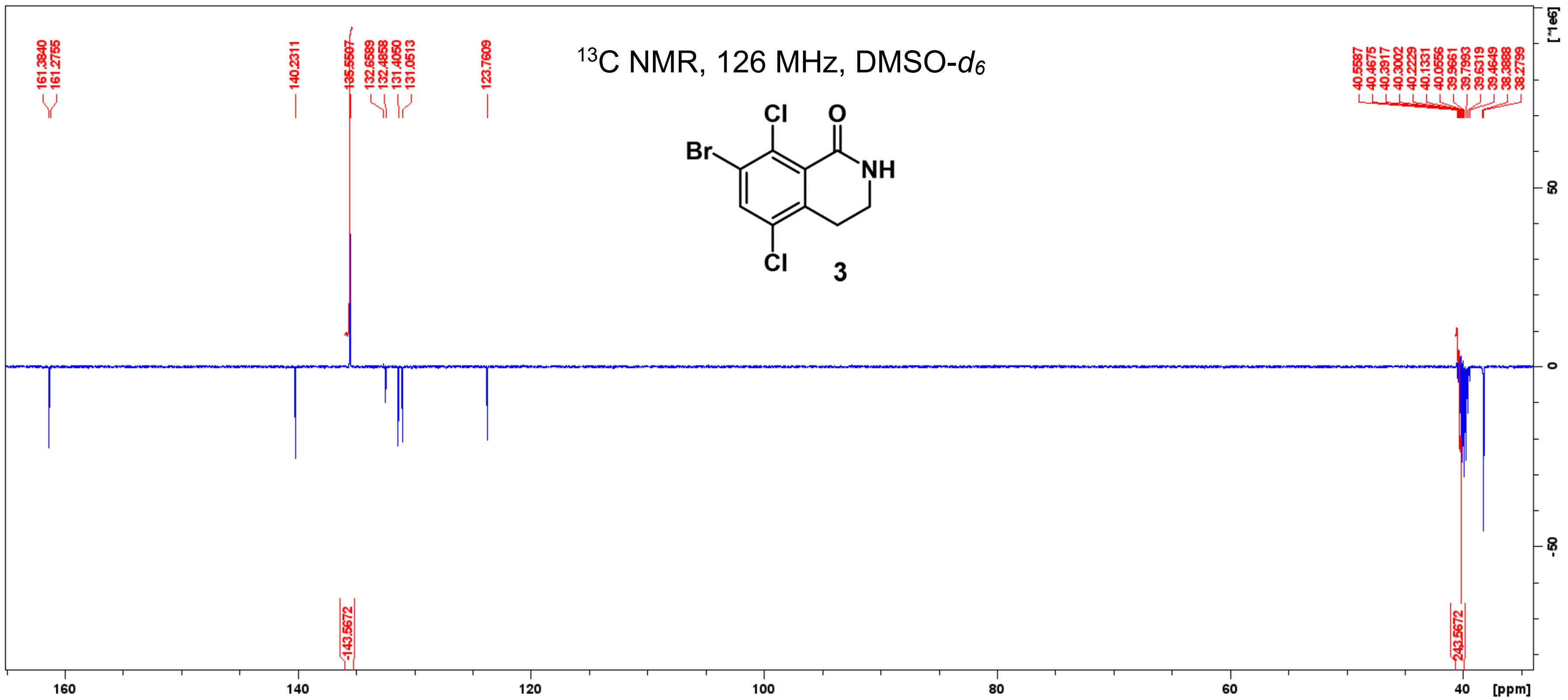

Figure S7. ^{13}C-NMR (126 MHz) spectra of *7-bromo-5,8-dichloro-3,4-dihydroisoquinolin-1(2H)-one* in DMSO-d_6. Chemical shifts δ (ppm) and integrals (arbitrary units) of the signals are depicted in red color.

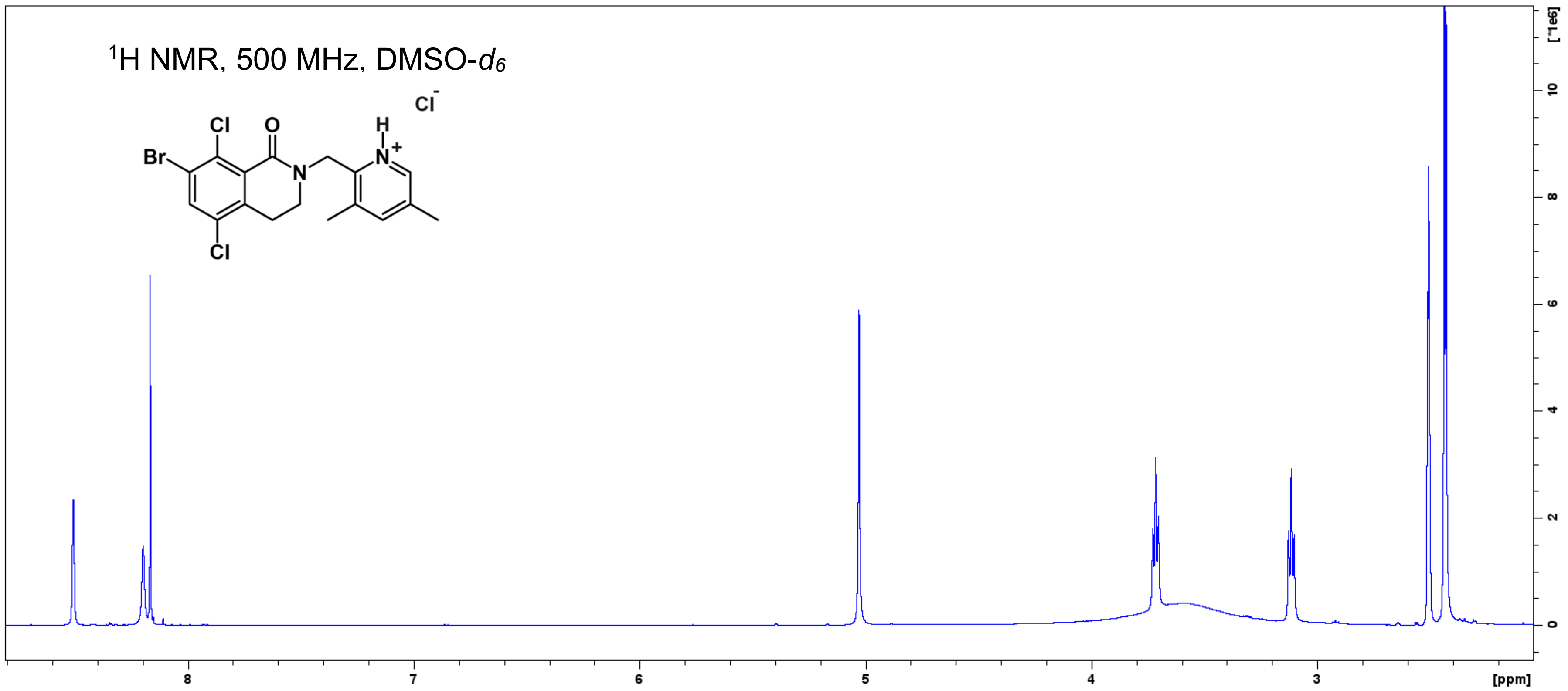

Figure S8. ¹H-NMR (500 MHz) spectra of *7-bromo-5,8-dichloro-2-((3,5-dimethylpyridin-2-yl)methyl)-3,4-dihydroisoquinolin-1(2H)-onehydrochloride* in DMSO-d_6.

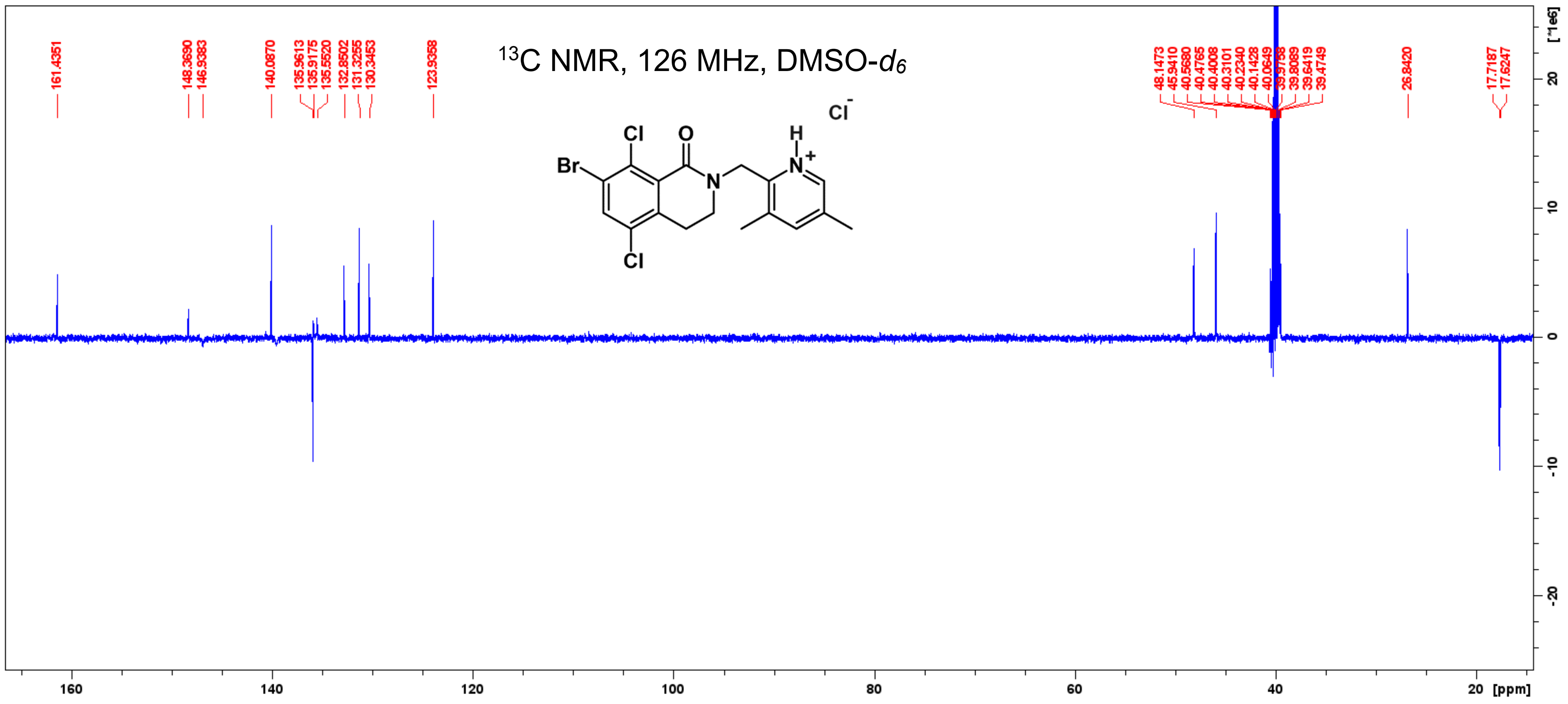

Figure S9. ^{13}C-NMR (126 MHz) spectra of *7-bromo-5,8-dichloro-2-((3,5-dimethylpyridin-2-yl)methyl)-3,4-dihydroisoquinolin-1(2H)-one hydrochloride* in DMSO-d_6. Chemical shifts δ (ppm) of the signals are depicted in red color.

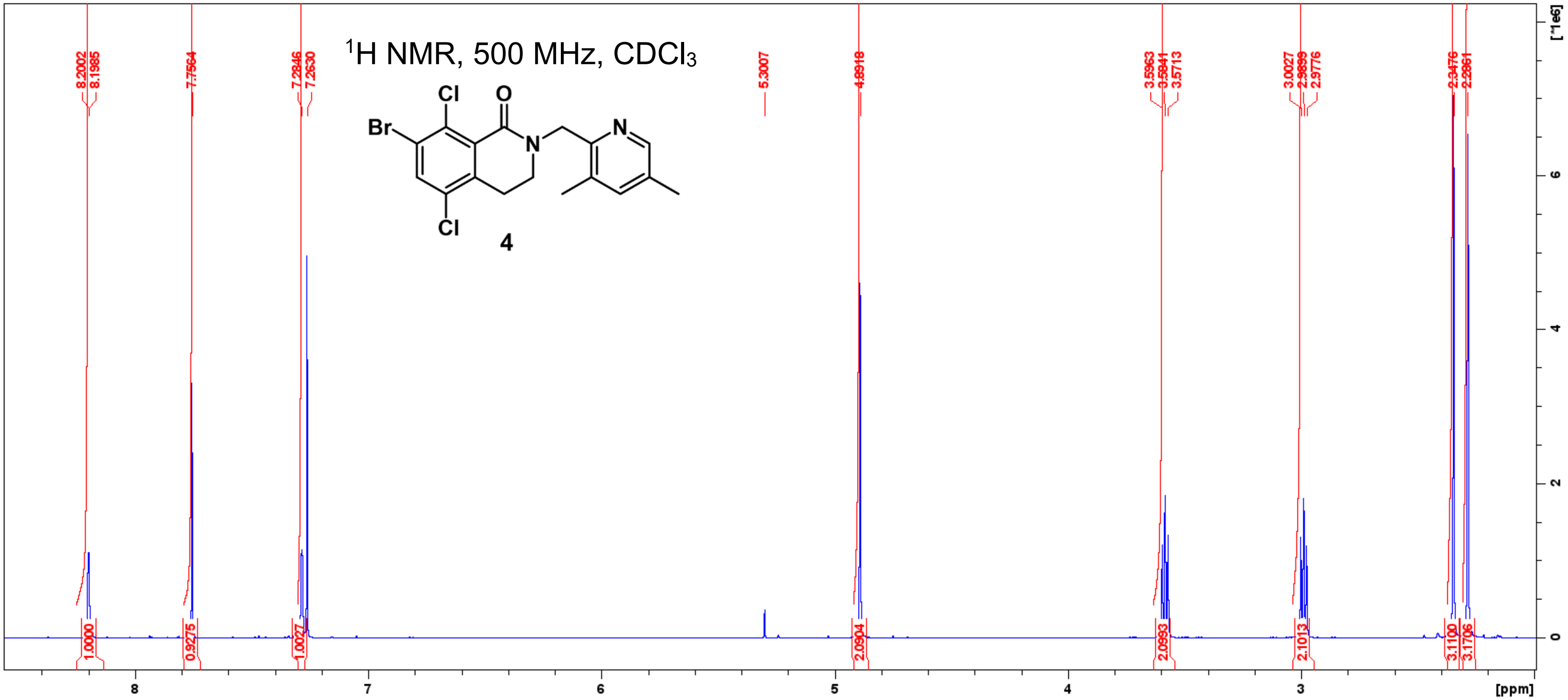

Figure S10. ^{1}H-NMR (500 MHz) spectra of *7-bromo-5,8-dichloro-2-((3,5-dimethylpyridin-2-yl)methyl)-3,4-dihydroisoquinolin-1(2H)-one* (**4**) in $CDCl_3$. Chemical shifts δ (ppm) and integrals (arbitrary units) of the signals are depicted in red color.

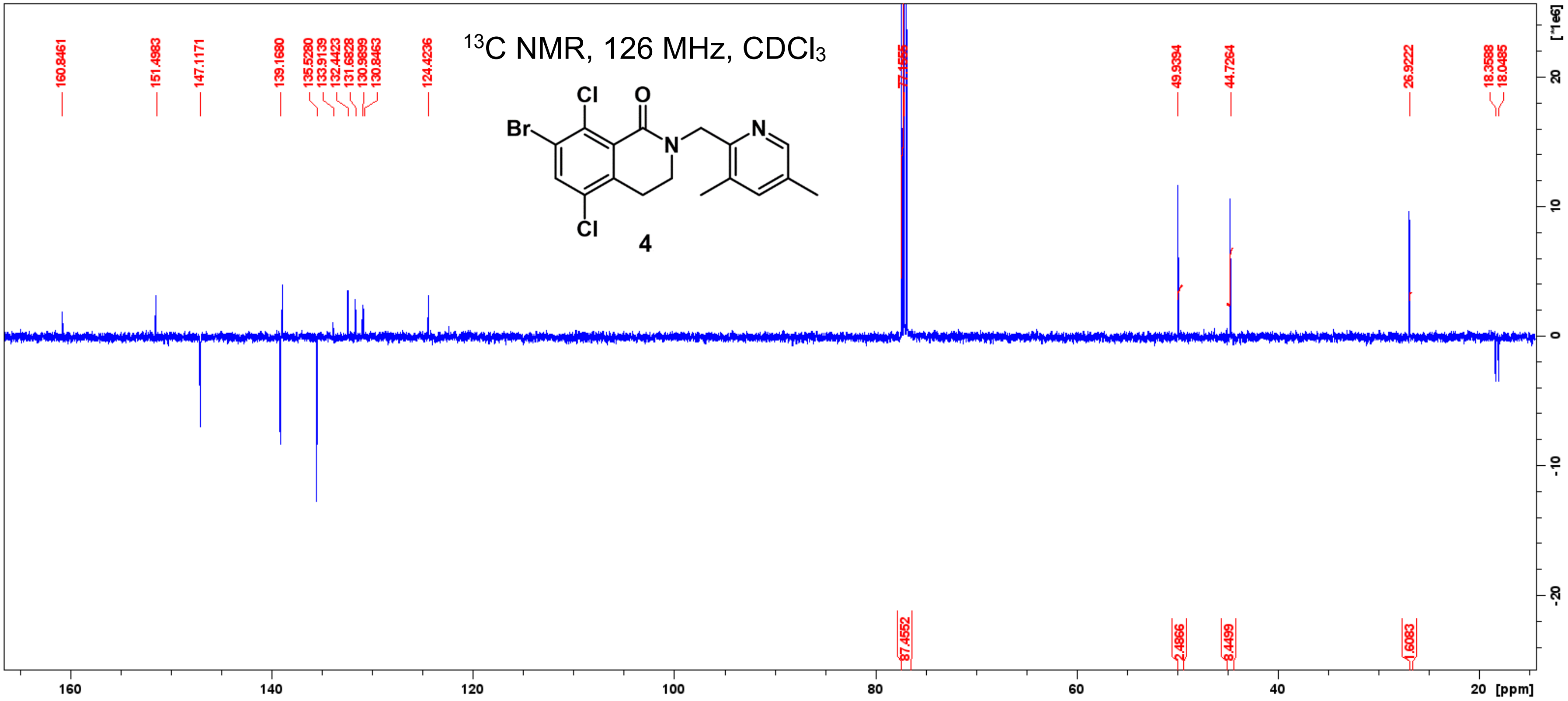

Figure S11. ^{13}C-NMR (126 MHz) spectra of *7-bromo-5,8-dichloro-2-((3,5-dimethylpyridin-2-yl)methyl)-3,4-dihydroisoquinolin-1(2H)-one*(**4**) **in** $CDCl_3$. Chemical shifts δ (ppm) and integrals (arbitrary units) of the signals are depicted in red color.

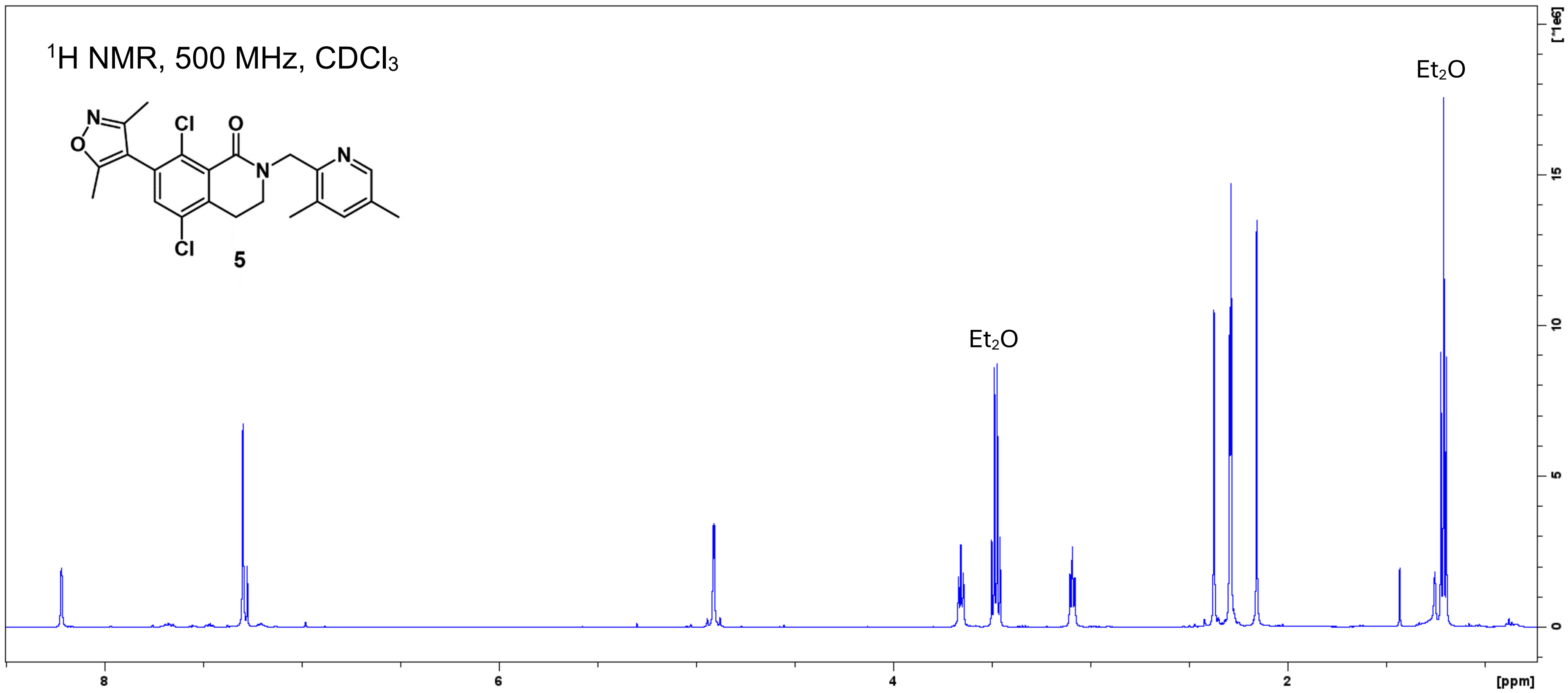

Figure S12. ^{1}H-NMR (500 MHz) spectra of *5,8-dichloro-2-((3,5-dimethylpyridin-2-yl)methyl)-7-(3,5-dimethylisoxazol-4-yl)-3,4-dihydroisoquinolin-1(2H)-one*(**5**) in CDCl$_3$.

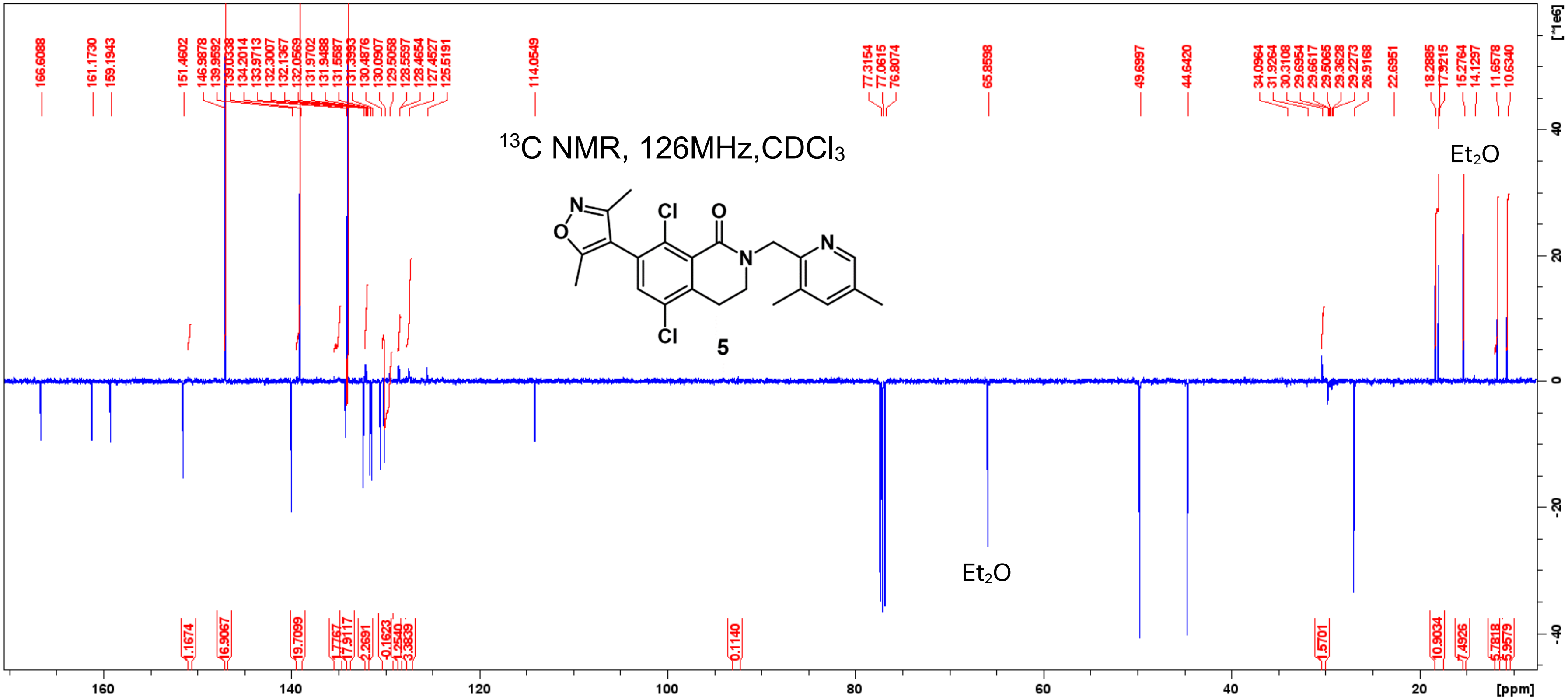

Figure S13. ^{13}C-NMR (126 MHz) spectra of *5,8-dichloro-2-((3,5-dimethylpyridin-2-yl)methyl)-7-(3,5-dimethylisoxazol-4-yl)-3,4-dihydroisoquinolin-1(2H)-one)* (**5**) in $CDCl_3$. Chemical shifts δ (ppm) and integrals (arbitrary units) of the signals are depicted in red color.

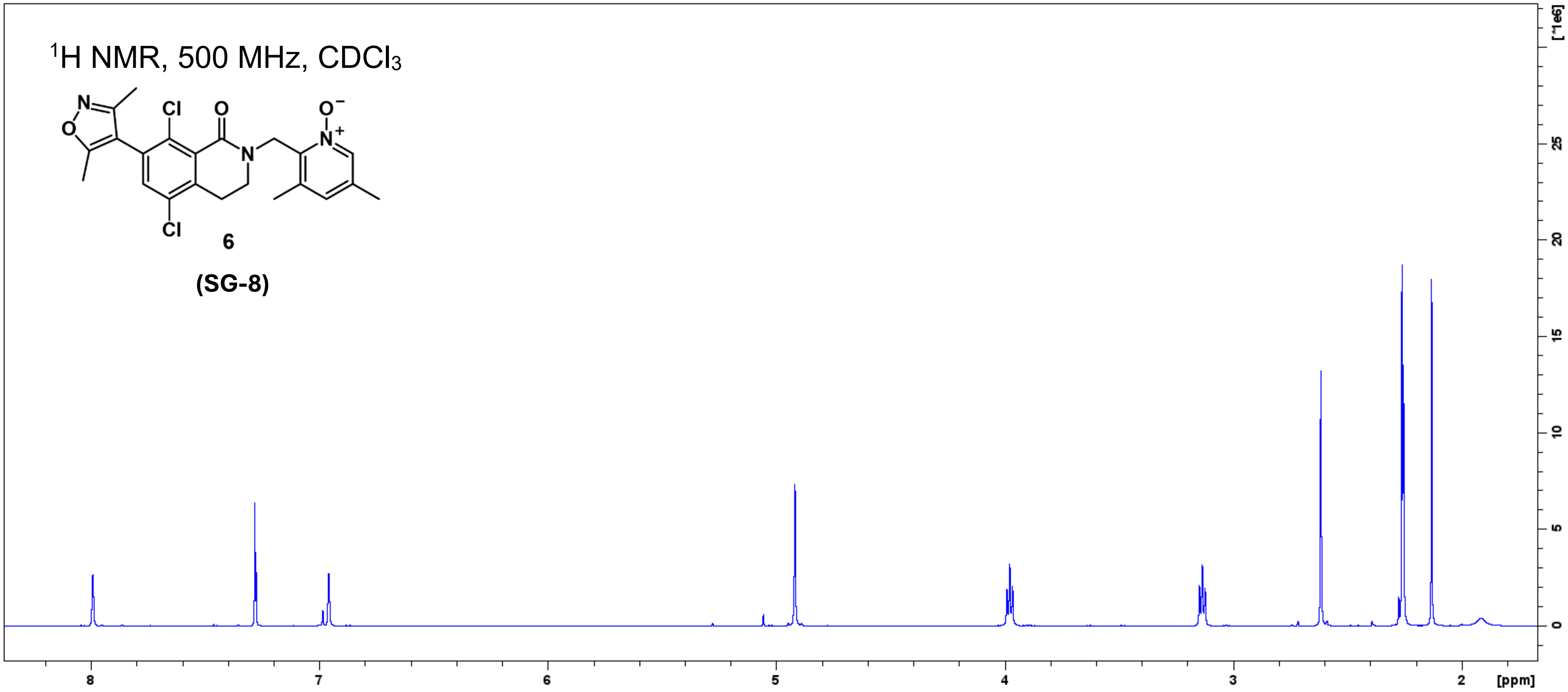

Figure S14. ^1H-NMR (500 MHz) spectra of *5,8-dichloro-2-[(3,5-dimethyl-1-oxo-1λ⁵-pyridin-2-yl)methyl]-7-(3,5-dimethylisoxazol-4-yl)-3,4-dihydroisoquinolin-1(2H)-one* (**6**, SG-8) in $CDCl_3$.

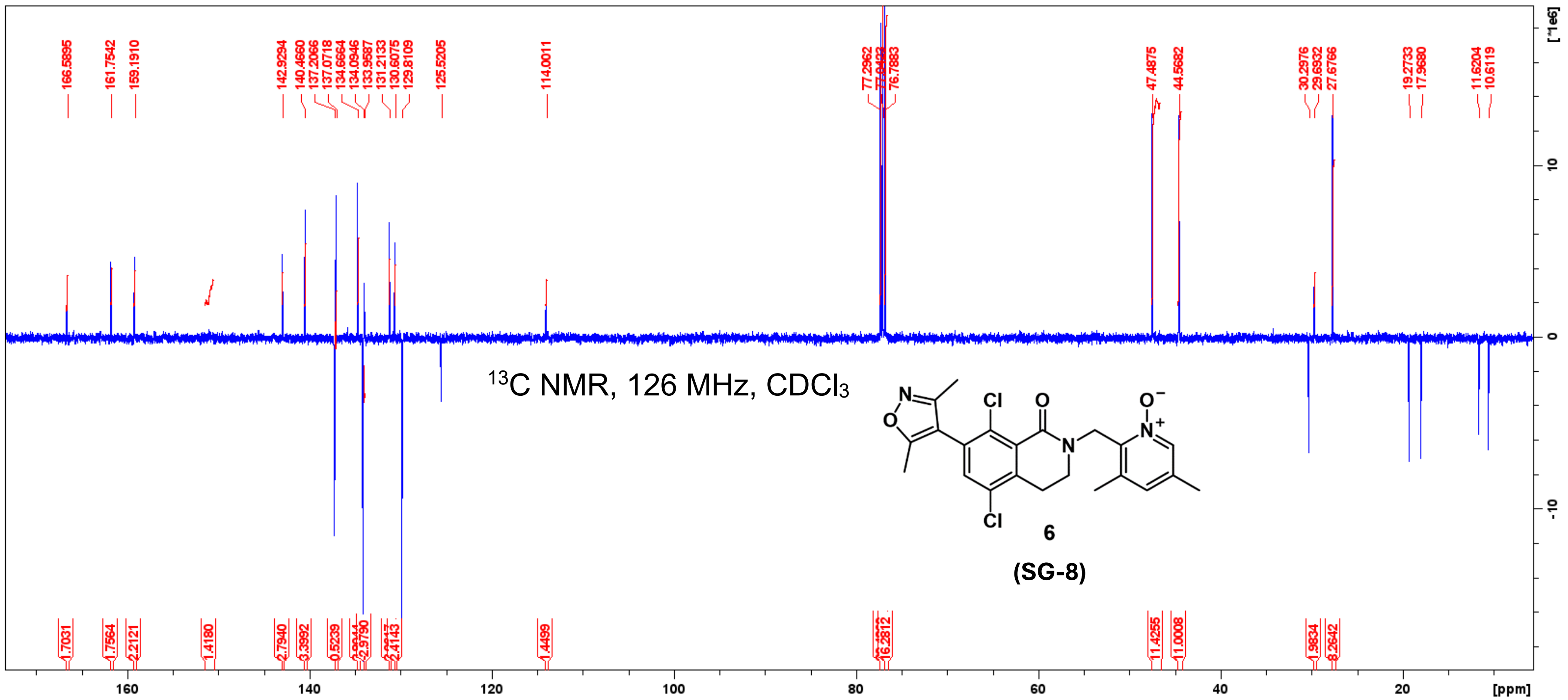

Figure S15. ^{13}C-NMR (126 MHz) spectra of *5,8-dichloro-2-[(3,5-dimethyl-1-oxo-1λ⁵-pyridin-2-yl)methyl]-7-(3,5-dimethylisoxazol-4-yl)-3,4-dihydroisoquinolin-1(2H)-one* (**6**, SG-8) in $CDCl_3$. Chemical shifts δ (ppm) and integrals (arbitrary units) of the signals are depicted in red color.

HRMS Spectra

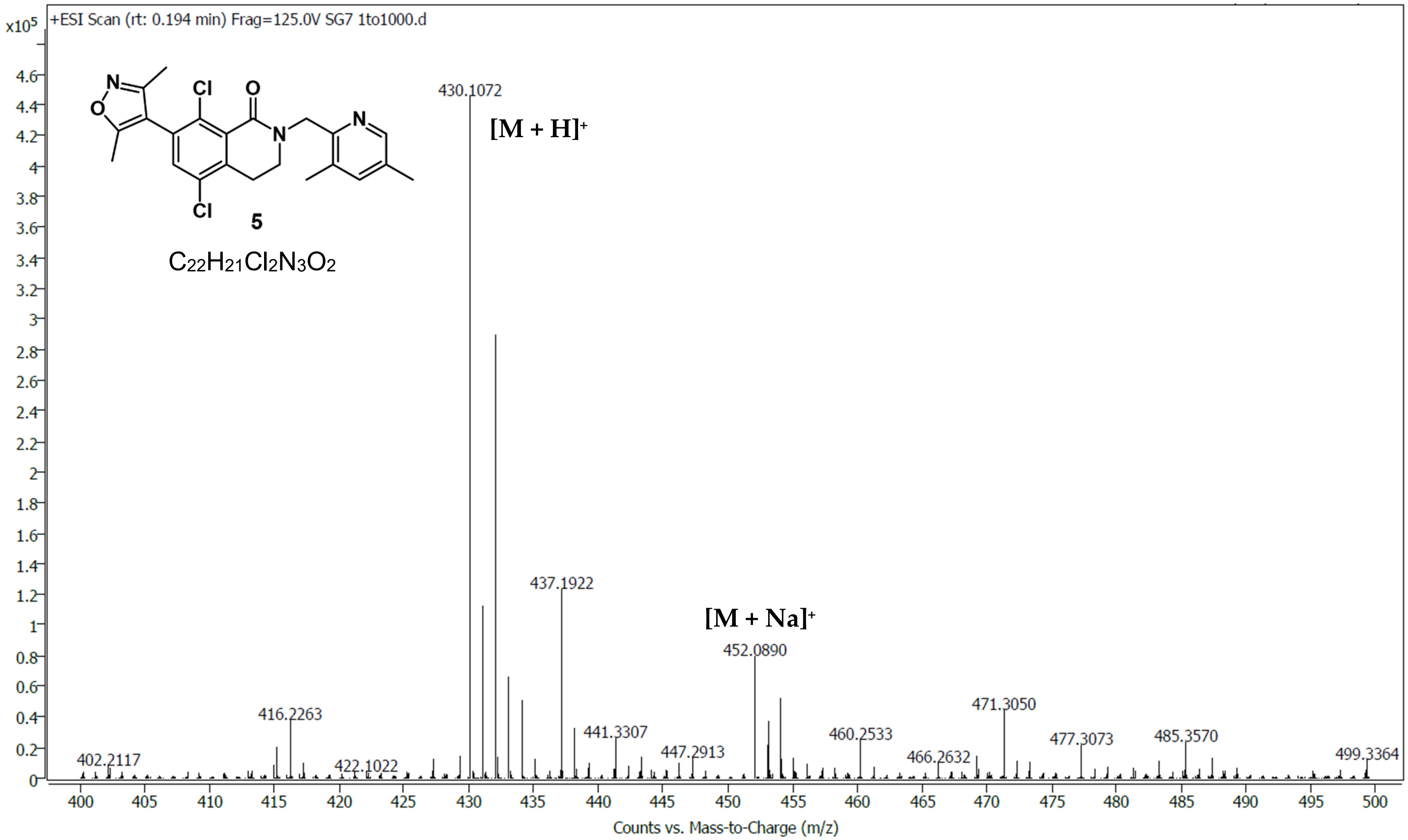

Figure S16. HRMS (ESI) spectra of *5,8-dichloro-2-((3,5-dimethylpyridin-2-yl)methyl)-7-(3,5-dimethylisoxazol-4-yl)-3,4-dihydroisoquinolin-1(2H)-one* (**5**) in acetonitrile.

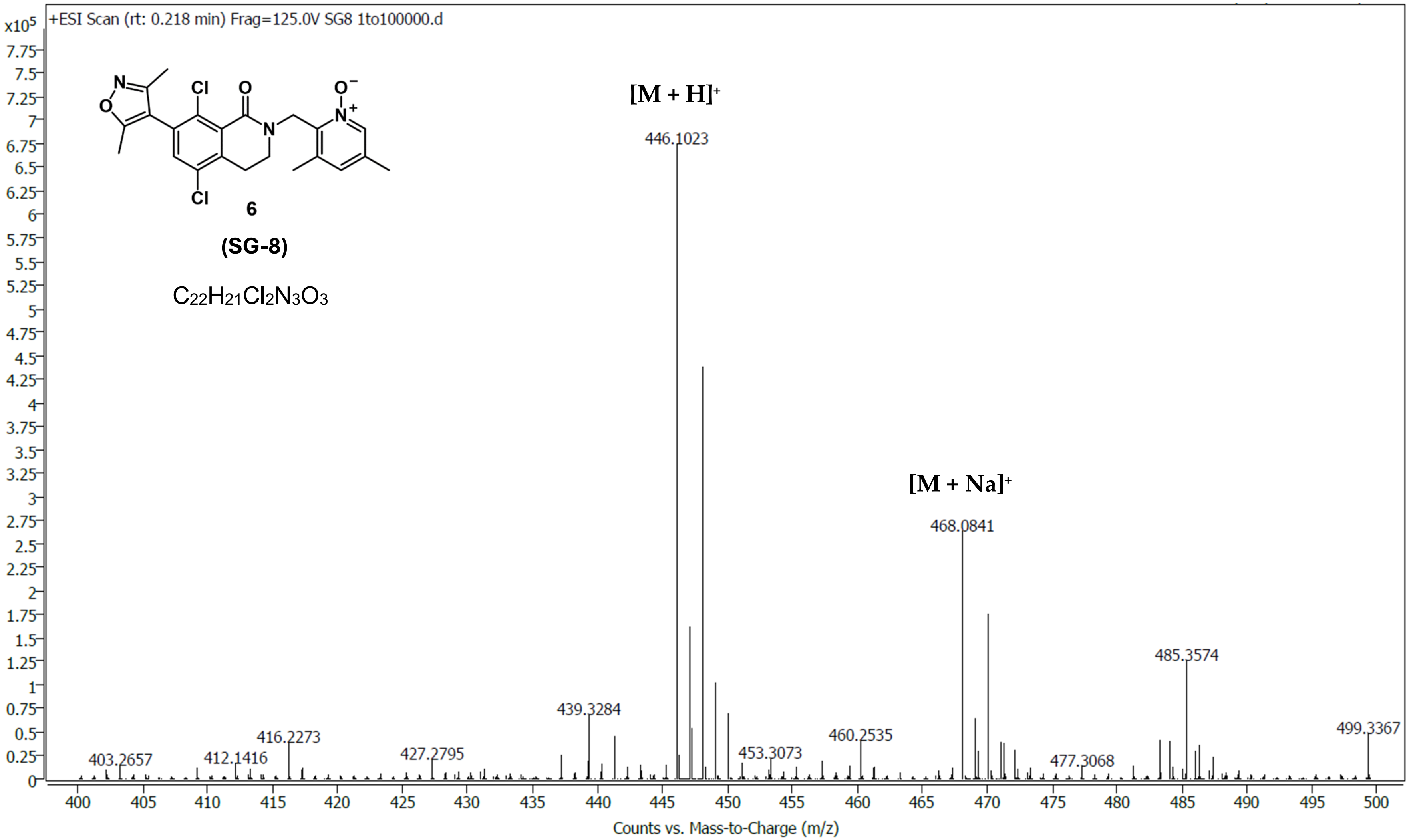

Figure S17. HRMS (ESI) spectra of*5,8-dichloro-2-[(3,5-dimethyl-1-oxo-1λ⁵-pyridin-2-yl)methyl]-7-(3,5-dimethylisoxazol-4-yl)-3,4-dihydroisoquinolin-1(2H)-one* (**6**, SG-8) in acetonitrile.

Molecular Docking Data

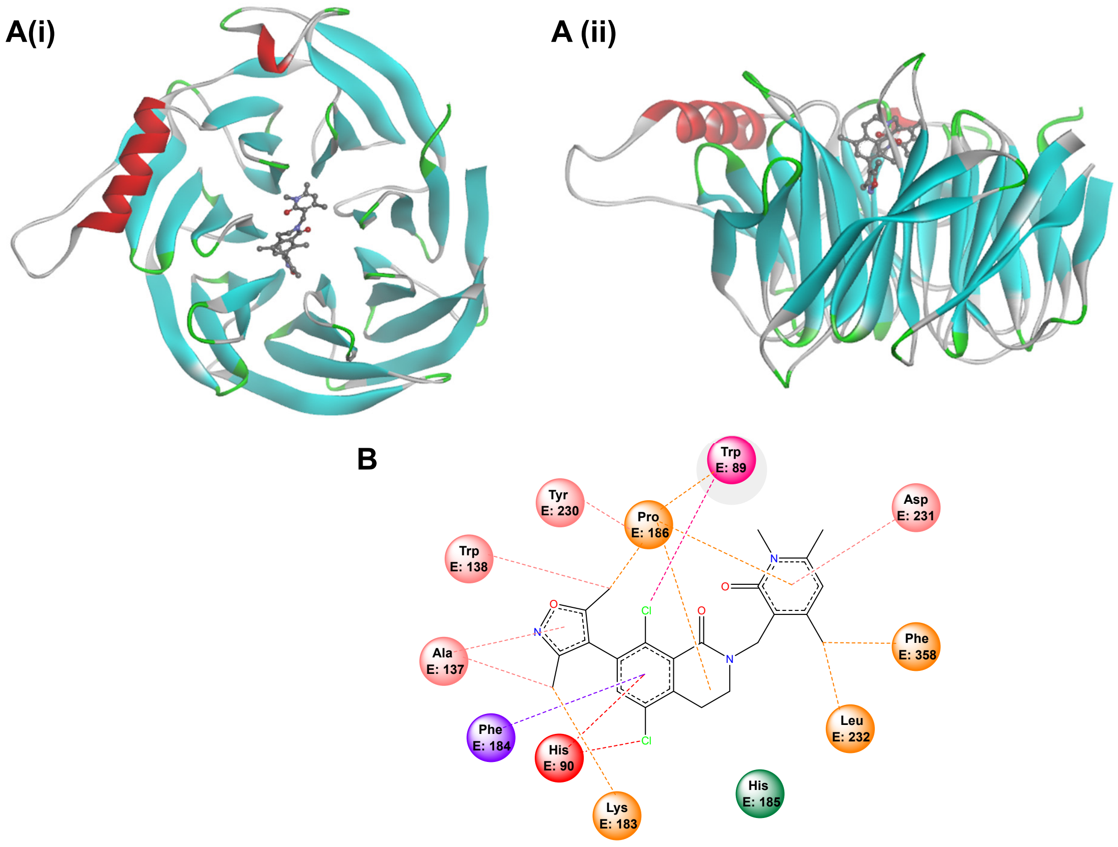

TDI-6118

Figure S18. Docking calculations showing interactions of TDI-6118 [**A** (**i**), (**ii**)] with the bottom pocket of EED. The binding of the ligand to the entire EED subunit can be observed from the front [**A** (**i**)] and side [**A** (**ii**)]. (**B**) schematically represents the local interactions of the ligand with the amino acid residues in the binding site (chain E).

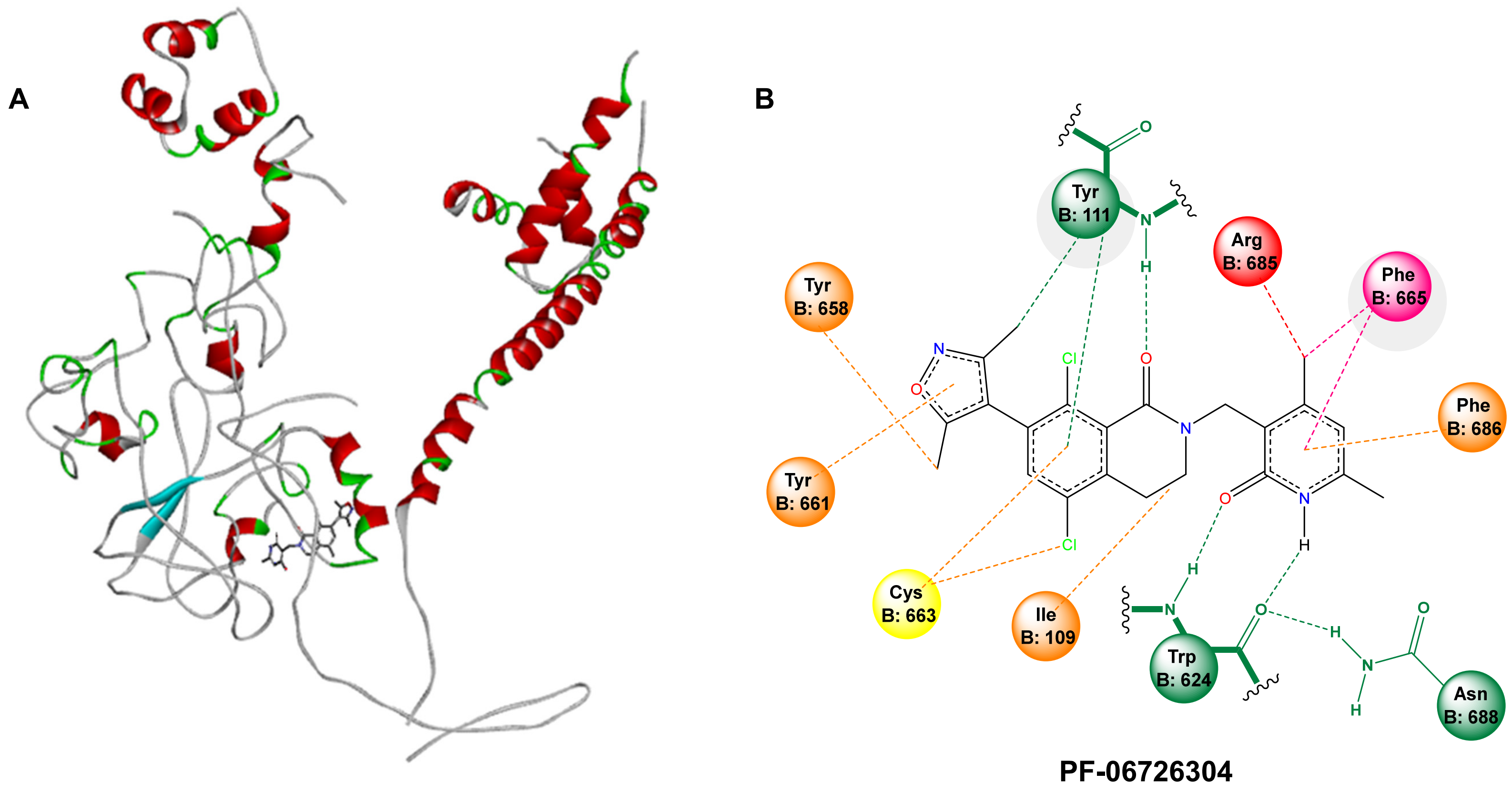

Figure S19. Docking calculations showing interactions of PF-06726304 with the SET domain of EZH2. (**A**) The binding of the ligand to the entire EZH2 subunit can be observed. (**B)** schematically represents the local interactions of the ligand with the amino acid residues in the binding site (chain B). PF-06726304 demonstrated the highest affinity, due to its strong hydrogen-bond interactions with Trp-624 and Tyr-111 (green dashed lines) and favorable π–π contacts with aromatic residues such as Phe-665, Phe-686, and Tyr-661 (orange dashed lines). This calculation corresponds to the experimental X-ray structures (PDB ID: *5IJ7*, *6B3W*).

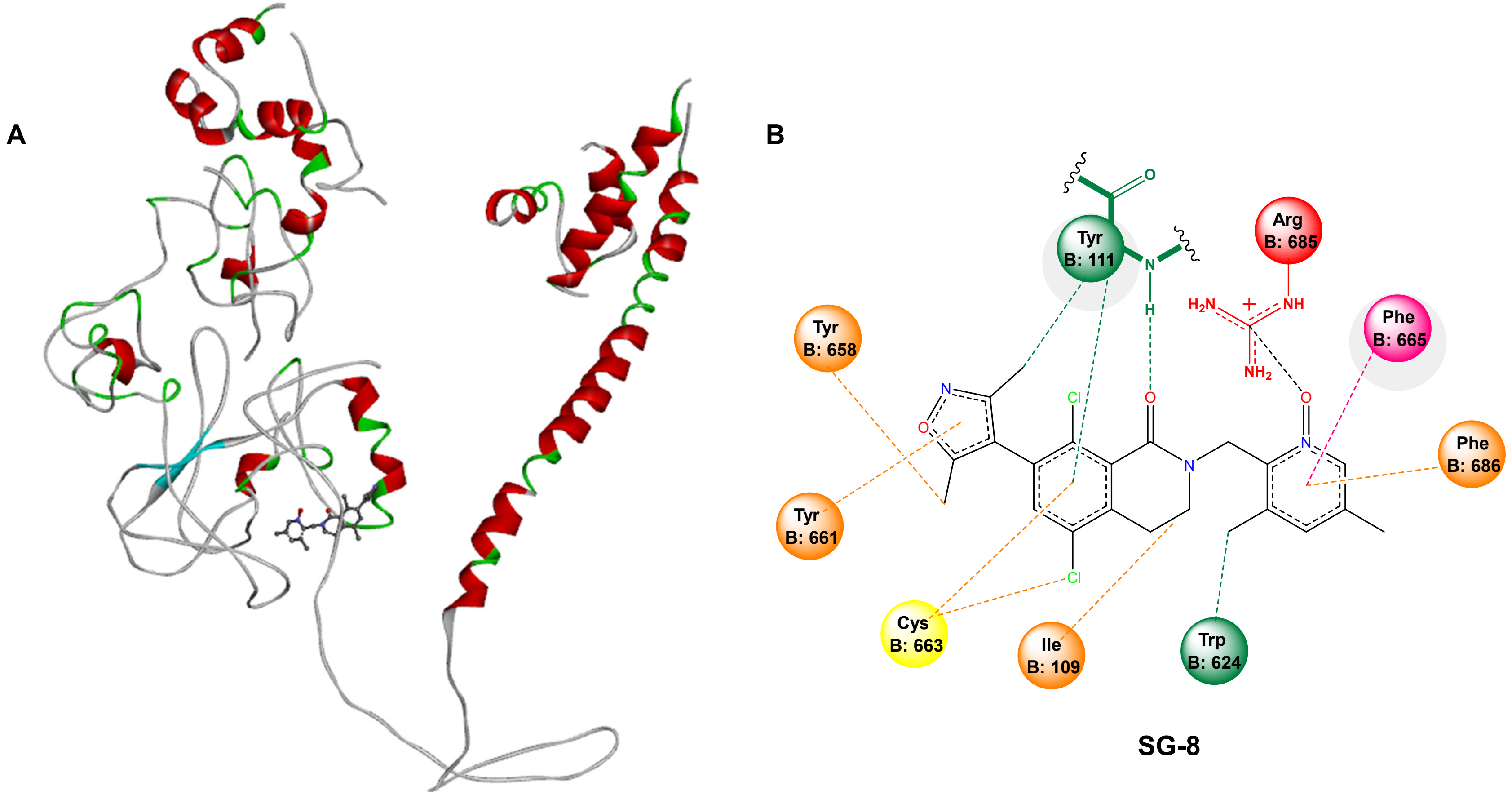

Figure S20. Docking calculations showing interactions of SG-8 with the SET domain of EZH2. (**A**) The binding of the ligand to the entire EZH2 subunit can be observed. (**B)** schematically represents the local interactions of the ligand with the amino acid residues in the binding site (chain B). In contrast to PF-06726304, SG-8 did not show any hydrogen bonds with Trp-624. Instead, an ionic attraction between the oxygen atom of the *N*-oxide group and the Arg-685 residue was predicted. Favorable π–π contacts with aromatic residues Phe-665, Phe-686, and Tyr-661 and hydrogen bonding with Tyr-111 were also found as in the case of PF-06726304.

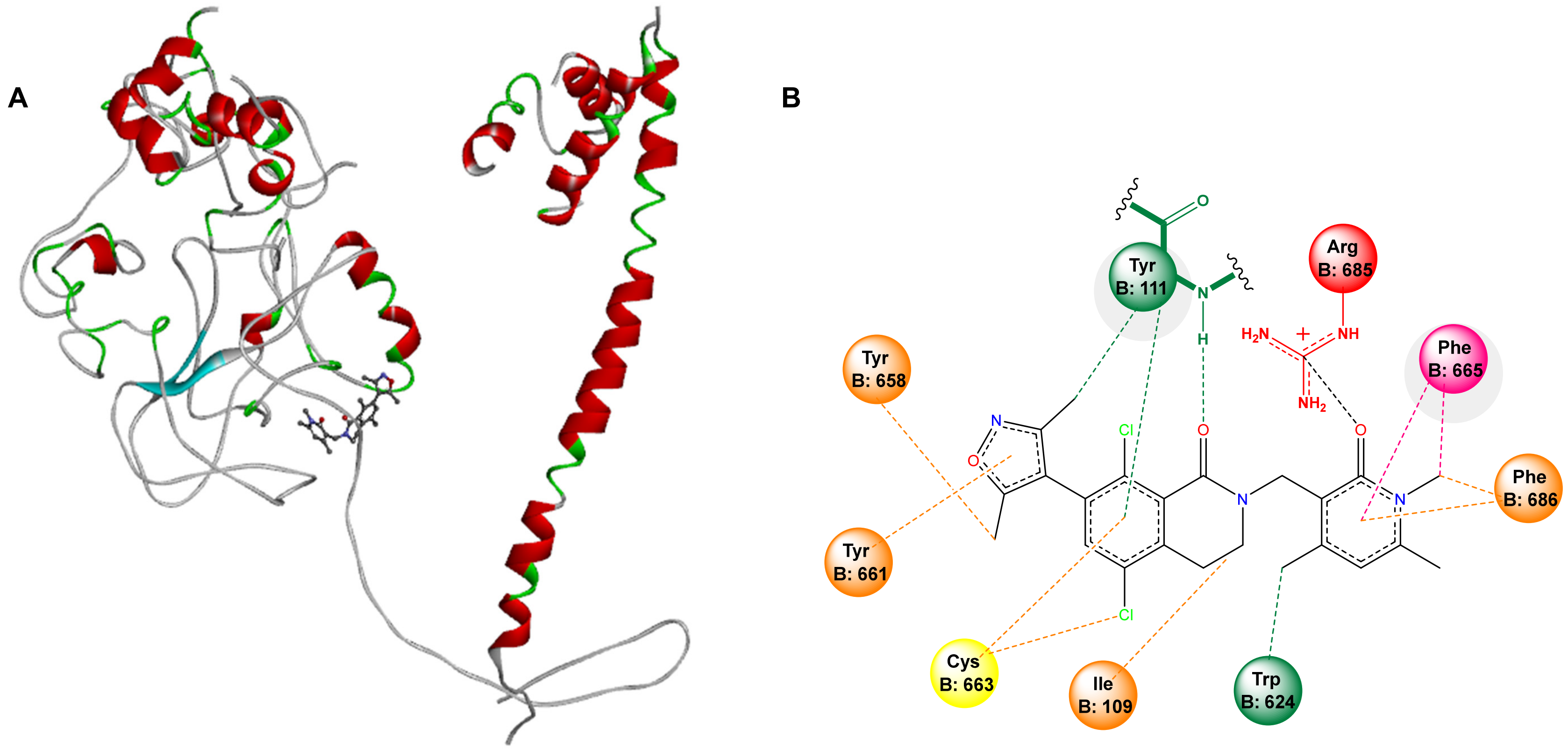

TDI-6118

Figure S21. Docking calculations showing interactions of TDI-6118 with the SET domain of EZH2. (**A**) The binding of the ligand to the entire EZH2 subunit can be observed. (**B)** schematically represents the local interactions of the ligand with the amino acid residues in the binding site (chain B). TDI-6118 had the same mode of interaction with EZH2 as SG-8. However, the interaction with Arg-685 residue was found to be weaker. Favorable π–π contacts with aromatic residues Phe-665, Phe-686, and Tyr-661 and hydrogen bonding with Tyr-111 were also found as in the case of PF-06726304.

Calculated physicochemical properties

Table S1. Calculated physicochemical properties of EZH2is, namely Tazemetostat, PF-06726304, TDI-6118 and SG-8.

Molecule	MW (g/mol)	Lipophilicity (WlogP)	TPSA (Å^2)	H-bond donors	BBB permeant
Tazemetostat	572.74	4.05	86.90	2	No
PF-06726304	446.33	4.24	79.20	1	No
TDI-6118	460.35	4.25	68.34	0	Yes
SG-8	446.33	4.18	71.80	0	Yes

MW — Molecular Weight; WlogP — Logarithm of the octanol-water partition coefficient calculated by the atomistic method of Wildman & Crippen; TPSA — Topological Polar Surface Area of molecules; BBB — Blood Brain Barrier.

Microscopy images

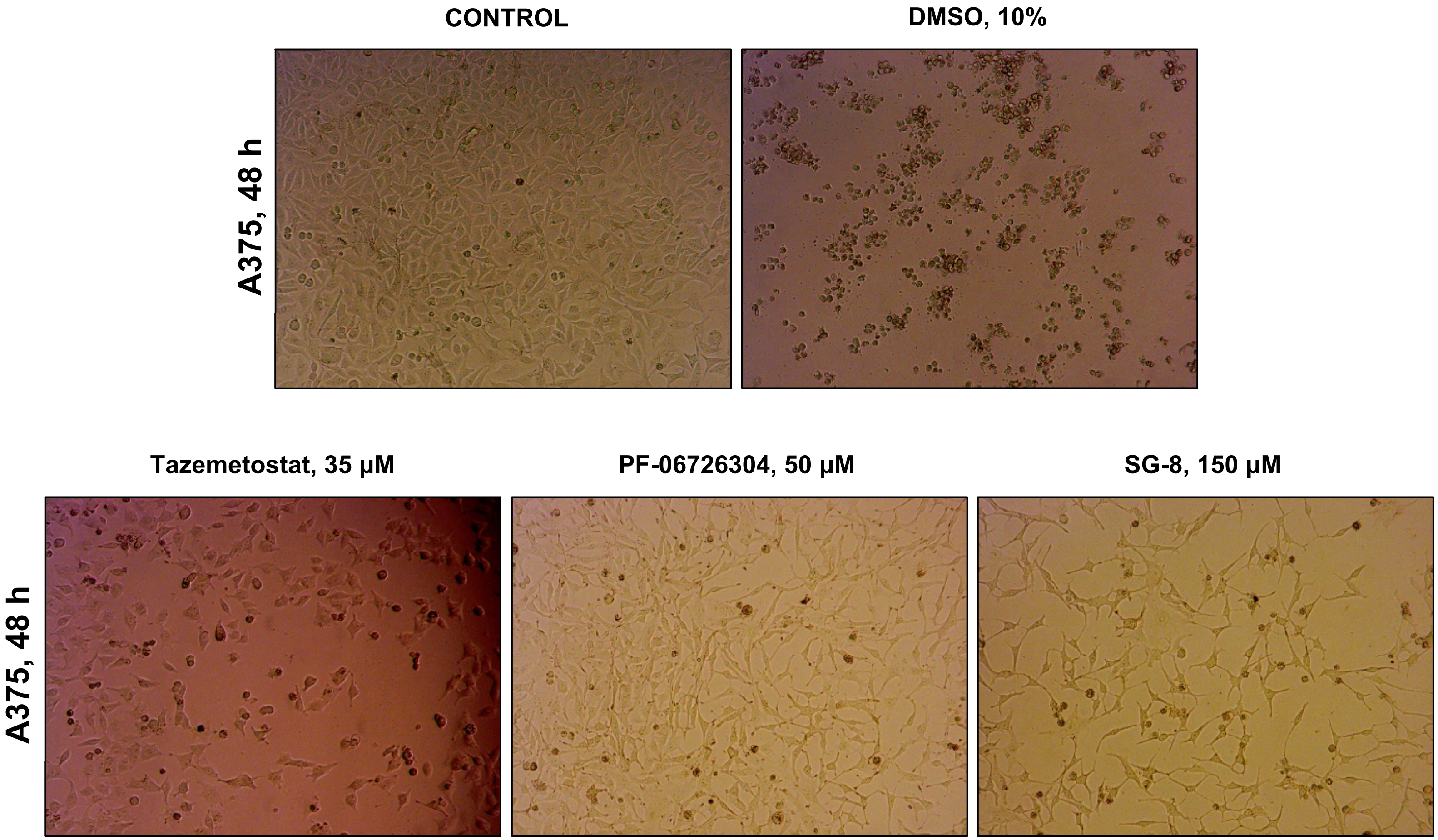

Figure S22. Representative bright-field microscopy images of A375 cells following 48 h treatments with Tazemetostat, PF-06726304 and SG-8 at their respective EC_{50} concentrations. Optical images were acquired using an inverted Kern microscope (The KERN Group, Bensheim, Germany), equipped with a digital camera and 10X lens.
