# Supplementary material for: Biological Evaluation of a Novel Compound with Predicted EZH2 and EED Binding Against Human Malignant Melanoma Cells
Source: Int J Mol Sci. 2026 Mar 13;27(6):2647. doi: 10.3390/ijms27062647 (PMC13026104; doi:10.3390/ijms27062647)
Supplement: Supplementary file 1 [file ijms-27-02647-s001.zip › Revised Supplementary II Gorbunov et al.pdf]

Supplementary Material – Part II

List of primary antibodies

Table S2. List of primary antibodies used for Western blot.

Target	MW (kDa)	Source	Dilution	Supplier	Code
EZH2	98	Rabbit	1:1000	Cell Signaling	D2C9
SUZ12	83	Rabbit	1:1000	Cell Signaling	D39F9
EED	50-70	Rabbit	1:1000	Cell Signaling	E4L6E
H3K27me3	17	Rabbit	1:1000	Cell Signaling	C36B11
p-EZH2 (S21)	98	Rabbit	1:500	Affinity Biosciences	AF3822
Akt	60	Rabbit	1:1000	Cell Signaling	9272
p-Akt (S473)	60	Rabbit	1:2000	Cell Signaling	4060
Caspase-3	17, 19, 35	Rabbit	1:1000	Cell Signaling	9662
PARP	89, 116	Rabbit	1:1000	Cell Signaling	9542
β-Actin	43	Mouse	1:1000	Invitrogen	BA3R

Uncropped Western blot membranes

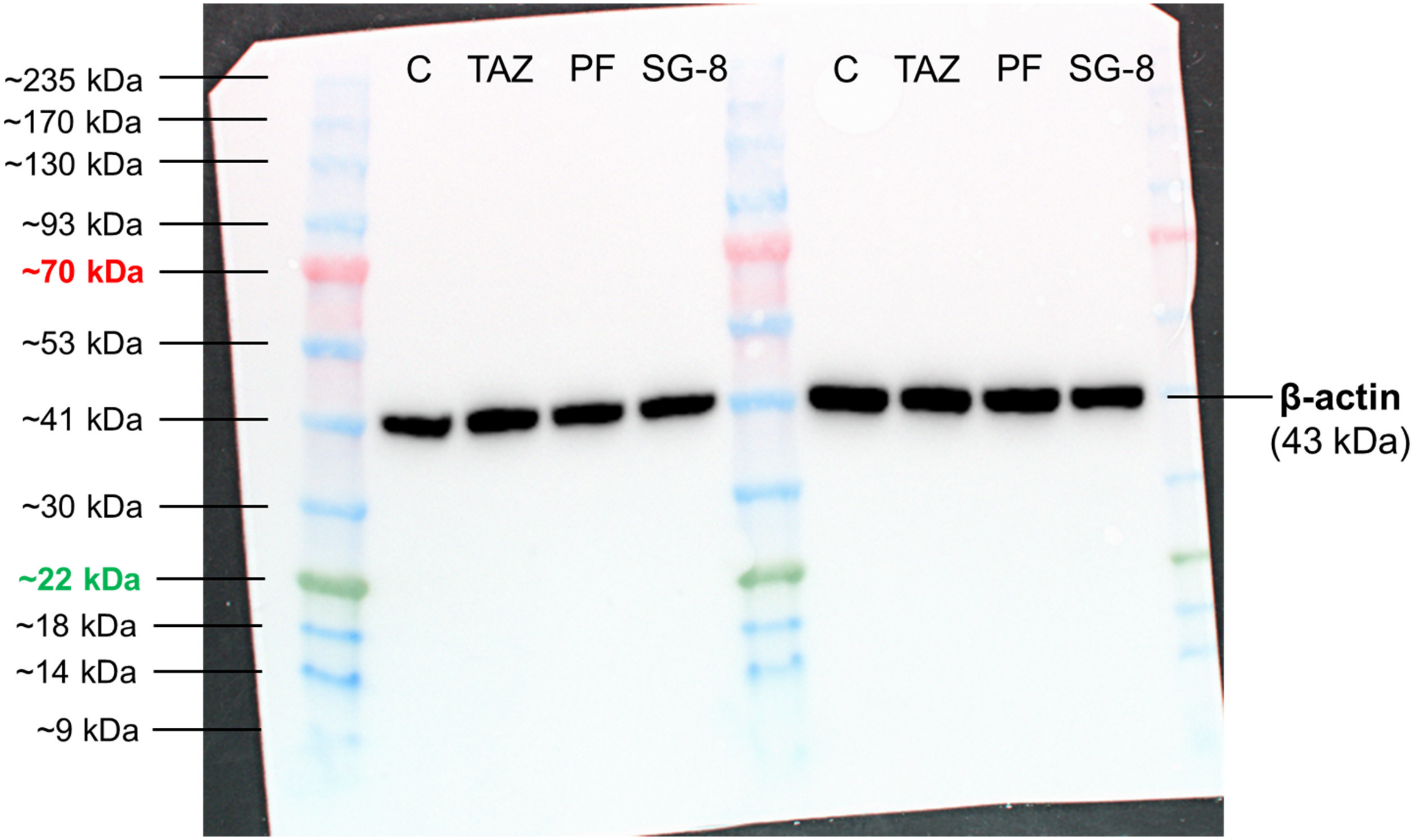

Figure S23. β-actin (43 kDa) antibody. C – Control, TAZ – Tazemetostat, PF – PF-06726304, SG-8 – 5,8-dichloro-2-[(3,5-dimethyl-1-oxo-1λ^5-pyridin-2-yl)methyl]-7-(3,5-dimethylisoxazol-4-yl)-3,4-dihydroisoquinolin-1(2*H*)-one.

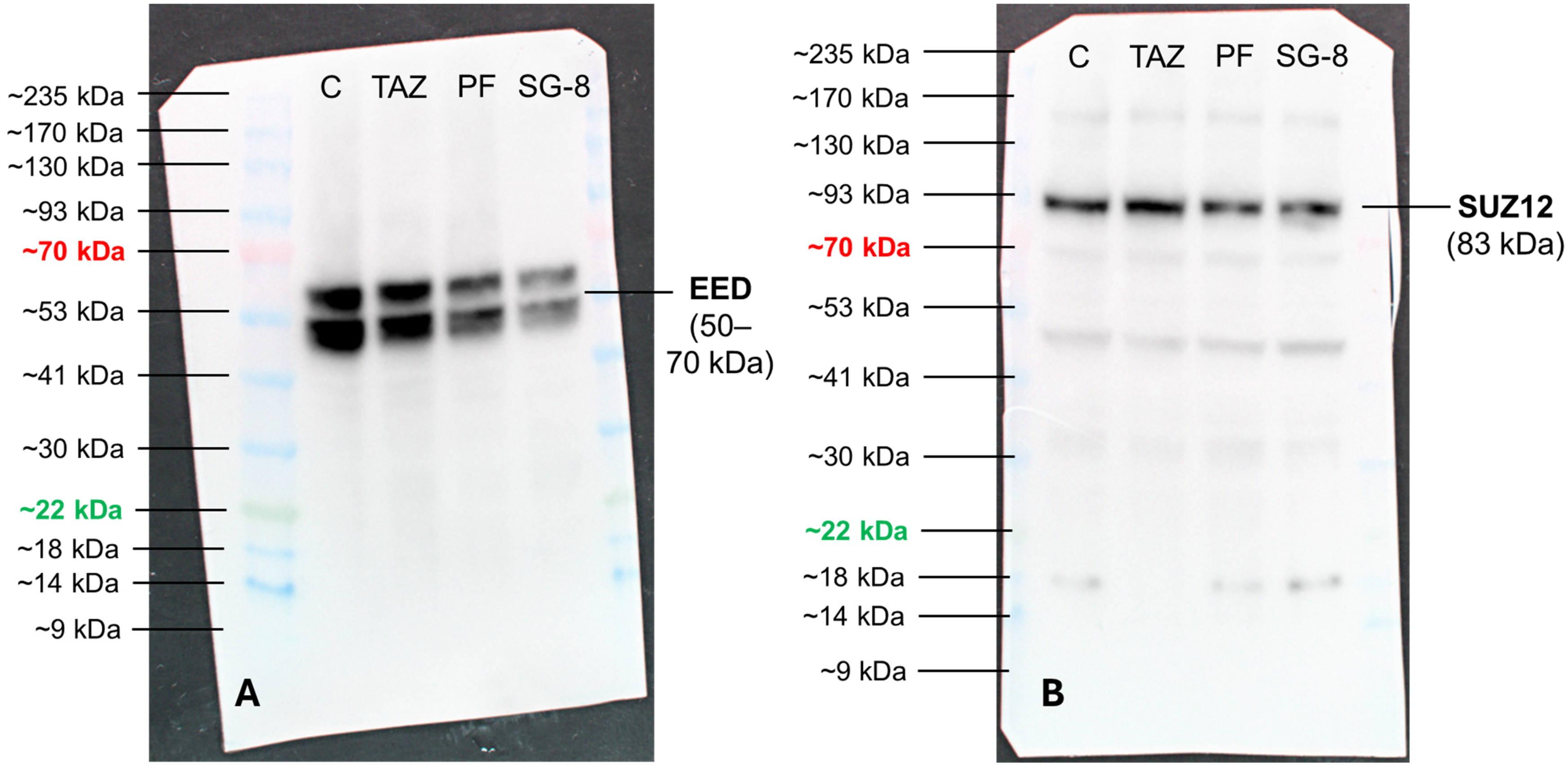

Figure S24. The initial membrane (**Figure S23**) was cut into two pieces (**A** and **B**). **A**: EED (50-70 kDa) antibody. **B**: SUZ12 (83 kDa) antibody.

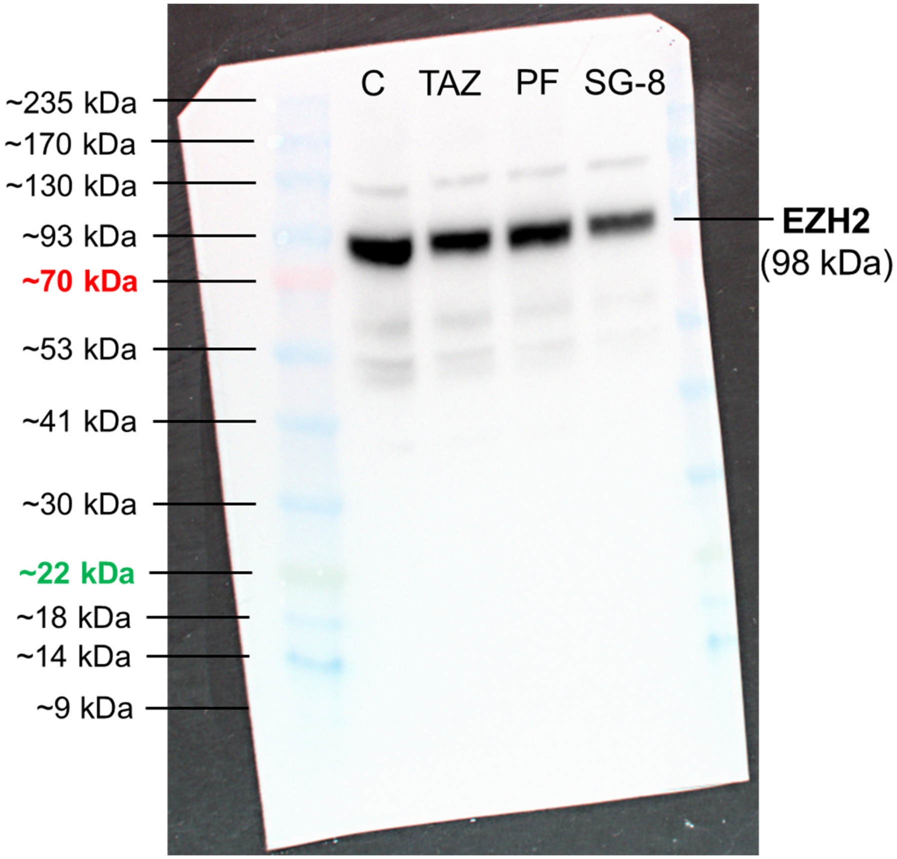

Figure S25. EZH2 (98 kDa) antibody.

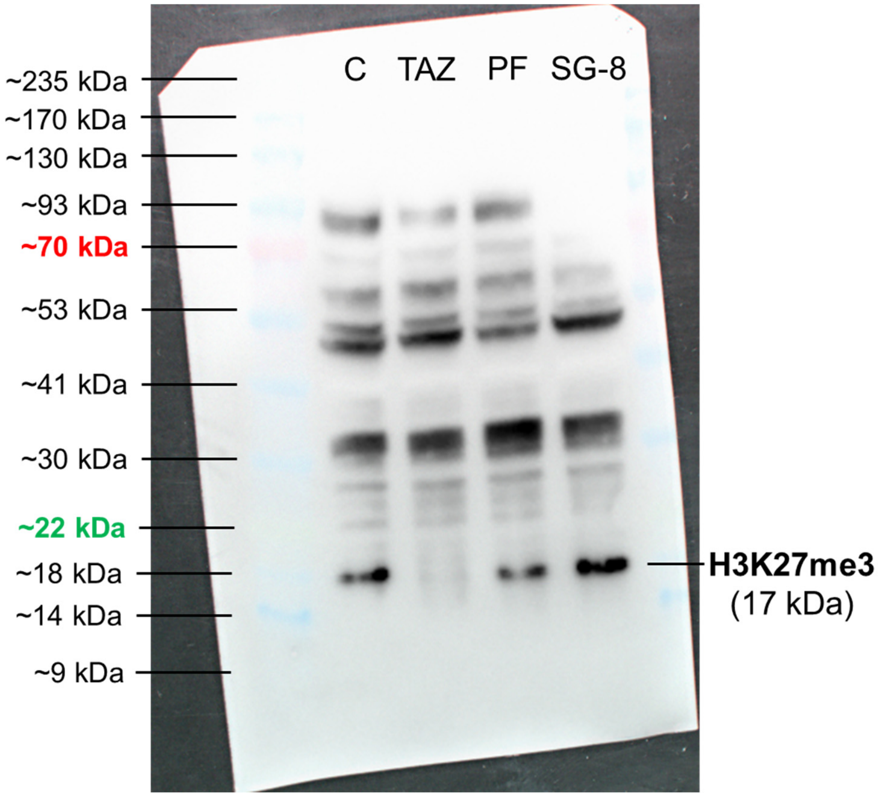

Figure S26. H3K27me3 (17 kDa) antibody.

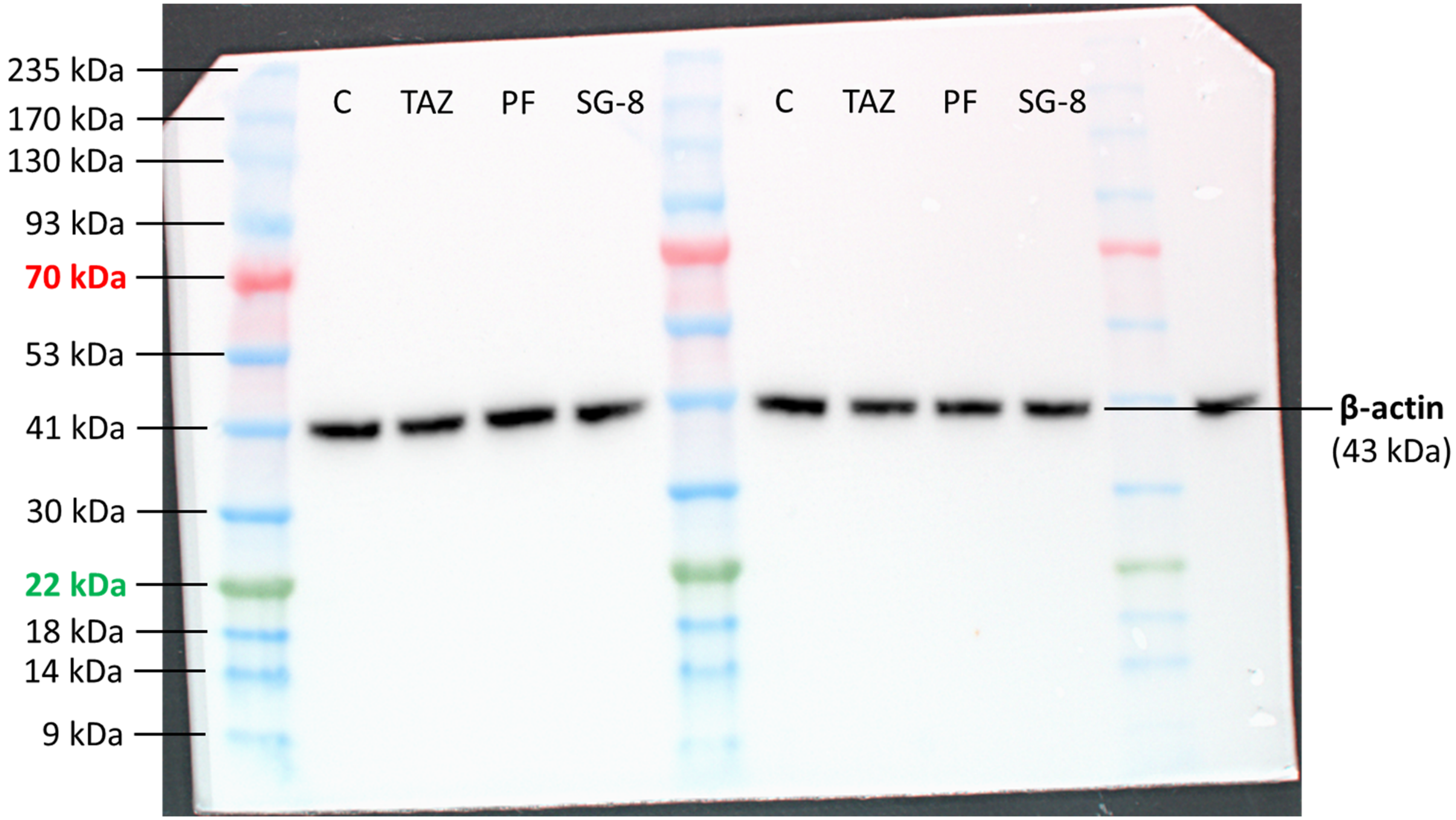

Figure S27. β-actin (43 kDa) antibody. C – Control, TAZ – Tazemetostat, PF – PF-06726304, SG-8 – 5,8-dichloro-2-[(3,5-dimethyl-1-oxo-1λ^5-pyridin-2-yl)methyl]-7-(3,5-dimethylisoxazol-4-yl)-3,4-dihydroisoquinolin-1(2*H*)-one.

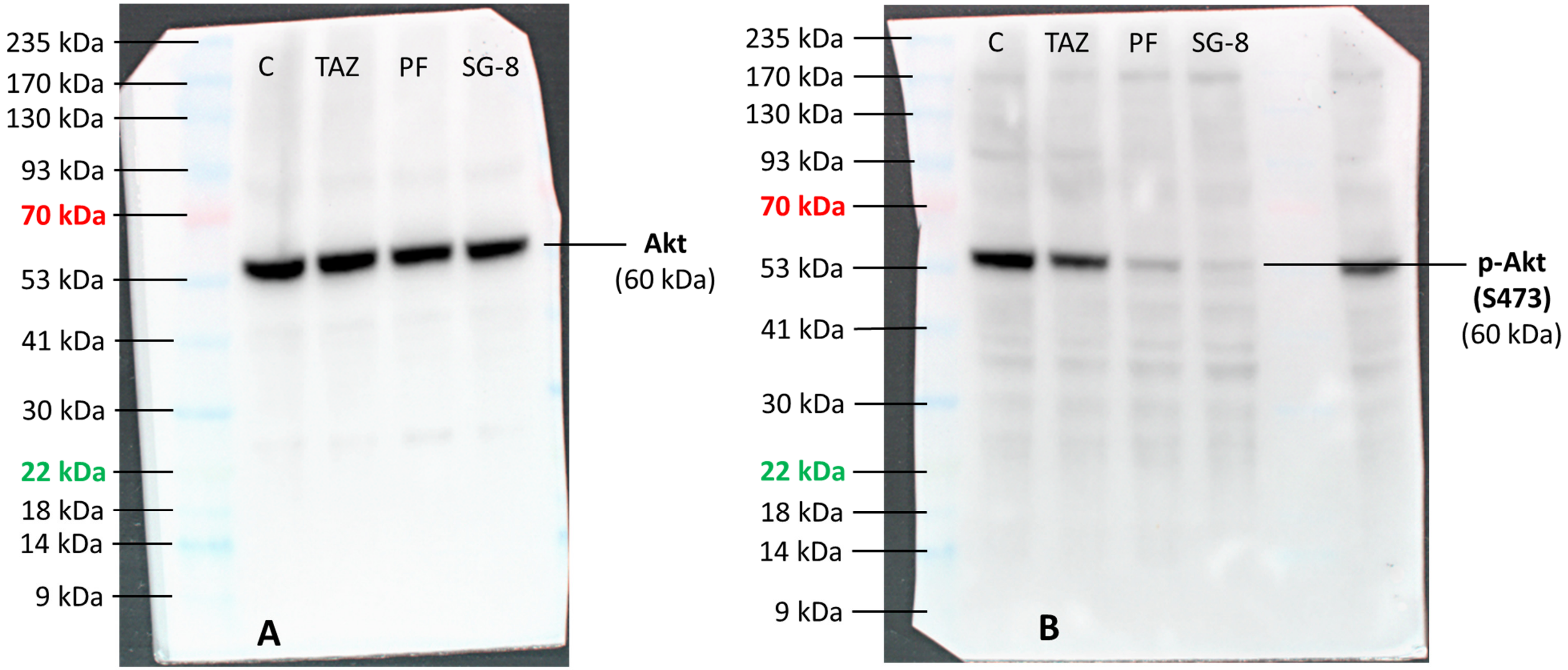

Figure S28. The initial membrane (**Figure S27**) was cut into two pieces (**A** and **B**). **A**: Akt (60 kDa) antibody. **B**: p-Akt (Ser473) (60 kDa) antibody.

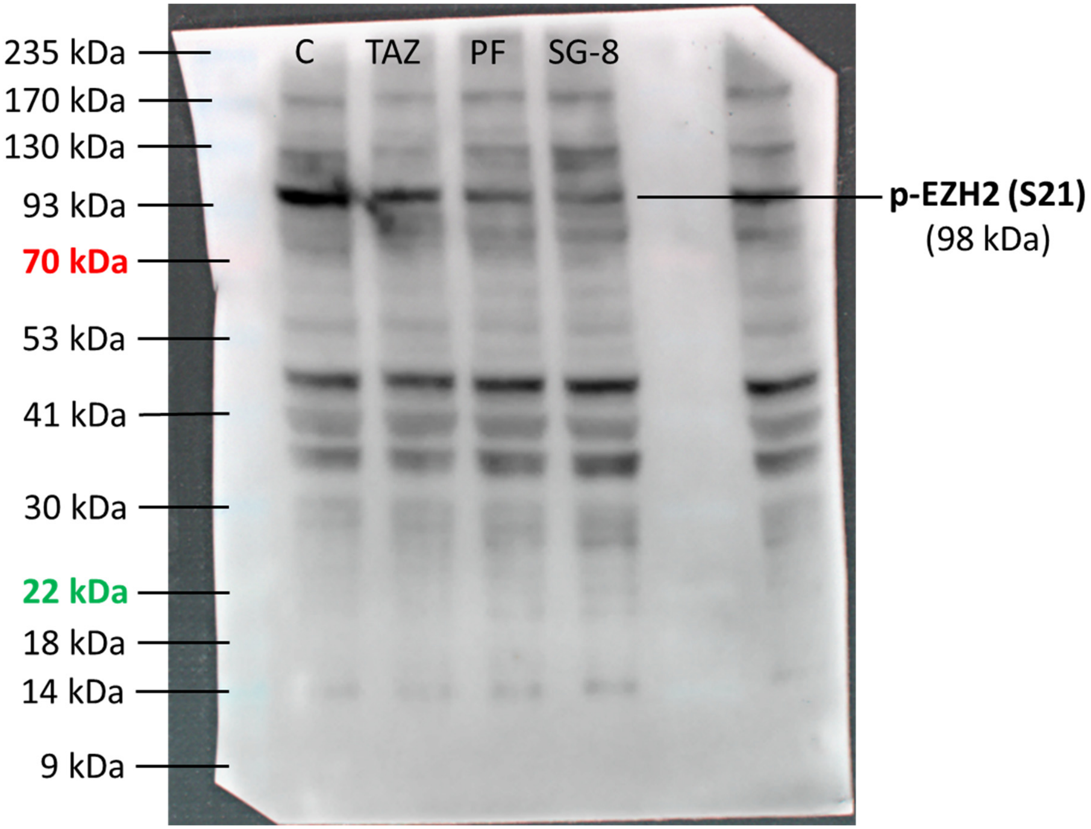

Figure S29. p-EZH2 (Ser21) (98 kDa) antibody.

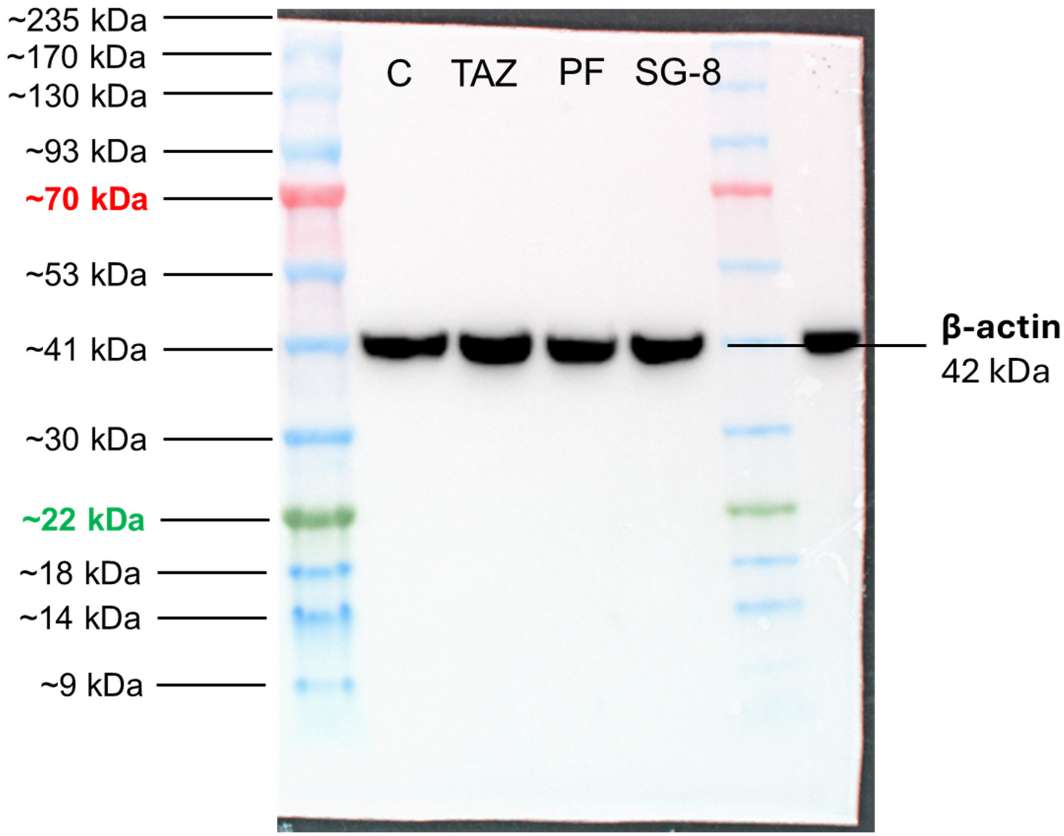

Figure S30. β-actin (42 kDa) antibody. C – Control, TAZ – Tazemetostat, PF – PF-06726304, SG-8 – 5,8-dichloro-2-[(3,5-dimethyl-1-oxo-1λ[5]-pyridin-2-yl)methyl]-7-(3,5-dimethylisoxazol-4-yl)-3,4-dihydroisoquinolin-1(2*H*)-one.

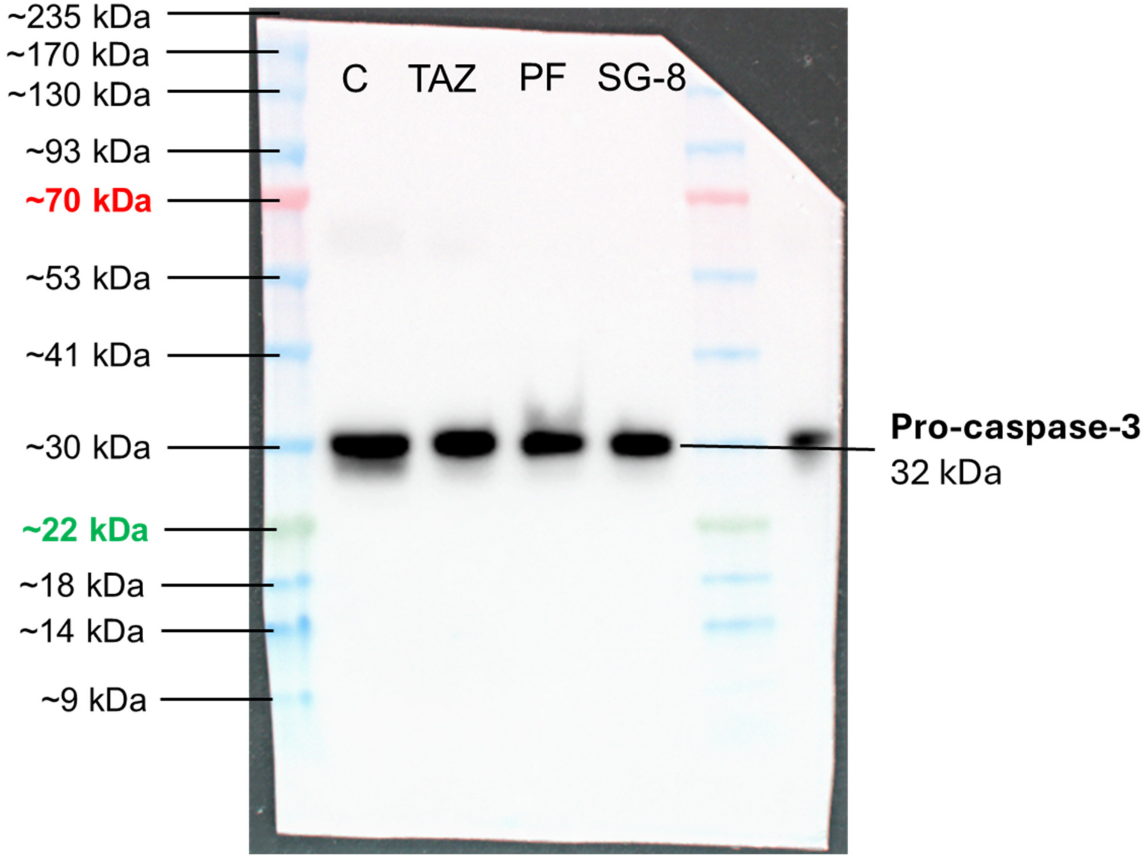

Figure S31. Caspase-3 (17, 19, 35 kDa) antibody.

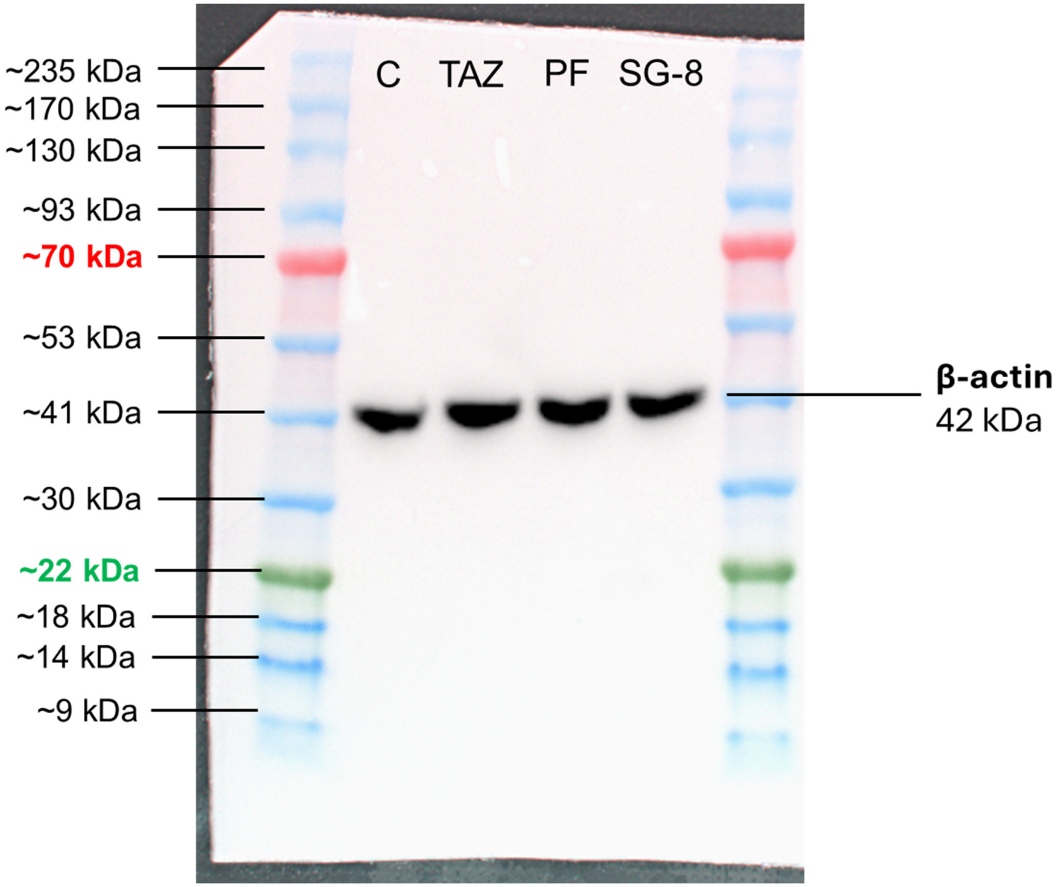

Figure S32. β-actin (42 kDa) antibody. C – Control, TAZ – Tazemetostat, PF – PF-06726304, SG-8 – 5,8-dichloro-2-[(3,5-dimethyl-1-oxo-1λ^5-pyridin-2-yl)methyl]-7-(3,5-dimethylisoxazol-4-yl)-3,4-dihydroisoquinolin-1(2*H*)-one.

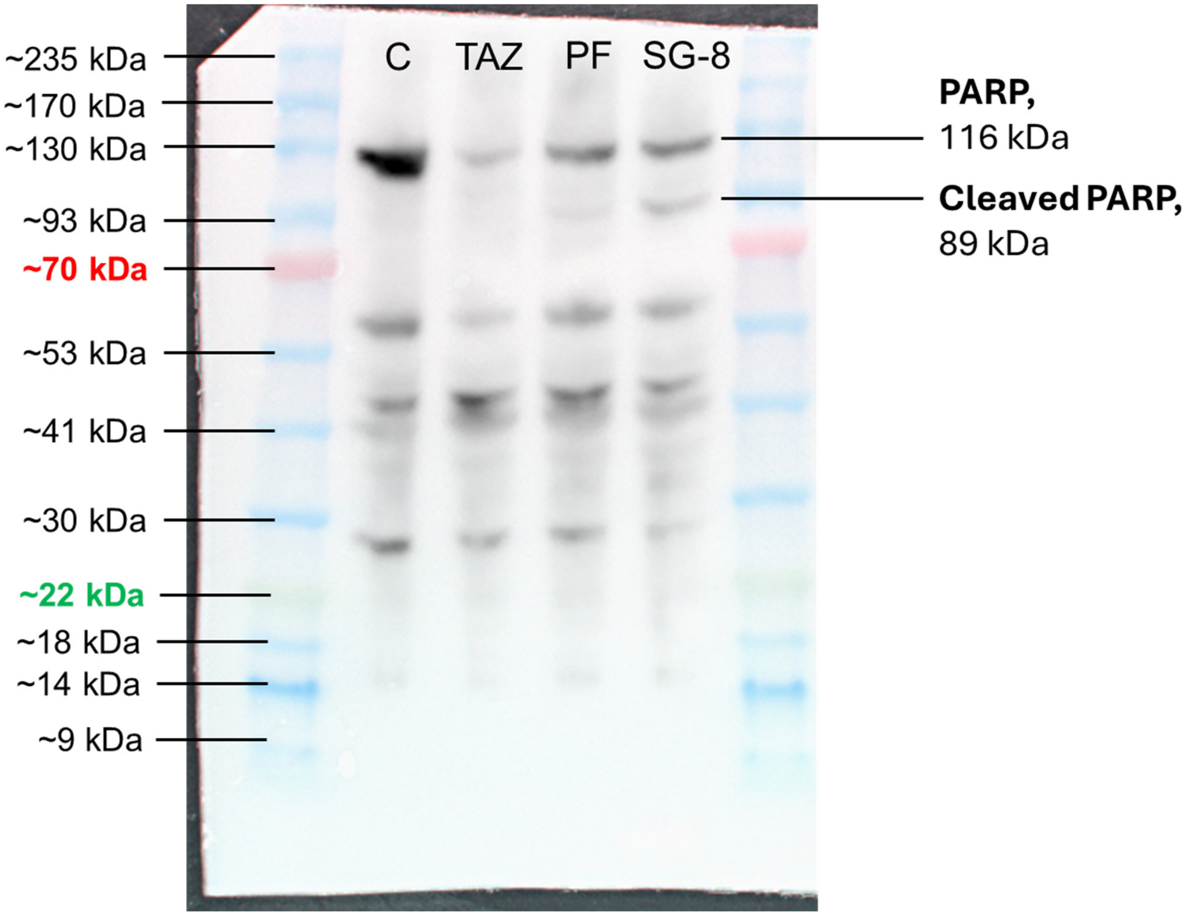

Figure S33. PARP (89, 116 kDa) antibody.

Flow Cytometry Data

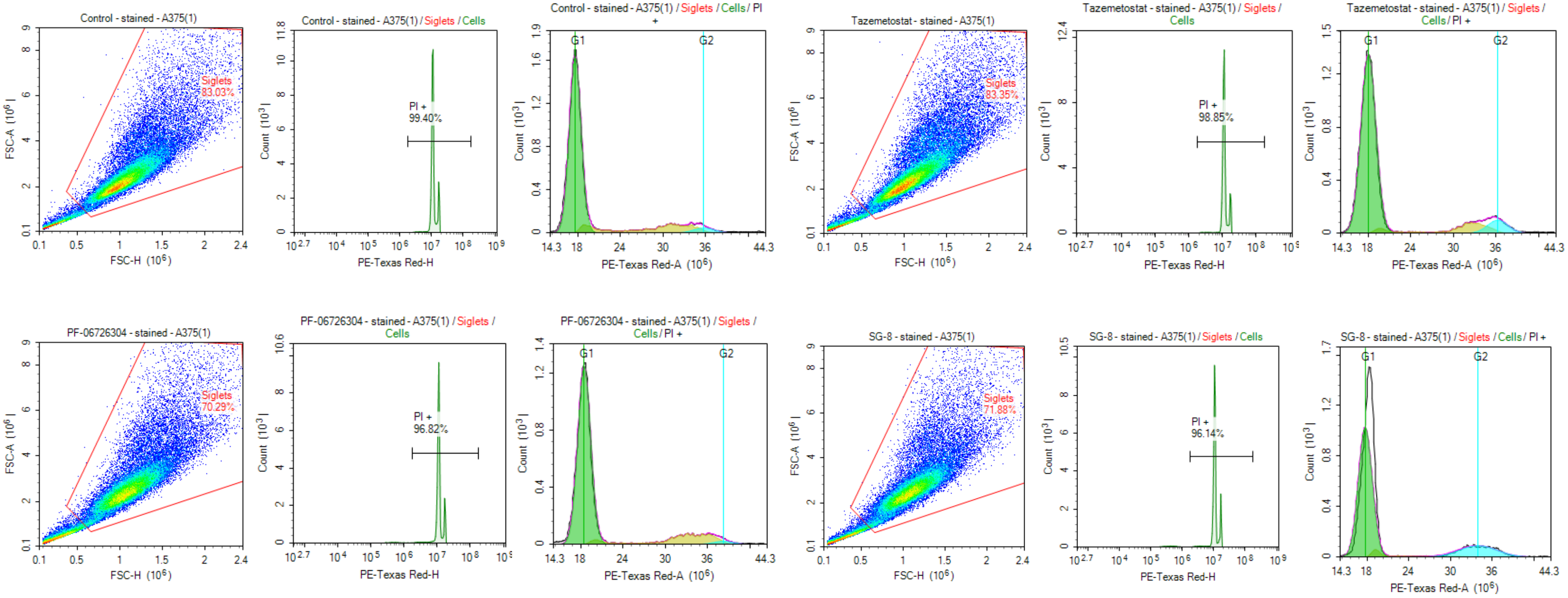

Figure S34. Flow cytometry data. A375 cells were stained with propidium iodide (PI).

Table S3. Cell cycle phase distributions (%).

	G_0/G_1	S	G_2/M
**Control**	81.63±2.14	16.66±0.56	1.77±0.01
**Tazemetostat**	78.92±1.54	16.23±1.06	4.88±0.02
**PF-06726304**	80.44±2.11	16.54±1.04	2.59±0.02
**SG-8**	73.94±1.17	11.17±0.23	13.10±0.10
